# Double Negative Control Inference in Test-Negative Design Studies of Vaccine Effectiveness

**Published:** 2022-03-23

**Authors:** Kendrick Qijun Li, Xu Shi, Wang Miao, Eric Tchetgen Tchetgen

**Affiliations:** Department of Biostatistics, University of Michigan;; Department of Biostatistics, University of Michigan;; Department of Probability and Statistics, Peking University;; Department of Statistics and Data Science, The Wharton School, University of Pennsylvania

**Keywords:** Causal inference, proximal causal inference, selection bias, unmeasured confounding

## Abstract

The test-negative design (TND) has become a standard approach to evaluate vaccine effectiveness against the risk of acquiring infectious diseases in real-world settings, such as Influenza, Rotavirus, Dengue fever, and more recently COVID-19. In a TND study, individuals who experience symptoms and seek care are recruited and tested for the infectious disease which defines cases and controls. Despite TND’s potential to reduce unobserved differences in healthcare seeking behavior (HSB) between vaccinated and unvaccinated subjects, it remains subject to various potential biases. First, residual confounding bias may remain due to unobserved HSB, occupation as healthcare worker, or previous infection history. Second, because selection into the TND sample is a common consequence of infection and HSB, collider stratification bias may exist when conditioning the analysis on testing, which further induces confounding by latent HSB. In this paper, we present a novel approach to identify and estimate vaccine effectiveness in the target population by carefully leveraging a pair of negative control exposure and outcome variables to account for potential hidden bias in TND studies. We illustrate our proposed method with extensive simulation and an application to study COVID-19 vaccine effectiveness using data from the University of Michigan Health System.

## 1 Introduction

### 1.1 Text-negative design studies of vaccine effectiveness

The test-negative design (TND) has become a standard approach to evaluate real-world vaccine effectiveness (VE) against the risk of acquiring infections diseases (Chung et al., 2020; Flannery et al., 2019; Jackson et al., 2017; Rolfes et al., 2019; Tenforde et al., 2021). In an outpatient Influenza VE test-negative design, for example, symptomatic individuals seeking care and meeting eligibility criteria are enrolled and their Influenza virus infection status is subsequently confirmed via a laboratory test. VE against flu infection is then measured by comparing the prevalence of vaccination between the test-positive “cases” and test-negative “controls” (Jackson and Nelson, 2013; Jackson et al., 2017). Besides Influenza, the TND and its variants have also been applied to study VE against pneumococcal disease (Broome, Facklam, and Fraser, 1980), dengue (Anders et al., 2018; Utarini et al., 2021), rotavirus (Boom et al., 2010; Schwartz et al., 2017), and other infectious diseases. Recently, the TND has increasingly been used in post-licensure evaluation of COVID-19 VE (Dagan et al., 2021; Dean, Hogan, and Schnitzer, 2021; Hitchings et al., 2021; Olson et al., 2022; Patel, Jackson, and Ferdinands, 2020; Thompson et al., 2021).

Test-negative designs are believed to reduce unmeasured confounding bias due to healthcare seeking behavior (HSB), whereby care seekers are more likely to be vaccinated, have healthier behaviors that reduces the risk of infection, and get tested when ill (Jackson et al., 2006; Shrank, Patrick, and Brookhart, 2011). By restricting analysis to care seekers who are tested for the infection in view (e.g. Influenza or COVID-19), the vaccinated and unvaccinated are more likely to share similar HSB and underlying health characteristics. Misclassification of infection status is also reduced because the analysis is restricted to tested individuals (Jackson and Nelson, 2013).

**Figure 1:**
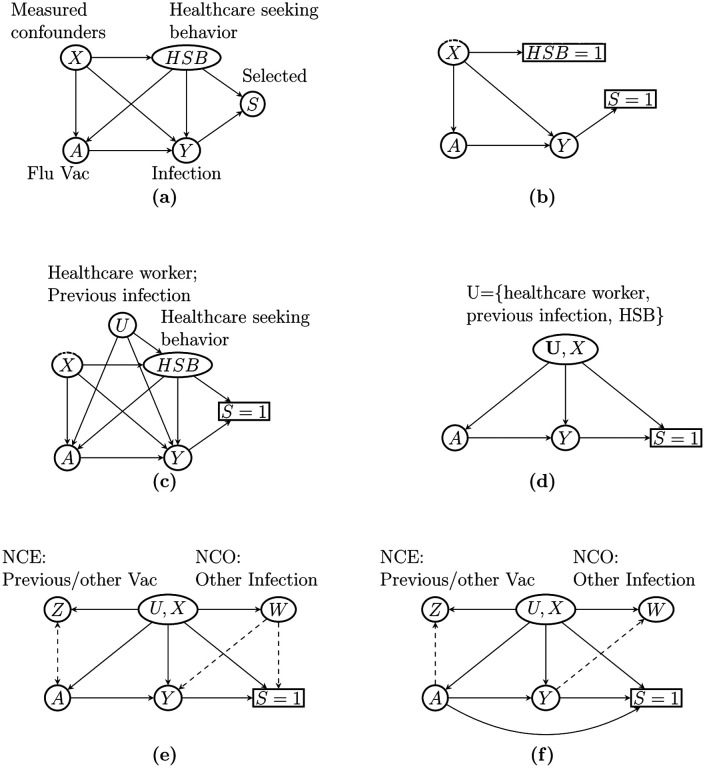
Causal relationships of variables in a test-negative design. Sullivan, Tchetgen Tchetgen, and Cowling, 2016 used (a) to illustrate the causal relationship between variables in a test-negative design in the general population, and used (b) to illustrate the assumption implicit in the common approach to estimate VE from the study data that study subjects have identical healthcare seeking behavior (HSB) (Sullivan, Tchetgen Tchetgen, and Cowling, 2016). (c) shows that if *HSB* remains partially unobserved, then the backdoor paths *A* ← *HSB* → *Y* and *A* ← *HSB* → *S* = 1 ← *Y* indicate unmeasured confounding bias and selection bias, respectively. Other unmeasured confounders, such as occupation as a healthcare worker and previous infection, open additional backdoor paths between *A* and *Y* and result in additional confounding bias. (d) shows a simplified DAG from (c) that combines the unmeasured confounders into a single variable *U*. (e) illustrates our approach to estimate VE leveraging negative control exposure *Z* and outcome *W*. Dashed arrows indicate effects that are not required. (f) shows a scenario with the *A* → *Y* arrow where the causal odds ratio can still be identified under additional assumptions.

Sullivan et al. (2016) used directed acyclic graphs (DAG) to illustrate the rationale behind TND in the context of evaluating VE against Influenza infection, as shown in Figures 1(a) and (b). We denote Influenza vaccination status by *A* and Influenza infection by *Y*, so that the arrow *A* → *Y* represents VE against flu infection. Selection into the TND study sample, denoted by *S*, is triggered by a subject experiencing flu-like symptoms or acute respiratory illness, seeking care at clinics or hospitals, and getting tested for Influenza infection, hence the *Y* → *S* edge. Healthcare seeking behavior, denoted by HSB, may affect *S*, *A*, and *Y* because subjects with certain health seeking proclivities may be more likely to seek care, take annual flu shots, and participate in healthy and preventative behaviors. The above variables are subject to effects of other clinical or demographic factors, such as age, season and high-risk conditions, included in Figure 1 as confounders *X*. The TND assumes that by restricting recruitment to care seekers, the study subjects have identical healthcare seeking behavior; in other words, conditioning the analysis on *S* = 1 necessarily leads to HSB= 1, which blocks the effects of HSB (Figure 1(b)). The effects of *X* are further adjusted for by including these factors in a logistic regression model or by inverse probability weighting (Bond, Sullivan, and Cowling, 2016; Thompson et al., 2021).

However, the TND remains subject to potential hidden bias. First, the assumption that all study subjects seeking care are lumped into a single category HSB= 1 may be unrealistic. It may be more realistic that HSB is not a deterministic function of *S* and remains a source of confounding bias even after conditioning on *S*. Furthermore, there might be other mismeasured or unmeasured confounders, denoted as *U*. For example, healthcare workers are at increased risk of flu infection due to higher exposure to flu patients and are more likely to seek care and receive vaccination due to health agency guidelines (Black et al., 2018). Previous flu infection history may also be a source of confounding if it alters the likelihood of vaccination and care seeking, while also providing immunity against circulating strains (Krammer, 2019; Sullivan, Tchetgen Tchetgen, and Cowling, 2016). These unmeasured or mismeasured potential sources of confounding, if not properly accounted for, can result in additional confounding bias, as illustrated in Figure 1(c). Finally, collider stratification bias is likely present due to conditioning on *S*, which is a common consequence of HSB, other risk factors (*X*, *U*), and Influenza infection *Y* (Lipsitch, Jha, and Simonsen, 2016). That is, conditioning on *S* unblocks the backdoor path *A* ← (*X*, *U*, *HSB*) → *S* ← *Y*, which would in principle be blocked if study subjects had identical levels of HSB and other risk factors (Sullivan, Tchetgen Tchetgen, and Cowling, 2016).

Accounting for these potential sources of bias is well known to be challenging, and potentially infeasible without additional assumptions or data. This can be seen in Figure 1(d), which is a simplified version of Figure 1(c) where the unmeasured confounders *U* include individuals’ occupation as a healthcare worker, previous flu infection, HSB, and so on. Figure 1(d) indicates that the unmeasured confounders *U* induce both confounding bias through the path *A* ← *U* → *Y* and collider stratification bias through the path *A* ← *U* → *S* ← *Y*. In presence of both unmeasured confounding and collider bias, causal bounds may be available (Gabriel, Sachs, and Sjölander, 2020) but likely too wide to be informative; causal identification in TND therefore remains to date an important and outstanding open problem in the causal inference literature which we aim to resolve.

### 1.2 Negative control methods

In recent years, negative control variables have emerged as powerful tools to detect, reduce and potentially correct for unmeasured confounding bias (Lipsitch, Tchetgen Tchetgen, and Cohen, 2010; Miao, Geng, and Tchetgen Tchetgen, 2018; Shi, Miao, and Tchetgen Tchetgen, 2020). The framework requires that at least one of two types of negative control variables are available which are *a priori* known to satisfy certain conditions: a negative control exposure (NCE) known to have no direct effect on the primary outcome; or a negative control outcome (NCO), known not to be an effect of the primary exposure. Such negative control variables are only valid and therefore useful to address unmeasured confounding in a given setting to the extent that they are subject to the same source of confounding as the exposure-outcome relationship of primary interest. Thus, the observed association between a valid NCE and the primary outcome (conditional on the primary treatment and observed covariates) or that between a valid NCO and the primary exposure can indicate the presence of residual confounding bias. For example, in a cohort study to investigate flu VE against hospitalization and death among seniors, to detect the presence of confounding bias due to underlying health characteristics, Jackson et al. (2006) used hospitalization/death before and after the flu season as NCOs and found that the association between flu vaccination and hospitalization was virtually the same before and during the flu season, suggesting that the lower hospitalization rate observed among vaccinated seniors versus unvaccinated seniors was partially due to healthy user bias.

Recently, new causal methods have been developed to not only detect residual confounding when present, but also to potentially de-bias an observational estimate of a treatment causal effect in the presence of unmeasured confounders when both an NCE and an NCO are available, referred to as the double negative control (Miao, Geng, and Tchetgen Tchetgen, 2018; Shi et al., 2020; Tchetgen Tchetgen et al., 2020). In this recent body of work, the double negative control design was extended in several important directions including settings in which proxies of treatment and outcome confounding routinely measured in well designed observational studies may be used as negative control variables, a framework termed *proximal causal inference*; longitudinal settings where one is interested in the joint effects of time-varying exposures (Ying et al., 2021), potentially subject to both measured and unmeasured confounding by time-varying factors; and in settings where one aims to estimate direct and indirect effects in mediation analysis subject to unmeasured confounding or unmeasured mediators (Dukes, Shpitser, and Tchetgen Tchetgen, 2021; Ghassami, Shpitser, and Tchetgen Tchetgen, 2021). Additional recent papers in this fast-growing literature include Qi, Miao, and Zhang (2021), Liu and Tchetgen Tchetgen (2021), Egami and Tchetgen Tchetgen (2021), Kallus, Mao, and Uehara (2021), Imbens, Kallus, and Mao (2021), Deaner (2018), Deaner (2021), Ghassami et al. (2021), Mastouri et al. (2021) and Ghassami, Shpitser, and Tchetgen Tchetgen (2022). Importantly, existing identification results in negative control and proximal causal inference literature has been restricted to i.i.d settings (Miao, Shi, and Tchetgen Tchetgen, 2018) and time series settings (Shi et al., 2021), and to date, to the best of our knowledge, outcome-dependent sampling settings such as TND, have not been considered, particularly one where confounding and selection bias might co-exist.

### 1.3 Outline

The rest of the paper is organized as followed: we introduce notation and the identification challenge in view in Sections 2.1. Next we develop the identification strategy and describe a new debiased estimator under a double negative control TND study in Section 2.2–2.4, assuming (1) homogeneous VE across strata defined by all measured and unmeasured confounders and (2) no direct effect of vaccination on selection into the TND sample. In Section 2.5, we relax the homogeneous VE assumption and describe identification and estimation allowing for VE to depend on observed covariates. In Section 2.6, we relax the assumption of no direct effect of vaccination on selection and introduce the assumptions under which our VE estimator is unbiased on the odds ratio scale. In Section 3, we demonstrate the performance of our method with simulation. In Section 4, the approach is further illustrated in an application to estimate COVID-19 VE against infection in a TND study nested within electronic health records from University of Michigan Health System. We then conclude with a discussion in Section 5.

## 2 Method

### 2.1 Preliminary: estimation under no unmeasured confounding and no selection bias

To fix ideas, we first review estimation assuming all confounders (*U*, *X*) are fully observed and the study sample is randomly drawn (rather than selected by testing) from source population, referred to as the “target population”. That is, we observe data on (*A*, *Y*, *U*, *X*) which are independent and identically distributed in the target population. For each individual, we write *Y* (*a*) as the binary potential infection outcome had, possibly contrary to fact, the person’s vaccination status been *A* = *a*, *a* = 0, 1. Our goal is to provide identification and estimation strategies for the causal risk ratio (RR) defined as *RR* = *E*[*Y* (1)]/*E*[*Y* (0)]. Let *β*_0_ denote the log causal RR, i.e., *RR* = exp(*β*_0_). Following Hudgens and Halloran (2006) and Struchiner and Halloran (2007), we define VE as one minus the causal RR: *V E* = 1 − *RR* = 1 − exp(*β*_0_). The potential outcomes and the observed data are related through the following assumptions:

**Assumption 1**. *(Identification conditions of mean potential outcomes)*.

- *(Consistency) Y* (*a*) = *Y if A* = *a almost surely for a* = 0, 1;
- *(Exchangeability) A* ⫫ *Y* (*a*)|*U*, *X for a* = 0, 1.
- *(Positivity)* 0 < *P*(*A* = *a*|*U*, *X*) *almost surely a* = 0, 1.

Assumption 1(a) states that the infection status of a subject with vaccination status *A* = *a* is equal to the corresponding potential outcome *Y* (*a*). This further requires that the treatment is sufficiently well-defined and a subject’s potential outcome is not affected by the treatment of other subjects (Cole and Frangakis, 2009). Assumption 1(b) states that treatment is exchangeable within strata of (*U*, *X*), i.e. there is no unmeasured confounding given (*U*, *X*). We develop methods that allow *U* to be unmeasured in Section 2.3. Assumption 1(c) states that for all realized values of (*U*, *X*) there is at least one individual with an opportunity to get treatment *a* = 0, 1.

Let *Q*(*A* = *a*, *U*, *X*) = 1/*P*(*A* = *a*|*U*, *X*) denote the inverse of the probability of vaccination status *A* = *a* given confounders (Rosenbaum, 1987). Under Assumption 1, it is well known that, if *U* were observed, the mean potential outcome for treatment *a* in the general population can be identified by inverse probability of treatment weighting (IPTW):

(1) E[Y(a)]=E[I(A=a)Q(A=a,U,X)Y],

for *a* = 0, 1. Therefore, the log causal RR *β*_0_ satisfies the following equation

$$E\left[Q(A=1,U,X)AY\text{exp}\left(-\beta_{0}\right)\right]-E[Q(A=0,U,X)(1-A)Y]=0.$$

Equivalently, we have

(2) $$E\left[V_{0}\left(A,Y,U,X;\beta_{0}\right)\right]=0$$

for unbiased estimation, where *V*_0_(*A*, *Y*, *U*, *X*; *β*) = (−1)^1−*A*^*Q*(*A*, *U*, *X*)*Y* exp(−*βA*).

### 2.2 Tackling selection bias under a semiparametric risk model

Next, consider a TND study for which data (*A*, *Y*, *X*, *U*) is observed only for the tested individuals with *S* = 1. Because *S* is impacted by other factors such as infection, the estimating function *V*_0_(*A*, *Y*, *U*, *X*; *β*_0_) may not be unbiased with respect to the study sample; i.e. *V*_0_(*A*, *Y*, *U*, *X*; *β*_0_)|*U*, *X*, *S* = 1] ≠ 0 without another assumption about the selection process into the TND sample.

For a study sample of size *n* from a TND, we denote the *i*-th study subject’s variables as (*A*_*i*_, *Y*_*i*_, *U*_*i*_, *X*_*i*_), *i* = 1, …, *n*. For generalizability, we first make the key assumption that vaccination *A* is unrelated to selection *S* other than through a subject’s infection status *Y* and confounders (*U*, *X*).

**Assumption 2** (Treatment-independent sampling). *S* ⫫ *A*|*Y*, *U*, *X*.

In the test-negative design, this assumption requires that an individual’s decision to seek care and get tested only depends on the presence of symptoms and his/her underlying behavioral or socioeconomic characteristics, including HSB (contained in (*U*, *X*)); a person’s vaccination status does not directly Influence their selection process. The DAGs in Figure 1(a)–(e) in fact encode this conditional independence condition. We will relax this assumption in Section 2.6.

**Assumption 3** (No effect modification by a latent confounder). *For a* = 0, 1,

(3) $$P(Y=1|A=a,U,X)=\text{exp}\left(\beta_{0}a\right)g(U,X)$$

*where g*(*U*, *X*) *is an unknown function only restricted by* 0 ≤ *P*(*Y* = 1|*A*, *U*, *X*) ≤ 1.

Assumption 3 defines a semiparametric multiplicative risk model which posits that vaccine effectiveness, measured on the RR scale, is constant across (*U*, *X*) strata in the target population. In other words, the effect of vaccination *A* on the risk of infection *Y* is not modified by confounders *U*, *X*. In Section 2.5, we will relax the assumption to allow for effect modification by measured confounders *X*. Infection risk for control subjects *P*(*Y* = 1|*A* = 0, *U*, *X*) = *g*(*U*, *X*) is left unspecified and thus defines the nonparametric component of the model.

Under Assumptions 1 and 3, one can verify that exp(*β*_0_) = *E*[*Y* (1)]/*E*[*Y* (0)], which is the marginal causal RR. The potential infection outcome means *E*[*Y* (1)] and *E*[*Y* (0)] in the target population cannot be identified due to the study selection process without an additional restriction. Nevertheless, the estimating equation (2) implies that it may still be possible to identify *β*_0_ without necessarily identifying *E*[*Y* (0)] and *E*[*Y* (1)]. The following proposition indicates that the same is true when the data are subject to selection bias of certain structure.

**Proposition 1**. *Under* Assumptions 1–3, *the parameter β*_0_ *satisfies*

(4) $$E\left[V_{0}\left(A,Y,U,X;\beta_{0}\right)|U,X,S=1\right]=0.$$

The proof of Proposition 1 is in Appendix A. From Proposition 1, the IPTW estimating function *V*_0_ derived from the target population is also unbiased with respect to the study sample.

Under Assumptions 1–3, one can estimate *β*_0_ with $\hat{\beta }$ as the solution to

(5) $$\frac{1}{n}\sum_{i=1}^{n}{\left(-1\right)}^{1-A_{i}}c\left(X_{i}\right)\hat{Q}\left(A_{i},U_{i},X_{i}\right)Y_{i}\text{exp}\left(-{\hat{\beta }}_{0}A_{i}\right)=0,$$

where *n* is the size of the selected sample, *c*(·) is a user specified function, and $\hat{Q}\left(A_{i},U_{i},X_{i}\right)=\hat{P}\left(A=A_{i}|U_{i},X_{i}\right)$ is the estimated probability of have vaccination status *A* = *A*_*i*_ given confounders (*U*_*i*_, *X*_*i*_). Letting *c*(*X*_*i*_) = 1, the resulting estimator

$${\hat{\beta }}_{0}=\left[\sum_{i=1}^{n}\hat{Q}\left(A_{i},U_{i},X_{i}\right)A_{i}Y_{i}\right]/\left[\sum_{i=1}^{n}\hat{Q}\left(A_{i},U_{i},X_{i}\right)\left(1-A_{i}\right)Y_{i}\right]$$

is essentially the IPTW estimator of marginal RR in Schnitzer (2022) assuming (*U*_*i*_, *X*_*i*_)’s are all observed.

However, *Q*(*A*, *U*, *X*) cannot be estimated because *U* is unobserved. Furthermore, even if *U* were observed, $\hat{Q}\left(A_{i},U_{i},X_{i}\right)$ may not be identified from the TND sample due to selection bias. In the next section, we describe a new framework to account for unmeasured confounding in a TND setting, leveraging negative control exposure and outcome variables.

### 2.3 Tackling unmeasured confounding bias leveraging negative controls

#### 2.3.1 Negative control exposure (NCE) and treatment confounding bridge function

As shown in Figure 1(e), suppose that one has observed a valid possibly vector-valued NCE, denoted as *Z*, which is a priori known to satisfy the following key independence conditions:

**Assumption 4** (NCE independence conditions). *Z* ⫫ (*Y*, *S*)|*A*, *U*, *X*.

Assumption 4 essentially states that any existing *Z* − *Y* association conditional on (*X*, *A*) in the target population must be a consequence of their respective association with *U*, therefore indicating the presence of confounding bias. Importantly, the NCE must a priori be known to have no causal effect on infection status (Miao, Shi, and Tchetgen Tchetgen, 2018). Likewise, the association between *Z* and *S* conditional on (*X*, *A*) is completely due to their respective association with *U*. Figure 1(e) presents a graphical illustration of an NCE that satisfies Assumption 4.

In the Influenza VE setting, a candidate NCE can be vaccination status for the preceding year, or other vaccination status such as Tdap (Tetanus, Diphtheria, Pertussis) vaccine, as both are known to effectively provide no protection against the circulating flu strain in a given year. We now provide an intuitive description of our approach to leverage *Z* as an imperfect proxy of *U* for identification despite being unable to directly observe *U*.

To illustrate the rationale behind identification, ignore selection bias for now and suppose that *Q*(*A*, *U*) = *α*_0_ + *α*_1_*A* + *α*_2_*U*, suppressing measured confounders *X*. Although *U* is unobserved, suppose further that *Z* satisfies *E*[*Z*|*A*, *U*] = *γ*_0_ + *γ*_1_*A* + *γ*_2_*U*. Then we have that

$$Q(A,U)=E[q(A,Z)|A,U],\;U=E[\tilde{U}(A,Z)|A,U],$$

where $\tilde{U}=\left(Z-\gamma_{0}-\gamma_{1}A\right)/\gamma_{2}$. Replacing *U* with $\tilde{U}$ in *Q*(*A*, *U*), we get $q(A,Z)=\alpha_{0}+\alpha_{1}A+\alpha_{2}\tilde{U}(A,Z)$, which does not depend on unmeasured confounder *U* and can represent the inverse probability of vaccination as *Q*(*A*, *U*) = *E*[*q*(*A*, *Z*)|*A*, *U*]. If all parameters of *q* were known, it would naturally follow that the IPTW method in (1) can be recovered by

$$E[Y(a)]=E\{I(A=a)E[q(A,Z)|A,U]Y\}\overset{A.4}{=}E[I(A=a)q(A,Z)Y],$$

Therefore, *β*_0_ can be identified if the distribution of (*A*, *Y*, *Z*) in the target population is available provided that parameters indexing *q* can be identified.

The above insight motivates the following assumption:

**Assumption 5** (treatment confounding bridge function). *There exists a function q*(*A*, *Z*, *X*) *that satisfies*, *for every a*, *u and x*,

(6) Q(A=a,U=u,X=x)=E[q(A,Z,X)∣A=a,U=u,X=x]

The function *q* that satisfies (6) is called a treatment confounding bridge function, as it bridges the observed NCE with the unobserved propensity score (Cui et al., 2020). Below we give two examples where the integral equation (6) can easily be solved and the treatment confounding bridge function *q* admits a closed form solution.

**Example 1**. *(Binary U and Z) Suppose that U is binary*, *and so is the NCE Z*. *For simplicity we suppress X*. *The integral* equation (6) *can then be written as* $\sum_{z=0}^{1}q(a,z)P(Z=z|U=u,A=a)=P{\left(A=a|U=u\right)}^{-1}$, *or equivalently*, $\sum_{z=0}^{1}p_{za.u}q(a,z)=1$ *for each a*, *u* ∈ {0, 1}, *where p*_*za*.*u*_ = *P*(*Z* = *z*, *A* = *a*|*U* = *u*). *Therefore*, *the treatment confounding bridge function q*(*a*, *z*) *solves the linear equation system*

$$P_{Z,A|U}\left(\begin{array}{c} q(a,0)\\ q(a,1) \end{array}\right)=\left(\begin{array}{c} 1\\ 1 \end{array}\right),\;\text{where }P_{Z,A|U}=\left(\begin{array}{cc} p_{0a.0} & p_{1a.0}\\ p_{0a.1} & p_{1a.1} \end{array}\right).$$

*If the matrix P*_*Z*,*A*|*U*_ *is invertible*, *then q*(*a*, *z*) *has a closed form solution given by*

(7) $$q(a,z)=\left[p_{1a.1}-p_{1a.0}+\left(p_{0a.0}-p_{0a.1}-p_{1a.1}+p_{1a.0}\right)z\right]/\left(p_{0a.0}p_{1a.1}-p_{0a.1}p_{1a.0}\right).$$

*The result can be extended to the cases where Z is polytomous as detailed in* Appendix D.

**Example 2**. *(Continuous U and Z) Suppose the unmeasured confounder U and the NCE Z are continuous*. *Further assume that*

$$\begin{array}{l} A|U,X\sim \text{Bernoulli}\left({\left[1+\text{exp}\left(-\mu_{0A}-\mu_{UA}U-\mu_{XA}X\right)\right]}^{-1}\right)\\ Z|A,U,X\sim N\left(\mu_{0Z}+\mu_{AZ}A+\mu_{UZ}U+\mu_{XZ}X,\sigma_{Z}^{2}\right). \end{array}$$

*By the derivation in* Appendix E, *the treatment confounding bridge function q*(*A*, *Z*, *X*) *is*

(8) $$q(A,Z,X)=1+\text{exp}\left[{\left(-1\right)}^{A}\left(\tau_{0}+\tau_{1}A+\tau_{2}Z+\tau_{3}X\right)\right]$$

*where $\tau_{0}=\mu_{0A}-\frac{\mu_{UA}\mu_{0Z}}{\mu_{UZ}}-\frac{\sigma_{z}^{2}\mu_{UA}^{2}}{2\mu_{UZ}^{2}}$*, $\tau_{1}=\frac{\sigma_{z}^{2}\mu_{UA}^{2}}{\mu_{UZ}^{2}}-\frac{\mu_{UA}\mu_{AZ}}{\mu_{UZ}}$, *τ*_2_ = *μ*_*UA*_/*μ*_*UZ*_, *and τ*_3_ = *μ*_*XA*_ − *μ*_*XZ*_*μ*_*UA*_/*μ*_*UZ*_.

Formally, Equation (6) defines a Fredholm integral equation of the first kind, with treatment confounding bridge function *q*(*A*, *Z*, *X*) as its solution (Cui et al., 2020). Heuristically, the existence of a treatment confounding bridge function requires that variation in *Z* induced by *U* is sufficiently correlated with variation in *A* induced by *U*. For instance, in Example 1, existence of a treatment confounding bridge function requires that the matrix *P*_*Z*,*A*|*U*_ is nonsingular. In Example 2, the existence of a treatment confounding bridge function amounts to the condition *μ*_*UZ*_ ≠ 0, which again requires Z⫫U|A,X. Cui et al. (2020) provided formal conditions sufficient for the existence of the treatment confounding bridge function satisfying Equation (6). These conditions are reproduced for completeness in Appendix B.

Thus, under Assumption 5, we propose to construct a new unbiased estimating function for *β*_0_ by replacing *Q*(*A*, *U*, *X*) with *q*(*A*, *Z*, *X*) in *V*_0_(*A*, *Y*, *U*, *X*; *β*_0_).

**Theorem 1**. *(Moment restriction of β*_0_*) Under* Assumptions 1–5, *we have that*

$$E\left[V_{1}\left(A,Y,Z,X;\beta_{0}\right)|U,X,S=1\right]=0$$

*where V*_1_(*A*, *Y*, *Z*, *X*; *β*_0_) = (−1)^1−*A*^*q*(*A*, *Z*, *X*)*Y* exp(−*β*_0_*A*).

The proof of Theorem 1 is in Appendix C. In practice, if one can consistently estimate the treatment confounding bridge function *q*(*A*, *Z*, *X*) with $\hat{q}(A,Z,X)$, Theorem 1 suggests estimating *β*_0_ by solving the estimating equation

(9) $$\frac{1}{n}\sum_{i=1}^{n}{\left(-1\right)}^{1-A_{i}}c\left(X_{i}\right)\hat{q}\left(A_{i},Z_{i},X_{i}\right)Y_{i}\text{exp}\left(-\beta_{0}A_{i}\right)=0,$$

which, for a one-dimensional *c*(·), results in a closed form estimator

$${\hat{\beta }}_{0}=\text{log}\left(\frac{\sum c\left(X_{i}\right)\hat{q}\left(A_{i},Z_{i},X_{i}\right)A_{i}Y_{i}}{\sum c\left(X_{i}\right)\hat{q}\left(A_{i},Z_{i},X_{i}\right)\left(1-A_{i}\right)Y_{i}}\right).$$

Importantly, although (6) may not have a unique solution, any solution uniquely identifies the causal log RR *β*_0_ for a fixed function *c*(·). Furthermore, although the choice of *c*(·) doesn’t change the unbiasedness of (9), it impacts the validity and efficiency of the resulting estimator ${\hat{\beta }}_{0}$. For example, (9) doesn’t lead to a estimator of *β*_0_ if *c*(*X*_*i*_) is set to be zero. In practice, one may simply set *c*(*X*_*i*_) ≡ 1. The result in Theorem 1 cannot directly be applied in practice because the treatment confounding bridge function is not identifiable even if random samples from the target population were available – solving (6) requires additional information about *U* which is unobserved. For instance, in Example 1 one is unable to directly estimate *q*(*a*, *z*) because *p*_*za*.*u*_ in (7) cannot directly be estimated from the observed data.

#### 2.3.2 Negative control outcome (NCO) for identification of treatment confounding bridge function

For identification and estimation of *q*, we leverage negative control outcomes (NCO) to construct feasible estimating equations for the treatment confounding bridge function as in Cui et al. (2020). Similar to NCEs, NCOs can be viewed as imperfect proxies of *U*. However, unlike NCEs, a valid NCO, denoted by *W*, is a measured covariate which is (i) known a priori not to be a causal effect of either the primary exposure *A* nor the NCE *Z*; and (ii) is associated with (*A*, *Z*) conditional on *X* only to the extent that it is associated with *U*.

Formally, we make the following assumption.

**Assumption 6**. *(NCO Independence Conditions)*.


$$\begin{array}{ll} \text{ (a) }W⫫A|U,X\text{; } & \text{ (b) }W⫫Z|A,U,X,Y\text{; }\\ \text{ (c) }S⫫Z|A,U,X,W,Y\text{. } \end{array}$$

Assumptions 6(a) and (b) formalize the requirement that neither the primary exposure nor NCE have direct effects on the NCO. Assumption 6(c) complements Assumption 4 and states that conditioning on *W* in addition to (*A*, *U*, *X*, *Y* ) does not alter the conditional independence of *Z* with *S*. In flu VE studies, a candidate NCO can be an infection whose risk is not causally affected by either *A* or *Z*. For example, if the NCE is selected to be Tdap vaccination, then a potential NCO may be current-year respiratory syncytial virus infection, as its risk is unlikely to be affected by Influenza or Tdap vaccination. Recent outpatient visits for other acute illnesses can also serve as NCO, such as blepharitis, wrist/hand sprain, lipoma, ingrowing nail, etc. (Leung et al., 2011). Figure 1(e) illustrates an NCO *W* that satisfies Assumptions 6(a) and (b).

Similar to Cui et al. (2020), we leverage the availability of an NCO as an additional proxy to identify the treatment confounding bridge function. However, a complication arises due to lack of a random sample from the target population, a key requirement in the approach outlined in Cui et al. (2020). In general, it is not possible to obtain sufficient information about neither the distribution of *W* nor that of *U* in the target population from the TND data without an additional structural assumption (Bareinboim and Pearl, 2012). In the following, we avoid imposing such an additional structural assumption by leveraging an important feature of infectious diseases such as Influenza and COVID-19; mainly that contracting such an infection is a rare event in most target populations of interest, and therefore information from the target population that is relevant for estimating the treatment confounding bridge function can be recovered from the test-negative control group. Formally, we make the following rare disease assumption.

**Assumption 7** (Rare infection). *There exist a small positive number δ >* 0 *such that*

(10) P(Y=1∣A=a,W=w,U=u,X=x)≤δ,  for almost every a,w,u,x

Assumption 7 states that infected subjects, whether vaccinated or not and regardless of their negative control outcomes, only constitute a small proportion of each (*U*, *X*) stratum in the general population; specifically, the assumption implies that $\frac{1}{1-\delta }\leq \frac{P(A,Z|U=u,X=x,Y=0)}{P(A,Z|U=u,X=x)}\leq 1-\delta$. Thus, under Assumptions 2, 4 and 7, *P*(*A* = *a*, *Z* = *z*|*U* = *u*, *X* = *x*) ≈ *P*(*A* = *a*, *Z* = *z*|*U* = *u*, *X* = *x*, *Y* = 0, *S* = 1) for all *a*, *z*, *x*, *u*. We now introduce a key property of the treatment confounding bridge function in Theorem 2, which is proved in Appendix F.

**Theorem 2** (Identification of the treatment confounding bridge function). *Under* Assumptions 2, 4, 5, 6, *and* 7, *for a* = 0, 1 *we have that*

$$\frac{{\left(1-\delta \right)}^{3}}{P(A=a|W,X,Y=0,S=1)}<E[q(a,Z,X)|W,A=a,X,Y=0,S=1]\;<\frac{1}{{\left(1-\delta \right)}^{3}P(A=a|W,X,Y=0,S=1)}$$

Thus, provided *δ* ≈ 0, Theorem 2 suggests that an approximation to the treatment confounding bridge function can be obtained by solving the following integral equation involving only observed data

(11) $$E\left[q^{*}(A,Z,X)|W,A=a,X,Y=0,S=1\right]=1/P(A=a|W,X,Y=0,S=1).$$

as long as a solution exists. Accordingly, hereafter suppose that the following assumption holds.

**Assumption 8** (Existence of a unique solution to (11)). There exists a unique square-integrable function q*(*A*, *Z*, *X*) *that satisfies* (11).

Heuristically, uniqueness of a solution to (11) requires that variation in *W* is sufficiently informative about variation in *Z*, in the sense that there is no source of variation in *W* that is not associated with a corresponding source of variation in *Z*. See Appendix G for further elaboration of completeness conditions and D’Haultfoeuille (2011) and Newey and Powell (2003) for related use of the assumption in the literature. Below we briefly illustrate Assumption 8 in the examples of Section 2.3.1.

**Example 1′**. *Suppose U and Z are both binary*, *and a binary NCO W is also observed*. *Let $p_{za.w}^{\prime }=P(Z=z,A=a|W=w,Y=0,S=1)$ for z*, *a*, *w* ∈ {0, 1}, *then solving* (11) *is equivalent to solving the system of linear equations*

$$p_{0a.0}^{\prime }q^{*}(a,0)+p_{1a.0}^{\prime }q^{*}(a,1)=1;\;p_{0a.1}^{\prime }q^{*}(a,0)+p_{1a.1}^{\prime }q^{*}(a,1)=1,$$

*giving* $q^{*}(a,z)=\left[p_{1a.1}^{\prime }-p_{1a.0}^{\prime }+\left(p_{0a.0}^{\prime }-p_{0a.1}^{\prime }-p_{1a.1}^{\prime }+p_{1a.0}^{\prime }\right)z\right]/\left(p_{0a.0}^{\prime }p_{1a.1}^{\prime }-p_{0a.1}^{\prime }p_{1a.0}^{\prime }\right)$. *Note that the probabilities* $p_{za.w}^{\prime }$ *can all be estimated from the study sample*.

We emphasize that the solution to Equation (11) is ultimately an approximation to the (non-identifiable) treatment confounding bridge function in the target population. The accuracy of this approximation relies on the extent to which the rare disease assumption holds in the target population of interest. We study the potential bias resulting from a departure of this key assumption in the Appendix I. We further observe that, under the null hypothesis of no vaccine effectiveness, or if *W* has no direct effects on *Y* or *S*, then the function q*(*A*, *Z*, *X*) equals the treatment confounding bridge exactly, even for a non-rare disease outcome, as stated in the corollary below. We prove Corollary 1 in Appendix F.

**Corollary 1**. *Under the Assumptions of* Theorem 1 *and* Assumption 8, *if there is no vaccine effect against infection*, *such that Y* ⫫ *A*|*U*, *X*, *then*

E[q(A,Z,X)∣W,A=a,X,Y=0,S=1]=1/P(A=a∣W,X,Y=0,S=1).

From Theorem 2, we immediately have the following corollary which provides a basis for estimation of q*(*A*, *Z*, *X*) from the observed TND sample.

**Corollary 2**. *Under the conditions listed in* Theorem 2, *for any function m*(*W*, *A*, *X*), the solution q*(*A*, *Z*, *X*) *to* Equation (11) *also solves the population moment equation*

(12) $$E\left[m(W,A,X)q^{*}(A,Z,X)-m(W,1,X)-m(W,0,A)|Y=0,S=1\right]=0.$$

We prove Corollary 2 in Appendix H. In practical situations where a parametric model q*(*A*, *Z*, *X*; *τ*) for the treatment confounding bridge function might be appropriate, where *τ* is an unknown finite dimensional parameter indexing the model, Corollary 2 suggests one can estimate *τ* by solving the estimating equation

(13) $$\frac{1}{n}\sum_{i=1}^{n}\left(1-Y_{i}\right)[m(W,A,X)q(A,Z,X;\tau )-m(W,1,X)-m(W,0,X)]=0,$$

where *m*(*W*, *A*, *X*) is a user-specified function whose dimension is no smaller than *τ*’s.

**Example 1″**. *If Z and W are both binary*, *rather than solving the system of equations implied by* (11), *one can instead specify a saturated model for the treatment confounding bridge function*:

(14) $$q^{*}(A,Z;\tau )=\tau_{0}+\tau_{1}Z+\tau_{2}A+\tau_{3}ZA$$

*and estimate τ* = (*τ*_0_, *τ*_1_, *τ*_2_, *τ*_3_)^*T*^ *by solving* (13) *with m*(*W*, *A*) = (1, *W*, *A*, *WA*)^*T*^. *Extension to Z and X with multiple categories is straightforward*.

**Example 2**′. *In case of continuous* (*U*, *X*, *Z*), *result* (8) *suggests the model*

(15) $$q^{*}(A,Z,X;\tau )=1+\text{exp}\left[{\left(-1\right)}^{A}\left(\tau_{0}+\tau_{1}A+\tau_{2}Z+\tau_{3}X\right)\right].$$

*If a univariate NCO W is available*, *we may solve* (13) *with m*(*W*, *A*, *X*) = (1, *W*, *A*, *X*)^*T*^.

### 2.4 Estimation and Inference

In the previous sections, we have defined the causal parameter of interest *β*_0_ as stratum-specific log risk ratio, introduced the treatment confounding bridge function as a key ingredient to identification of *β*_0_, and presented a strategy to estimate the treatment confounding bridge function leveraging an available NCO. We summarize the steps of our estimation framework in Algorithm 1 and present the large-sample properties of the resulting estimator ($\hat{\beta }$, $\hat{\tau }$) in Theorem 3.


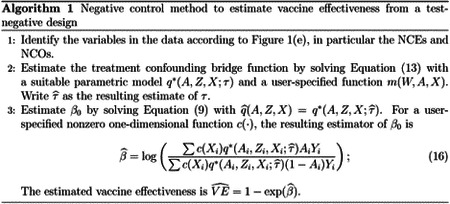


**Theorem 3** (Inference based on ($\hat{\beta }$, $\hat{\tau }$)). *Under* Assumptions 1–8 *and suitable regularity conditions provided in* Appendix J, *the estimator* ($\hat{\beta }$, $\hat{\tau }$) *in Algorithm 1*, *or equivalently*, *the solution to the estimating equation $\frac{1}{n}\sum_{i=1}^{n}G_{i}(\beta ,\tau )=0$ is regular and asymptotically linear with the i-th influence function IF*_*i*_(*β*, *τ*) = −Ω(*β*, *τ*)^+^*G*_*i*_(*β*, *τ*), *where* Ω(*β*, *τ*)^+^ *denotes the Moore-Penrose inverse of* Ω(*β*, *τ*),

$$G_{i}(\beta ,\tau )=\left(\begin{array}{c} {\left(-1\right)}^{1-A_{i}}c\left(X_{i}\right)q^{*}\left(A_{i},Z_{i},X_{i};\tau \right)Y_{i}\text{exp}\left(-\beta A_{i}\right)\\ \left(1-Y_{i}\right)\left[m\left(W_{i},A_{i},X_{i}\right)q^{*}\left(A_{i},Z_{i},X_{i};\tau \right)-m\left(W_{i},1,X_{i}\right)-m\left(W_{i},0,X_{i}\right)\right] \end{array}\right)$$

*and* Ω(*β*, *τ*) = (*E* [∂*G*_*i*_(*β*, *τ*)/∂*β*^*T*^], E [∂*G*_*i*_(*β*, *τ*)/∂*τ*^*T*^]).

The proof follows from standard estimating equation theory (See Van der Vaart (2000) Theorem 5.21). An immediate consequence of Theorem 3 is that we may estimate the variance-covariance matrix of ($\hat{\beta }$, $\hat{\tau }$) with

(17) $$\hat{\Omega }{\left(\hat{\beta },\hat{\tau }\right)}^{+}\hat{Var}\left(G_{i}(\hat{\beta },\hat{\tau })\right){\left[\hat{\Omega }{\left(\hat{\beta },\hat{\tau }\right)}^{+}\right]}^{T}/n,$$

where $\hat{\Omega }(\beta ,\tau )=\left(\hat{E}\left[\partial G_{i}(\beta ,\tau )/{\partial \beta^{T}|}_{\beta =\hat{\beta },\tau =\hat{\tau }}\right],\hat{E}\left[\partial G_{i}(\beta ,\tau )/{\partial \tau^{T}|}_{\beta =\hat{\beta },\tau =\hat{\tau }}\right]\right)$. Here $\hat{E}$ and $\hat{Var}$ denote the expectation and variance with respect to the empirical distribution, respectively. A two-sided *α*-level Wald-type confidence interval of VE can then be obtained as $\left(1-\text{exp}\left(\hat{\beta }-z_{1-\alpha /2}\sqrt{{\hat{\Sigma }}_{n,1,1}}\right),1-\text{exp}\left(\hat{\beta }+z_{1-\alpha /2}\sqrt{{\hat{\Sigma }}_{n,1,1}}\right)\right)$, where ${\hat{\Sigma }}_{n,1,1}$ is the (1, 1)-th entry of ${\hat{\Sigma }}_{n}$ and *z*_1−*α*/2_ is the (1 − *α*/2)-th quantile of a standard normal distribution.

The estimator $\hat{\beta }$ and the above confidence interval are constructed under the assumption that the disease is rare in the target population; for non-rare diseases, $\hat{\beta }$ is in general going to be biased and the confidence interval may not be well-calibrated. However, by Corollary 1, under the null hypothesis of no vaccine effects, the estimated q*(*A*, *Z*, *X*) converges to the true treatment confounding bridge function and $\hat{\beta }$ is consistent for *β*_0_ = 0. This implies that while our methods are approximately asymptotically unbiased for rare infections, they provide a valid test of no vaccine effect even if the infection is not rare.

### 2.5 Accounting for effect modification by measured confounders

So far we have operated under Assumption 3 that VE is constant across levels of (*U*, *X*). As we now show, this assumption can be relaxed to allow for potential effect modification with respect to *X* without compromising identification. This extension is particularly important because empirical evidence has indeed suggested that flu vaccine effectiveness may vary across sex and age groups (Chambers et al., 2018); and similar effect heterogeneity is of key interest in case of COVID-19 (Fernández Villalobos et al., 2021).

Instead of Assumption 3, we consider a less stringent assumption:

**Assumption 9** (No effect modification by unmeasured confounders).

(18) $$P(Y=1|A=a,U,X)=\text{exp}\left(\beta_{0}(X)a\right)g(U,X)$$

*where β*_0_(*x*) *are g*(*u*, *x*) *are unknown functions of x and u*, *x respectively*.

Under condition 1, Assumption 9 further implies that *β*_0_(*x*) = *E*[*Y* (1)|*X* = *x*]/*E*[*Y* (0)|*X* = *x*], i.e. the conditional causal RR as a function of *x*. Similar to Theorem 1, we have:

**Theorem 4**. *Under* Assumptions 1, 2, 4, 5 *and* 9, *for an arbitrary function c*(·) *we have that*

$$E\left[V_{3}\left(A,Y,Z,X;\beta_{0}\right)|S=1\right]=0,$$

*where V*_3_(*A*, *Y*, *Z*, *X*; *β*_0_) = (−1)^1−*A*^*c*(*X*)*q*(*A*, *Z*, *X*)exp(−*β*_0_(*X*)*A*).

The proof of Theorem 4 is identical to that of Theorem 1 with *β*_0_*A* replaced with *β*_0_(*X*)*A*. Identification and estimation of the treatment confounding bridge function are also essentially identical to that of Corollary 2. Therefore, it is straightforward to extend Algorithm 1 to allow effect modification by measured confounders. We describe the algorithm and the large sample properties of the resulting estimator in Appendix K.

### 2.6 Estimating VE under treatment-induced selection into TND sample

Thus far, unbiasedness of the estimating function *V*_0_ has crucially relied on Assumption 2 that *A* does not have a direct effect on *S*. In some settings, the assumption may be violated if an infected person who is vaccinated is on average more likely to present to the ER than an unvaccinated infected person with similar symptoms, so that treatment or vaccination-induced selection into the TND sample is said to be present. In such settings, both estimators $\hat{\beta }$ and $\hat{\beta }\left(X\right)$ produced by Algorithms 1 and 2 may be severely biased because Assumption 2 may no longer be valid. Crucially, we note that this form of selection bias can be present even in context of a randomized trial in which vaccination/treatment is assigned completely at random, if the outcome is ascertained using a TND, for example in the cluster-randomized test-negative design studies of community-level dengue intervention effectiveness Anders et al. (2018), Dufault and Jewell (2020), Jewell et al. (2019), and Wang et al. (2022). In this Section, we provide sufficient conditions for identification under treatment-induced selection. In this vein, consider the following assumptions:

**Assumption 2′**. *P*(*S* = 1|*A* = *a*, *Y* = 1, *U*, *X*)/*P*(*S* = 1|*A* = *a*, *Y* = 0, *U*, *X*) = exp(*h*(*U*, *X*)) *for a* = 0, 1.

That is, the risk ratio association between infection status and selection into the TND sample is independent of vaccination status. Furthermore,

**Assumption 3′**. *(No effect modification by confounders on the OR scale)*.

$$\frac{P(Y=1|A=1,U,X)/P(Y=0|A=1,U,X)}{P(Y=1|A=0,U,X)/P(Y=0|A=0,U,X)}=\text{exp}\left(\beta_{0}^{\prime }\right).$$

Recall that Assumption 3 posited a constant vaccination causal effect on the RR scale across levels of (*U*, *X*), while Assumption 3′ posits that the corresponding causal effect on the odds ratio scale is constant across levels of (*U*, *X*). In case of a rare infection in the target population, the OR and RR are approximately equal, in which case VE is well approximated by 1 − *OR*.

Furthermore, identification relies on the following modified definition of a treatment confounding bridge function:

**Assumption 5′**. *There exists a treatment confounding bridge function $\tilde{q}$ such that for a* = 0, 1,

(19) $$E[\tilde{q}(a,Z,X)|A=a,U,X]=1/P(A=a|U,X,Y=0,S=1)\;\text{almost}\;\text{surely. }$$

Note that if the infection is rare in the target population in the sense of Assumption 7, then the treatment confounding bridge function defined in Assumption 5 in Section 2.3.1 satisfies (19) approximately.

We now introduce the identification of the OR with the following theorem:

**Theorem 1′**. *Under* Assumptions 1, 2′, 3′, 4 *and* 5′, *we have*

$$E\left[{\tilde{V}}_{1}\left(A,Y,Z,X;\beta_{0}^{\prime }\right)|U,X,S=1\right]=0$$

*where ${\tilde{V}}_{1}(A,Y,Z,X;\beta )$ is the same as V*_1_ *defined in* Theorem 1 *except with* $\tilde{q}$ *replacing q*.

Importantly, the theorem establishes that the estimating function *V*_1_ previously developed in the paper can under certain conditions, remain unbiased for the odds ratio association of vaccination with testing positive for the infection, even in the presence of treatment-induced selection into the TND sample. We leave the proof of Theorem 1′ to Appendix L.

Estimation of the treatment confounding bridge function $\tilde{q}$ requires a negative control outcome that satisfies:

**Assumption 6′**. *(NCO Independence Conditions) W* ⫫ (*A*, *Z*, *S*)|*U*, *X*, *Y*.

In addition to Assumptions 6, this last assumption requires that neither *Y* nor *S* is a causal effect of *W*. Figure 1(f) illustrates a DAG that satisfies our assumptions regarding (*Z*, *W*). As can be verified in the graph, Assumption 6′ is needed to ensure that collider stratification bias induced by the path *A* → [*S* = 1] ← *W* upon conditioning on *S* = 1 is no longer present. Identification of the function $\tilde{q}$ is given below:

**Theorem 2′**. *Under* assumptions 4, 5′ *and* 6′, *for a* = 0, 1 *we have that*

$$E[\tilde{q}(a,Z,X)|A=a,W,X,Y=0,S=1]=1/P(A=a|W,X,Y=0,S=1)$$

We prove Theorem 2′ in Appendix M. As a result of Theorem 2′, the parameters in the treatment confounding bridge function can be estimated by solving moment equation (13).

In summary, the above discussion suggests that one can continue to use Algorithm 1 to estimate VE in presence of treatment induced selection bias, albeit on the OR scale and under a modified set of negative control conditions. Algorithm 2 can similarly be justified under treatment-induced selection with assumptions in this section, except that $\beta_{0}^{\prime }$ in Assumption 3′ is replaced by the conditional log RR $\beta_{0}^{\prime }(X)$.

As a side note, Assumption 2′ automatically holds under Assumption 2, and hence the above results in this section also apply to the setting in previous sections that is illustrated in Figure 1(e). We present this statement in the following corollary.

**Corollary 3**. *Under* Assumptions 1, 2, 3′, 4 *and* 5′, *we have*

$$E\left[{\tilde{V}}_{1}\left(A,Y,Z,X;\beta_{0}^{\prime }\right)|U,XS=1\right]=0.$$

With Assumption 2, the treatment confounding bridge function $\tilde{q}$ can be estimated by solving the moment equation (13) either under under Assumption 6′ and 8, or Assumptions 6, 7 and 8 as an approximation under the rare disease assumption. Corollary 3 leads to an interesting observation: under the treatment-independent sampling (Assumption 2), the estimator $\hat{\beta }$ from Algorithm 1 can be viewed as either log RR or log OR, depending on the set of assumptions made.

## 3 Simulation Study

To assess the empirical performance of our proposed method, we consider two settings with different types of confounding and negative control variables, and perform corresponding simulation studies.

In the first setting, we consider no measured confounder, a binary unmeasured confounder *U*, a binary NCE *Z* and a binary NCO *W*. To trigger selection among subjects with *Y* = 0, we let *D* be a binary indicator of the presence of other flu like illnesses. The treatment confounding bridge function is thus given by (7). We assume the distribution of *Y* is Bernoulli with a log-linear risk model: *Y* |*A*, *U* ~ *Bernoulli*(exp(*η*_0*Y*_ + *β*_0_*A* + *η*_*UY*_ *U*)). We consider values of *β*_0_ to be −1.609, −0.693, −0.357 or 0, corresponding to a risk ratio of 0.2, 0.5, 0.7 or 1. We assume the selection *S* only equals one with nonzero probability if at least one of *Y*, *W* and *D* equals one, and is independent of *A* and *Z* conditional on other variables. The resulting prevalence of *Y* in the target population is 0.75% among the unvaccinated individuals and 0.55%, 0.65%, 0.72% or 0.75% among the vaccinated individuals, corresponding to four values of *β*_0_. Next, we consider a setting where *X*, *U*, *Z* and *W* are all univariate continuous variables. We generate the infection outcome using a log-linear model

$$Y|A,U,X\sim \;\text{Bernoulli }\left(\text{exp}\left(\mu_{0Y}+\beta_{0}A+\mu_{UY}U+\mu_{XY}X+\mu_{UXY}UX\right)\right).$$

We generate *A* and *Z* following Example 2 in Section 2.3. As such the treatment confounding bridge function is given by Equation (8). The probability of *S* = 1 is 1 only if at least one of *Y* and *D* is nonzero. The resulting prevalence of *Y* in the target population is 0.34% among the unvaccinated individuals and 0.24%, 0.28%, 0.31% or 0.34% among the vaccinated individuals, corresponding to four values of *β*_0_. Appendix N and O give more details on the data-generating mechanism for the two settings.

In each scenario, we simulate a target population of size *N* = 7, 000, 000 and implement 1, 000 simulation iterations. For both settings, we evaluate the performance of three estimators for *β*_0_:

- NC estimator: our proposed estimator given by Algorithm 1. In the first setting, we use a saturated parametric model (14) for the treatment confounding bridge function in the first setting, with *m*(*W*, *A*) = (1, *W*, *A*, *WA*)^*T*^ ; in the second setting, we use model (15) and *m*(*W*, *A*, *X*) = (1, *W*, *A*, *X*)^*T*^. We set *c*(*X*) = 1 in both settings.
- NC-Oracle estimator: the estimated treatment confounding bridge function in Algorithm 1 is only an approximation under Assumption 7, whose bias may affect the estimation for *β*_0_, as derived in Appendix I. We therefore include NC-Oracle estimator that uses the true treatment confounding bridge function *q*(*A*, *Z*, *X*). Appendices E include derivation of the true treatment confounding bridge function under the continuous (*X*, *U*, *Z*, *W*) setting.
- Logistic regression: we also consider a logistic regression model of *Y* on *A* (and *X* in the second setting), overlooking the unmeasured confounders *U*. This is a common choice for covariate adjusted analyses of test-negative designs but ignores biases caused by *U* (Bond, Sullivan, and Cowling, 2016). We comment in Appendix P that the estimator is appropriate in the absence of unmeasured confounders except for potential model misspecification.

We note that the target parameter *β*_0_ for NC estimator and NC-Oracle estimator is log causal RR, while logistic regression gives log causal OR. However, the two parameters are approximately equal under Assumption 7 where the infection risk is low in the target population.

**Figure 2:**
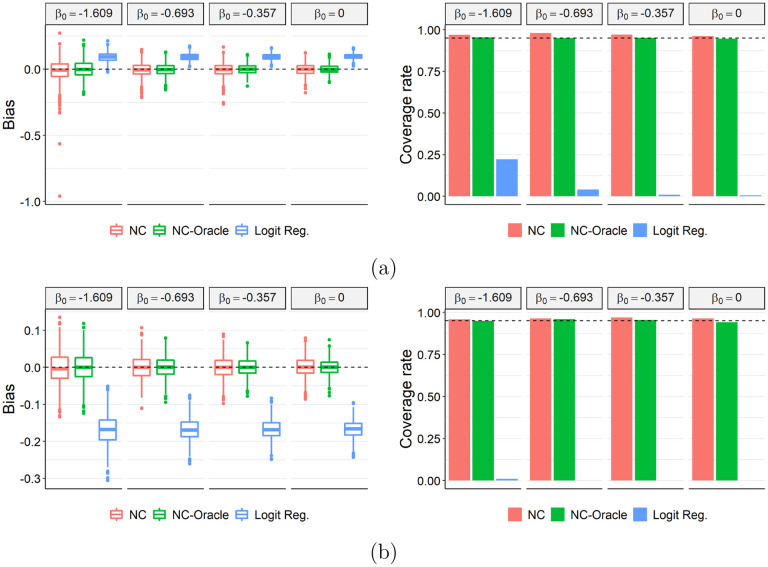
Bias (left) and coverage rates of 95% confidence interval (right) for the oracle estimator (NC-Oracle), GMM estimator (NC-GMM) and logistic regression (Logit Reg.) with a (a) binary or (b) continuous unmeasured confounder.

Figure 2 shows the bias of three estimators considered and the coverage of their 95% confidence intervals. In both settings, both NC and NC-Oracle are essentially unbiased whereas logistic regression gives a biased estimate in all scenarios. NC-Oracle exhibits slightly higher precision than NC, which implies that estimating the treatment confounding bridge function in the TND is only slightly more variable. The 95% confidence intervals for NC and NC-Oracle both achieve nominal coverage, whereas logistic regression-based confidence intervals under-cover severely. We repeated the simulation under a non-rare disease setting in Appendix Q. In such scenarios, while NC-Oracle estimator is still unbiased with calibrated 95% confidence intervals, the NC estimator is biased in general except when *β*_0_ = 0. We conclude that the proposed NC estimator is unbiased of the log causal RR either under a rare disease setting or under a non-rare disease setting with no vaccine effect.

## 4 Application

We applied our proposed method to a TND study of COVID-19 VE of two-dose Moderna vaccine (mRNA-1273), two-dose Pfizer-BioNTech vaccine (BNT162b2), and single-dose Johnson & Johnson’s Janssen vaccine (Ad26.COV2.S) against COVID-19 infection nested in the University of Michigan Health System. The selected study sample includes patients who interacted with the University of Michigan Health System and experienced COVID-19 symptoms, had suspected exposure to COVID-19 virus, or sought to screen for COVID-19 infection, between April 5, 2021 and December 7, 2021. In addition, the selected test-positive subjects had at least one positive lab tests for COVID-19 infection after April 5. Vaccination history was obtained through electronic health records. A study subject was considered fully vaccinated if they received at least one dose of Johnson & Johnson’s Janssen vaccine or at least two doses of Moderna or Pfizer-BioNTech vaccine. If a subject tested positive before or within 14 days after their first dose of Janssen vaccine or within 14 days after their second dose of Moderna or Pfizer-BioNTech vaccine, they were considered unvaccinated (Moline et al., 2021).

We took immunization visits before December 2020 as NCE since COVID-19 vaccines were not available before December 2020 and immunization before was unlikely to affect the risk of COVID-19 infection; nor that of the selected NCOs we describe next. For NCO, we selected a binary indicator of having at least one of the following “negative control outcome” conditions after April 5, 2021: arm/leg cellulitis, eye/ear disorder, gastro-esophageal disease, atopic dermatitis, and injuries. Such candidate NCE and NCO are likely to satisfy the requisite conditional independence conditions for them to be valid negative control variables and to be related to a patient’s latent HSB. We adjusted for age groups (<18, between 18 and 60, or ≥ 60), gender, race (white or non-white), Charlson comorbidity score ≥ 3, and the calendar month of a test-positive subject’s first positive COVID test or a test-negative subject’s last COVID test. Table S3 in Appendix R summarizes the distribution of negative control variables, demographic variables and COVID-19 infection among vaccinated and unvaccinated subjects.

Because NCE is expected not to be associated with either the outcome or NCE in a fully adjusted analysis unless there is unmeasured confounding, we first fit regression models to detect presence of residual confounding bias. Conditioning on the baseline covariates, in both vaccinated and unvaccinated groups, NCE is significantly associated with COVID-19 infection (*p* < 0.001) and NCO (*p* < 0.001) in corresponding adjusted logistic regression models, suggesting the presence of hidden biases (See Appendix R Table S4, S5).

We implemented Algorithm 1 to estimate VE. We specified a linear model for the treatment confounding bridge function with an interaction term between COVID-19 vaccination and the NCE, and set the function *m* to include one (for an intercept term), COVID-19 vaccination, the NCO, and baseline covariates, as well as all two-way interactions. For comparison, we also implemented a logistic regression model, which gives an unbiased estimate of causal odds ratio under the no unmeasured confounding assumption, adjusting for gender and age groups. In this case, The VE can be approximated by one minus the odds ratio of COVID-19 infection against vaccination, as COVID-19 infection rate is known to be low across strata in the target population.

The double negative control Algorithm 1 estimated the VE against lab-confirmed COVID-19 infection were 71.6% (95% CI: 68.9%, 74.0%) for the two-dose Pfizer-BioNTech vaccine, 86.8% (95% CI: 84.7%, 88.6%) for the two-dose Moderna vaccine, and 66.0% (95% CI: 54.5%, 74.6%) for the one-dose Johnson & Johnson’s Janssen vaccine. The logistic regression estimates for the same VE were 67.0% (95% CI: 64.5%, 69.4%) for the Pfizer-BioNTech vaccine, 75.1% (95% CI, 72.5%, 77.5%) for the Moderna vaccine, and 56.0% (95%: 47.9%, 62.9%) for the Janssen vaccine. There is significant evidence of hidden bias as summarized in Tables S4 and S5. The VE estimates with our double NC method are notably higher than those given by the standard logistic regression for all three regimens.

Recent observational studies estimated real-world mRNA COVID-19 vaccine (Pfizer-BioNTech and Moderna) effectiveness ranging between 80% and 98% against lab confirmed SARS-COV-2 infection of different variants (**bruxvoort2021effectiveness**; **israel2021elapsed**; Dagan et al., 2021). Our NC approach provided VE estimates that are close to the previous studies. We hypothesize that the standard logistic regression underestimates the VE by overlooking the confounding bias due to HSB and related factors, which our proposed double NC approach appears to reduce to some extent.

## 5 Discussion

In this article, we have introduced a statistical method for estimating vaccine effectiveness in a test-negative design. The approach leverages negative control variables to account for hidden bias due to residual confounding and/or selection mechanism into the TND sample. Negative control variables abound in practice, such as vaccination history which is routinely collected in insurance claims and electronic health records. Hence the proposed method may be particularly useful in such real world settings to obtain improved estimates of vaccine effectiveness.

The TND is a challenging setting in causal inference where selection bias and unmeasured confounding co-exist, selection is outcome-dependent, and unmeasured confounders also impact selection. As a result, the causal effect of interest is in general not identified from such studies (Cai and Kuroki, 2012). Nevertheless, we establish that progress can be made under a semiparametric multiplicative model, provided the outcome is rare in the target population, and double negative control variables are available. To this end, this article showcases the potential power of negative control methods and proximal causal inference in epidemiologic research (Shi, Miao, and Tchetgen Tchetgen, 2020; Tchetgen Tchetgen et al., 2020).

We focused on the outpatient TND, where recruitment is restricted to subjects who seek care voluntarily. TNDs have also been applied to inpatient settings for studying VE against, for example, flu hospitalization (Feng, Cowling, and Sullivan, 2016; Foppa et al., 2016). In inpatient TNDs, differential access to healthcare and underlying health characteristics between vaccinated and unvaccinated subjects are likely the main causes of confounding bias (Feng, Cowling, and Sullivan, 2016). Our methods are still applicable in such settings, but negative control variables should be selected to be relevant to the source of unmeasured confounding mechanism. For example, previous vaccination and hospitalization outside the flu season or hospitalization due to other flu-like illnesses are viable candidate NCE and NCO, respectively (Jackson et al., 2006).

Our approach is suitable for post-market TND studies where real-world vaccine effectiveness is of interest and vaccination history is obtained retrospectively, possibly through electronic health records. For vaccine efficacy in a controlled trial setting, Wang et al. (2022) recently developed estimation and inference of RR in cluster-randomized TND, aiming to correct for bias due to differential HSB induced by the intervention being unblinded. Because of randomization, they considered HSB as a post-treatment variable and proposed a log-contrast estimator which corrects for selection bias by leveraging a valid test-negative outcome, under an assumption that either (i) the vaccine does not have a causal effect in the population, and the causal impact of vaccination on selection is equal for test-positive and -negative subsamples; or (ii) among care seekers, the incidence of test-negative outcomes does not differ between vaccinated and unvaccinated, and the intervention effect among care seekers is generalizable to the whole population. We note that even under randomization, identification conditions given in Section 2.6 are neither stronger nor weaker than those of Wang et al. (2022) described above, as neither set of assumptions appear to imply the other. An important advantage of our proposed methods is that they can be used to account for selection bias in a TND study irrespective of randomization.

Our methods target RR as a measure of VE instead of the more common OR (Jackson et al., 2006; Sullivan, Tchetgen Tchetgen, and Cowling, 2016). These two measures are approximately equal for rare infections. Schnitzer (2022) recently considered estimation of a marginal causal RR in the TND sample and justified the use of an inverse probability of treatment weighted (IPTW) estimator in a setting in which an unmeasured common cause of infection and selection into the TND sample does not cause vaccination (and thus there is no unmeasured confounding). Instead, our methods allow for an unmeasured common cause of vaccination, infection and selection into the TND sample; however in order to estimate a causal RR, we invoke both, an assumptions of no effect modification by an unmeasured confounder, and a rare-disease condition. As we establish, the latter assumption is not needed if there is no vaccine effect against infection outcome. In Section 2.6, we establish that under a homogeneous OR vaccine effect measure condition, and an alternative definition of the treatment bridge function, our methods can identify a causal effect of the vaccine on the odds ratio scale without invoking the rare disease condition.

Throughout the article, we have assumed diagnostic tests are accurate and individuals who seek care are sparsely distributed, such that the vaccination of a given subject in the TND sample does not protect another study subject from infection, , i.e. there is no interference in the TND sample, a common assumption in TND literature. This assumption may be violated if members of the same households present in the ER in which case block interference must be accounted using results from interference literature (Hudgens and Halloran, 2008; Tchetgen Tchetgen and VanderWeele, 2012). Sensitivity analysis may be considered to evaluate how violation of these assumptions can potentially bias inferences about VE.

## Supplementary Material

1
