# Double Negative Control Inference in Test-Negative Design Studies of Vaccine Effectiveness

**Published:** 2022-03-23

**Authors:** Kendrick Qijun Li, Xu Shi, Wang Miao, Eric Tchetgen Tchetgen

**Affiliations:** Department of Biostatistics, University of Michigan; Department of Biostatistics, University of Michigan; Department of Probability and Statistics, Peking University; Department of Statistics and Data Science, The Wharton School, University of Pennsylvania

**Keywords:** Causal inference, proximal causal inference, selection bias, unmeasured confounding

## Abstract

The test-negative design (TND) has become a standard approach to evaluate vaccine effectiveness against the risk of acquiring infectious diseases in real-world settings, such as Influenza, Rotavirus, Dengue fever, and more recently COVID-19. In a TND study, individuals who experience symptoms and seek care are recruited and tested for the infectious disease which defines cases and controls. Despite TND’s potential to reduce unobserved differences in healthcare seeking behavior (HSB) between vaccinated and unvaccinated subjects, it remains subject to various potential biases. First, residual confounding may remain due to unobserved HSB, occupation as healthcare worker, or previous infection history. Second, because selection into the TND sample is a common consequence of infection and HSB, collider stratification bias may exist when conditioning the analysis on tested samples, which further induces confounding by latent HSB. In this paper, we present a novel approach to identify and estimate vaccine effectiveness in the target population by carefully leveraging a pair of negative control exposure and outcome variables to account for potential hidden bias in TND studies. We illustrate our proposed method with extensive simulations and an application to study COVID-19 vaccine effectiveness using data from the University of Michigan Health System.

## 1 Introduction

### 1.1 Text-negative design studies of vaccine effectiveness

The test-negative design (TND) has become a standard approach to evaluate real-world vaccine effectiveness (VE) against the risk of acquiring infectious diseases (Sullivan et al. 2014, Chua et al. 2020). In an outpatient Influenza VE TND study, for example, symptomatic individuals seeking care and meeting eligibility criteria are enrolled and their Influenza virus infection status is subsequently confirmed via a laboratory test. VE against flu infection is then measured by comparing the prevalence of vaccination between the test-positive “cases” and test-negative “controls” (Jackson & Nelson 2013, Jackson et al. 2017). Besides Influenza, the TND and its variants have also been used to study VE against pneumococcal disease (Broome et al. 1980), dengue (Anders et al. 2018), rotavirus (Boom et al. 2010), and other infectious diseases. Recently, the TND has increasingly been used in post-licensure evaluation of COVID-19 VE (Patel et al. 2020, Dean et al. 2021, Hitchings et al. 2021, Thompson et al. 2021, Dagan et al. 2021, Olson et al. 2022).

Test-negative designs are believed to reduce unmeasured confounding bias due to healthcare-seeking behavior (HSB), whereby care seekers are more likely to be vaccinated, have healthier behaviors that reduce the risk of infection, and get tested when ill (Jackson et al. 2006, Shrank et al. 2011). By restricting analysis to care seekers who are tested for the infection in view (e.g. Influenza or COVID-19), the vaccinated and unvaccinated are more likely to share similar HSB and underlying health characteristics. Misclassification of infection status is also reduced because the analysis is restricted to tested individuals (Jackson & Nelson 2013).

**Figure 1:**
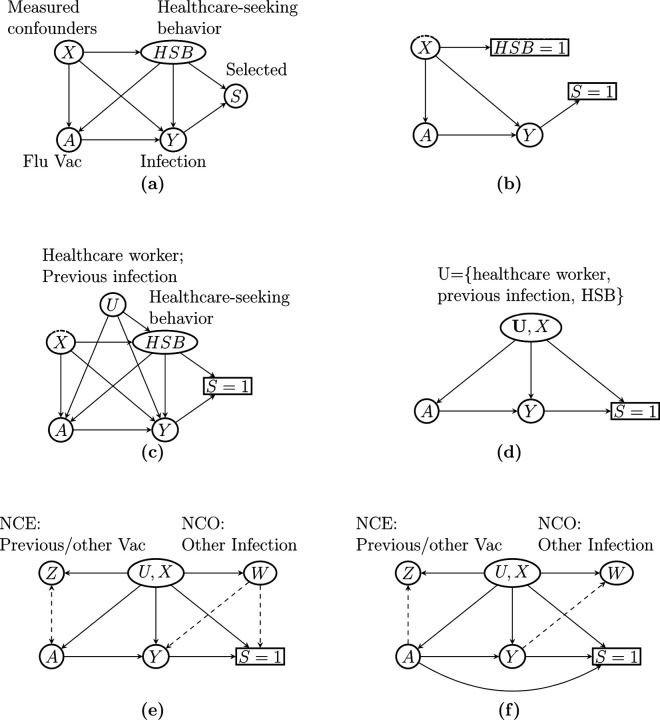
Causal relationships of variables in a test-negative design. Sullivan et al. (2016) used (a) to illustrate the causal relationship between variables in a test-negative design in the general population, and used (b) to illustrate the assumption implicit in the common approach to estimate VE from the study data that study subjects have identical healthcare-seeking behavior (HSB) (Sullivan et al. 2016). (c) shows that if *HSB* remains partially unobserved, then the backdoor paths *A* ← *HSB* → *Y* and *A* ← *HSB* → *S* = 1 ← *Y* indicate unmeasured confounding bias and selection bias, respectively. Other unmeasured confounders, such as occupation as a healthcare worker and previous infection, open additional backdoor paths between *A* and *Y* and result in additional confounding bias. (d) shows a simplified DAG from (c) that combines the unmeasured confounders into a single variable *U*. (e) illustrates our approach to estimate VE leveraging negative control exposure *Z* and outcome *W*. Dashed arrows indicate effects that are not required. (f) shows a scenario with the *A* → *S* arrow where the causal odds ratio can still be identified under additional assumptions.

Sullivan et al. (2016) used directed acyclic graphs (DAG) to illustrate the rationale behind TND in the context of evaluating VE against flu infection, as shown in Figures 1(a) and (b). We denote flu vaccination status by *A* and flu infection by *Y*, so that the arrow *A* → *Y* represents VE against flu infection. Selection into the TND study sample, denoted by *S*, is triggered by a subject experiencing flu-like symptoms, seeking care at clinics or hospitals, and getting tested for Influenza infection, hence the *Y* → *S* edge. Healthcare-seeking behavior, denoted by HSB, may affect *S*, *A*, and *Y* because subjects with certain healthcare-seeking proclivities may be more likely to seek care, take annual flu shots, and participate in healthy and preventive behaviors. The above variables may be subject to effects of other clinical or demographic factors, such as age, season, and high-risk conditions, included in Figure 1 as confounders *X*. The TND presumes that by restricting recruitment to care seekers, the study subjects essentially have identical HSB; in other words, conditioning the analysis on *S* = 1 is equivalent to conditioning on HSB= 1, which would then completely control for HSB (Figure 1(b)). Measured covariates *X* are further adjusted for by including these factors in a logistic regression model or by inverse probability weighting (Bond et al. 2016, Thompson et al. 2021).

However, the TND remains subject to potential hidden bias. First, it is unrealistic that all study subjects seeking care are lumped into a single category HSB= 1. It may be more realistic that HSB is not a deterministic function of *S* and remains a source of confounding bias even after conditioning on *S*. Furthermore, there might be other mismeasured or unmeasured confounders, denoted as *U*. For example, healthcare workers are at increased risk of flu infection due to higher exposure to flu patients and are more likely to seek care and receive vaccination due to health agency guidelines (Black et al. 2018). Prior flu infection history may also be a source of confounding if it alters the likelihood of vaccination and care-seeking, while also providing immunity against circulating strains (Sullivan et al. 2016, Krammer 2019). These potential sources of confounding, if not properly accounted for, can result in additional confounding bias, as illustrated in Figure 1(c). Finally, collider stratification bias is likely present due to conditioning on *S*, which is a common consequence of HSB, other risk factors (*X, U*), and Influenza infection *Y* (Lipsitch et al. 2016). That is, conditioning on *S* unblocks the backdoor path *A* ← (*X, U, HSB*) → *S* ← *Y*, which would in principle be blocked if study subjects had identical levels of HSB and other risk factors (Sullivan et al. 2016).

Accounting for these potential sources of bias is well known to be challenging, and potentially infeasible without additional assumptions or data. This can be seen in Figure 1(d), which is a simplified version of Figure 1(c) where the unmeasured confounders *U* include individuals’ occupation as a healthcare worker, previous flu infection, HSB, and so on. Figure 1(d) indicates that the unmeasured confounders *U* induce both confounding bias through the path *A* ← *U* → *Y* and collider stratification bias through the path *A* ← *U* → *S* ← *Y*. In presence of both unmeasured confounding and collider bias, causal bounds may be available (Gabriel et al. 2020) but likely too wide to be informative; causal identification in TND therefore remains to date an important open problem in the causal inference literature which we aim to resolve.

### 1.2 Negative control methods

In recent years, negative control variables have emerged as powerful tools to detect, reduce, and potentially correct for unmeasured confounding bias (Lipsitch et al. 2010, Miao, Geng & Tchetgen Tchetgen 2018, Shi, Miao & Tchetgen Tchetgen 2020). The framework requires that at least one of two types of negative control variables are available which are *a priori* known to satisfy certain conditions: a negative control exposure (NCE) known to have no direct effect on the primary outcome; or a negative control outcome (NCO), known not to be an effect of the primary exposure. Such negative control variables are only valid and therefore useful to address unmeasured confounding in a given setting to the extent that they are subject to the same source of confounding as the exposure-outcome relationship of primary interest. Thus, an observed association between a valid NCE and the primary outcome (conditional on the primary treatment and observed covariates) or one between a valid NCO and the primary exposure can indicate the presence of residual confounding bias. For example, in a cohort study to investigate flu VE against hospitalization and death among seniors, to detect the presence of confounding bias due to underlying health characteristics, Jackson et al. (2006) used hospitalization/death before and after the flu season as NCOs and found that the association between flu vaccination and hospitalization was virtually the same before and during the flu season, suggesting that the lower hospitalization rate observed among vaccinated seniors versus unvaccinated seniors was partially due to healthy-user bias.

Recently, new causal methods have been proposed to not only detect residual confounding when present, but also to potentially de-bias an observational estimate of a treatment causal effect in the presence of unmeasured confounders when both an NCE and an NCO are available, referred to as the double negative control (Miao, Geng & Tchetgen Tchetgen 2018, Shi, Miao, Nelson & Tchetgen Tchetgen 2020, Tchetgen Tchetgen et al. 2020). In this recent body of work, the double negative control design was extended in several important directions including settings in which confounding proxies routinely measured in well-designed observational studies may be used as negative control variables, a framework termed *proximal causal inference*; longitudinal settings where one is interested in the joint effects of time-varying exposures (Ying et al. 2021), potentially subject to both measured and unmeasured confounding by time-varying factors; and in settings where one aims to estimate direct and indirect effects in mediation analysis subject to unmeasured confounding or unmeasured mediators (Dukes et al. 2021, Ghassami, Shpitser & Tchetgen Tchetgen 2021). Other recent papers in this fast-growing literature include Qi et al. (2021), Liu & Tchetgen Tchetgen (2021), Egami & Tchetgen Tchetgen (2021), Kallus et al. (2021), Imbens et al. (2021), Deaner (2018), Ghassami, Ying, Shpitser, Tchetgen Tchetgen et al. (2021), Mastouri et al. (2021) and Ghassami et al. (2022). Notably, existing identification results in negative control and proximal causal inference literature have been restricted to i.i.d settings (Miao, Shi & Tchetgen Tchetgen 2018) and time series settings (Shi et al. 2021), and to date, to the best of our knowledge, outcome-dependent sampling settings such as TND have not been considered, particularly one where confounding and selection bias might co-exist.

### 1.3 Outline

The rest of the paper is organized as followed: we introduce notation and the identification under no unmeasured confounding nor selection bias in Sections 2.1. Next we develop our identification strategy and describe a new debiased estimator under a double negative control TND study in Sections 2.2–2.4, assuming no direct effect of vaccination on selection into the TND sample. In Section 2.5, we relax this assumption and introduce sufficient conditions under which VE remains identified. In Section 3, we demonstrate the performance of our method with simulations. In Section 4, the approach is further illustrated in an application to estimate COVID-19 VE against infection in a TND study nested within electronic health records from University of Michigan Health System. We conclude with a discussion in Section 5. We relegate all proofs, derivations, additional tables and figures, and detailed discussions to Section A–L of the Supplementary Materials.

## 2 Method

### 2.1 Preliminary: estimation under no unmeasured confounding and no selection bias

To fix ideas, we first review estimation assuming all confounders (*U, X*) are fully observed and the study sample is randomly drawn (rather than selected by testing) from a source population, referred to as the “target population”. That is, we observe data on (*A, Y, U, X*) which are independent and identically distributed in the target population. For each individual, we write *Y* (*a*) as the binary potential infection outcome had, possibly contrary to fact, the person’s vaccination status been *A* = *a*, *a* = 0, 1. Our goal is to provide identification and estimation strategies for the causal risk ratio (RR) defined as *RR* = *P*[*Y* (1) = 1]*/P*[*Y* (0) = 0]. Let *β*_0_ denote the log causal RR, i.e., *RR* = exp(*β*_0_). Following Hudgens & Halloran (2006), we define VE as one minus the causal RR: *V E* = 1 – exp(*β*_0_). Let *Q*(*A* = *a, U, X*) = 1*/P* (*A* = *a*|*U, X*) denote the inverse of the probability of vaccination status *A* = *a* given confounders. Under the standard assumptions of consistency (which involves the assumption of no interference: a subject’s potential outcome is not affected by the treatment of other subjects (Cole & Frangakis 2009)), ignorability (given *U, X*) and positivity (Hernán MA 2020), it is well known that, if *U* were observed, the mean potential outcome *P*[*Y* (*a*) = 1] can be identified by inverse probability of treatment weighting (IPTW):

(1) $$P[Y\left(a\right)=1]=E[I\left(A=a\right)Q(A=a,U,X)Y],$$

for *a* = 0, 1. Therefore, the log causal RR *β*_0_ satisfies the following equation

$$E\left[Q(A=1,U,X)AY\exp\left(-\beta_{0}\right)\right]-E[Q(A=0,U,X)(1-A)Y]=0.$$

Equivalently, we have

(2) $$E[V_{0}\left(A,Y,U,X;\beta_{0}\right)]=0$$

where *V*_0_(*A, Y, U, X*; *β*) = (−1)^1–*A*^*Q*(*A, U, X*)*Y* exp(−*βA*) is an unbiased estimating function for *β*_0_.

### 2.2 Tackling selection bias under a semiparametric risk model

Next, consider a TND study for which data (*A, Y, X, U*) is observed only for the tested individuals with *S* = 1. Because *S* is influenced by other factors such as infection, the estimating function *V*_0_(*A, Y, U, X*; *β*_0_) may not be unbiased with respect to the study sample; i.e. *E*[*V*_0_(*A, Y, U, X*; *β*_0_)|*U, X, S* = 1] ≠ 0 without another assumption about the selection process into the TND sample.

For a TND sample of size *n*, we denote the *i*-th study subject’s variables as (*A*_*i*_*, Y*_*i*_*, U*_*i*_*, X*_*i*_), *i* = 1*, …, n*. For generalizability, we make the key assumption that vaccination *A* is unrelated to selection *S* other than through a subject’s infection status *Y* and confounders (*U, X*).

**Assumption 1** (Treatment-independent sampling). S⫫A|Y,U,X .

In a TND study, this assumption requires that an individual’s decision to seek care and get tested only depends on the presence of symptoms and his/her underlying behavioral or socioeconomic characteristics, including HSB (contained in (*U, X*)), and therefore vaccination status does not directly affect selection. The DAGs in Figure 1(a)–(e) in fact encode this conditional independence condition. We relax this assumption in Section 2.5. Outcome-dependent sampling distinguishes our work from standard proximal causal inference, which has exclusively assumed the availability of a random sample of subjects from the target population (Miao, Geng & Tchetgen Tchetgen 2018, Cui et al. 2020, Tchetgen Tchetgen et al. 2020). We further consider the following effect homogeneity condition.

**Assumption 2** (No effect modification). *For a* = 0, 1,

(3) $$P(Y=1|A=a,U,X)=\exp\left(\beta_{0}a\right)g(U,X)$$

*where g*(*U, X*) = *P*(*Y* = 1 | *A* = 0*, U, X*) *is an unknown function only restricted by* 0 ≤ *P* (*Y* = 1|*A, U, X*) ≤ 1.

Assumption 2 formally defines a semiparametric multiplicative risk model which posits that vaccine effectiveness, measured on the RR scale, is constant across (*U, X*) strata in the target population. In other words, VE is not modified by *U, X*. This assumption is stronger than necessary for our methods but simplifies the exposition. In Section H of the Supplementary Materials, we relax the assumption to allow for effect modification by measured confounders *X*. Infection risk for control subjects *P*(*Y* = 1|*A* = 0*, U, X*) = *g*(*U, X*) is the nonparametric component of the model which is left unspecified.

Under Assumption 2, one can verify that exp(*β*_0_) = *P*[*Y* (1) = 1]*/P*[*Y* (0) = 1], which is the marginal causal RR. Therefore, the estimating equation (2) implies that it is possible to identify *β*_0_ even though the potential outcome means *P*[*Y* (0) = 1] and *P*[*Y* (1) = 1] cannot be identified due to selection bias. The following proposition indicates that the same is true when the data are subject to selection bias of a certain structure.

**Proposition 1.** *Under Assumptions 1 and 2, the parameter β*_0_ *satisfies*

(4) $$E\left[V_{0}\left(A,Y,U,X;\beta_{0}\right)|U,X,S=1\right]=0.$$

From Proposition 1, the IPTW estimating function *V*_0_ derived from the target population remains unbiased in the TND sample. In principle, one could estimate *β*_0_ with $\hat{\beta }$, the solution to

(5) $$\frac{1}{n}\sum_{i=1}^{n}{\left(-1\right)}^{1-A_{i}}c\left(X_{i}\right)\hat{Q}\left(A_{i},U_{i},X_{i}\right)Y_{i}\exp\left(-\hat{\beta }A_{i}\right)=0,$$

where *c*(·) is a user specified function, and $\hat{Q}\left(A_{i},U_{i},X_{i}\right)=1/\hat{P}\left(A=A_{i}|U_{i},X_{i}\right)$ is the estimated probability of having vaccination status *A* = *A*_*i*_ given confounders (*U*_*i*_*, X*_*i*_). Letting *c*(*X*_*i*_) = 1, the resulting estimator

(6) $${\hat{\beta }}_{0}=\log\left\{\left[\sum_{i=1}^{n}\hat{Q}\left(A_{i},U_{i},X_{i}\right)A_{i}Y_{i}\right]/\left[\sum_{i=1}^{n}\hat{Q}\left(A_{i},U_{i},X_{i}\right)\left(1-A_{i}\right)Y_{i}\right]\right\}$$

is essentially the IPTW estimator of marginal RR in Schnitzer (2022) assuming (*U*_*i*_*, X*_*i*_)’s are all observed.

However, *Q*(*A, U, X*) cannot be estimated because *U* is unobserved. Furthermore, even if *U* were observed, $\hat{Q}\left(A_{i},U_{i},X_{i}\right)$ may not be identified from the TND sample due to selection bias. In the next section, we describe a new framework to account for unmeasured confounding in a TND setting, leveraging negative control exposure and outcome variables.

### 2.3 Tackling unmeasured confounding bias leveraging negative controls

#### 2.3.1 Negative control exposure (NCE) and the treatment confounding bridge function

As shown in Figure 1(e), suppose that one has observed a valid possibly vector-valued NCE, denoted as *Z*, which is a priori known to satisfy the following key independence conditions:

**Assumption 3** (NCE independence conditions). Z⫫(Y,S)|A,U,X.

Assumption 3 essentially states that any existing *Z*−*Y* association conditional on (*X, A*) in the target population must be a consequence of their respective association with *U*, therefore indicating the presence of confounding bias. Importantly, the NCE must *a priori* be known to have no causal effect on the infection status (Miao, Shi & Tchetgen Tchetgen 2018). Likewise, the association between *Z* and *S* conditional on (*X, A*) is completely due to their respective association with *U*. Figure 1(e) presents a graphical illustration of an NCE that satisfies Assumption 3.

Shi, Miao & Tchetgen Tchetgen (2020) provided some general guidelines and examples of how to select an NCE in different settings. In the Influenza VE setting, a candidate NCE can be vaccination status for the preceding year, or other vaccination status such as Tdap (Tetanus, Diphtheria, Pertussis) vaccine, as both are known to effectively provide no protection against the circulating flu strain in a given year. We emphasize that an appropriate NCE should have no direct effect on selection into the TND sample. In other words, the selected NCE should be irrelevant to the study’s inclusion/exclusion criteria other than through *U, X*. We now provide an intuitive description of our approach to leverage *Z* as an imperfect proxy of *U* for identification despite not directly observing *U*.

To motivate the rationale behind identification, ignore selection bias for now and suppose that *Q*(*A, U*) = *α*_0_ + *α*_1_*A* + *α*_2_*U*, also suppressing measured confounders *X*. Although *U* is unobserved, suppose further that *Z* satisfies *E*[*Z*|*A, U*] = *γ*_0_ + *γ*_1_*A* + *γ*_2_*U*. Then we have $U=E[\tilde{U}(A,Z)|A,U]$, where $\tilde{U}(A,Z)=\left(Z-\gamma_{0}-\gamma_{1}A\right)/\gamma_{2}$. Replacing *U* with $\tilde{U}(A,Z)\text{ in }Q(A,U)$, we get $q(A,Z)=\alpha_{0}+\alpha_{1}A+\alpha_{2}\tilde{U}(A,Z)$, which does not depend on unmeasured confounder *U* and can recover the inverse probability of vaccination from *Q*(*A, U*) = *E*[*q*(*A, Z*)|*A, U*]. If all parameters of *q* were known, it would naturally follow that the IPTW method in (1) can be recovered by

$$E[Y(a)]=E\{I(A=a)E[q(A,Z)|A,U]Y\}\overset{A.3}{=}E[I(A=a)q(A,Z)Y],$$

Therefore, *β*_0_ can be identified if the distribution of (*A, Y, Z*) in the target population is available provided that parameters indexing *q* can be identified. The above insight motivates the following assumption:

**Assumption 4** (treatment confounding bridge function). *There exists a function q*(*A, Z, X*) *that satisfies, for every a, u and x,*

(7) Q(A=a,U=u,X=x)=E[q(A,Z,X)|A=a,U=u,X=x]

A function *q* that satisfies (7) is called a treatment confounding bridge function, as it bridges the observed NCE with the unobserved propensity score (Cui et al. 2020). Below we give two examples where the integral equation (7) admits a closed form solution.

**Example 1.** *(Binary U and Z) Suppose that U is binary, and so is the NCE Z. For simplicity we suppress X. Write p*_*za.u*_ = *P*(*Z* = *z, A* = *a*|*U* = *u*)*. We prove in* Section A *of the* Supplementary Materials *that the treatment confounding bridge function q*(*a, z*) *has a closed form given by*

(8) $$q(a,z)=\left[p_{1a.1}-p_{1a.0}+\left(p_{0a.0}-p_{0a.1}-p_{1a.1}+p_{1a.0}\right)z\right]/\left(p_{0a.0}p_{1a.1}-p_{0a.1}p_{1a.0}\right).$$

The result can be similarly extended to polytomous Z.

**Example 2.** *(Continuous U and Z) Suppose the unmeasured confounder U and the NCE Z are both continuous. Further assume that*

$$A|U,X\sim Bernoulli\left({\left[1+\exp\left(-\mu_{0A}-\mu_{UA}U-\mu_{XA}X\right)\right]}^{-1}\right)$$

$$Z|A,U,X\sim N\left(\mu_{0Z}+\mu_{AZ}A+\mu_{UZ}U+\mu_{XZ}X,\sigma_{Z}^{2}\right).$$

*The treatment confounding bridge function q*(*A, Z, X*) *can then be shown equal to*

(9) $$q(A,Z,X)=1+\exp\left[{\left(-1\right)}^{A}\left(\tau_{0}+\tau_{1}A+\tau_{2}Z+\tau_{3}X\right)\right]$$

*where* $\tau_{0}=\mu_{0A}-\frac{\mu_{UA}\mu_{0Z}}{\mu_{UZ}}-\frac{\sigma_{z}^{2}\mu_{UA}^{2}}{2\mu_{UZ}^{2}}$, $\tau_{1}=\frac{\sigma_{z}^{2}\mu_{UA}^{2}}{\mu_{UZ}^{2}}-\frac{\mu_{UA}\mu_{AZ}}{\mu_{UZ}}$, $\tau_{2}=\mu_{UA}/\mu_{UZ}$, *and* $\;\tau_{3}=\mu_{XA}-\mu_{XZ}\mu_{UA}/\mu_{UZ}$.

Formally, Equation (7) defines a Fredholm integral equation of the first kind, with treatment confounding bridge function *q*(*A, Z, X*) as its solution (Cui et al. 2020). Heuristically, the existence of a solution requires that variation in *Z* induced by *U* is sufficiently correlated with variation in *A* induced by *U*. For instance, in Example 1, existence of a treatment confounding bridge function requires that the matrix *P*_*Z,A*|*U*_ is nonsingular. In Example 2, the existence of a treatment confounding bridge function amounts to the condition *μ*_*UZ*_ ≠ 0, which again requires Z⫫U∣A,X . Cui et al. (2020) provided formal conditions sufficient for the existence of the treatment confounding bridge function satisfying Equation (7). These conditions are reproduced for completeness in Section B of the Supplementary Materials.

Thus, under Assumption 4, we propose to construct a new unbiased estimating function for *β*_0_ by replacing *Q*(*A, U, X*) with *q*(*A, Z, X*) in *V*_0_(*A, Y, U, X*; *β*_0_).

**Theorem 1.** *(Moment restriction of β*_0_*) Under Assumptions 1–4, we have that*

$$E\left[V_{1}\left(A,Y,Z,X;\beta_{0}\right)|U,X,S=1\right]=0$$

*where V*_1_(*A, Y, Z, X*; *β*_0_) = (−1)^1–A^*q*(*A, Z, X*)*Y* exp(−*β*_0_*A*).

Theorem 1 immediately implies that

$$E\left[{\left(-1\right)}^{1-A}c(X)q(A,Z,X)Y\exp\left(-\beta_{0}A\right)|S=1\right]=0$$

for any function *c*(*X*). In practice, if one can consistently estimate the treatment confounding bridge function *q*(*A, Z, X*) with $\hat{q}(A,Z,X)$, then *β*_0_ can be estimated by solving the estimating equation

(10) $$\frac{1}{n}\sum_{i=1}^{n}{\left(-1\right)}^{1-A_{i}}c\left(X_{i}\right)\hat{q}\left(A_{i},Z_{i},X_{i}\right)Y_{i}\exp\left(-\beta_{0}A_{i}\right)=0,$$

for unidimensional *c*(·) ≠ 0, which results in the closed form estimator

$${\hat{\beta }}_{0}=\log\left(\frac{\sum c\left(X_{i}\right)\hat{q}\left(A_{i},Z_{i},X_{i}\right)A_{i}Y_{i}}{\sum c\left(X_{i}\right)\hat{q}\left(A_{i},Z_{i},X_{i}\right)\left(1-A_{i}\right)Y_{i}}\right).$$

Importantly, although (7) may not have a unique solution, any solution uniquely identifies the causal log RR *β*_0_ for a fixed function *c*(·). Furthermore, although the choice of *c*(·) does not impair unbiasedness of (10), it does impact efficiency of the resulting estimator ${\hat{\beta }}_{0}$. In practice, one may simply set *c*(*X*_*i*_) ≡ 1.

There remains the question of identifying and estimating the treatment confounding bridge function from the TND sample which we consider next.

#### 2.3.2 Negative control outcome (NCO) for identification of treatment confounding bridge function

For identification and estimation of *q*, we propose to leverage NCOs to construct feasible estimating equations for the treatment confounding bridge function as in Cui et al. (2020). Similar to NCEs, NCOs can be viewed as imperfect proxies of *U*. However, unlike NCEs, a valid NCO, denoted by *W*, is (i) known a priori not to be a causal effect of either the primary exposure *A* or the NCE *Z*; and (ii) is associated with (*A, Z*) conditional on *X* only to the extent that it is associated with *U*. Formally, we make the following assumption.

**Assumption 5.** *(NCO Independence Conditions).*

(*a*) W⫫A∣U,X; (*b*) W⫫Z∣A,U,X,Y; (*c*) S⫫Z∣A,U,X,W,Y.

Assumptions 5(a) and (b) formalize the requirement that neither the primary exposure nor NCE can have direct effects on the NCO. Assumption 5(c) complements Assumption 3 and states that conditioning on *W* in addition to (*A, U, X, Y*) does not alter the conditional independence of *Z* with *S*. General guidelines for selecting NCOs are discussed by Shi, Miao & Tchetgen Tchetgen (2020). In TND studies in which there may be multiple possible test-negative illnesses, an NCO may be selected as one or more of the test-negative illnesses. In flu VE studies, a candidate NCO can be an infection whose risk is not causally affected by either *A* or *Z*. For example, if the selected NCE is Tdap vaccination, then a potential NCO may be current-year respiratory syncytial virus infection, as its risk is unlikely to be affected by Influenza or Tdap vaccination. Recent outpatient visits for other acute illnesses can also serve as NCO, such as blepharitis, wrist/hand sprain, lipoma, ingrowing nail, etc. (Leung et al. 2011). In contrast with an NCE, an NCO can have a direct effect on the selection *S*. Figure 1(e) illustrates an NCO *W* that satisfies Assumptions 5(a) and (b).

Similar to Cui et al. (2020), we leverage an NCO as an additional proxy to identify the treatment confounding bridge function. However, a complication arises due to the lack of a random sample from the target population, a key requirement in the approach outlined in Cui et al. (2020). In general, it is not possible to obtain sufficient information about either the distribution of *W* or that of *U* in the target population from only the TND data without an additional structural assumption (Bareinboim & Pearl 2012). In the following, we avoid imposing such an additional structural assumption by leveraging an important feature of several infectious diseases such as Influenza and COVID-19; mainly that contracting such an infection at any point in time is a rare event in most target populations of interest, and therefore information from the target population relevant to estimating the treatment confounding bridge function can be recovered from the test-negative control group. Formally, we make the following rare disease assumption.

**Assumption 6** (Rare infection). *There exist a small positive number δ >* 0 *such that*

(11) P(Y=1|A=a,W=w,U=u,X=x)≤δ,  for almost every a,w,u,x

Assumption 6 states that infected subjects, whether vaccinated or not and regardless of their NCO, only constitute a small proportion of each (*U, X*) stratum in the target population. This assumption implies that $\frac{1}{1-\delta }\leq \frac{P(A,Z|U=u,X=x,Y=0)}{P(A,Z|U=u,X=x)}\leq 1-\delta$. Thus, under Assumptions 1, 3 and 6, P(A=a,Z=z|U=u,X=x)≈P(A=a,Z=z|U=u,X=x,Y=0,S=1) for all *a, z, x, u*. We now introduce a key property of the treatment confounding bridge function in Theorem 2.

**Theorem 2** (Identification of the treatment confounding bridge function). *Under Assumptions 1, 3, 4, 5, and 6, for a* = 0, 1 *we have that*

$$\frac{{\left(1-\delta \right)}^{3}}{P(A=a|W,X,Y=0,S=1)}<E[q(a,Z,X)|W,A=a,X,Y=0,S=1]\;<\frac{1}{{\left(1-\delta \right)}^{3}P(A=a|W,X,Y=0,S=1)}$$

Thus, provided *δ* is small, Theorem 2 suggests that an approximation to the treatment confounding bridge function can be obtained by solving the following integral equation involving only observed data

(12) $$E\left[q*(A,Z,|X)|W,A=a,X,Y=0,S=1\right]=1/P(A=a|W,X,Y=0,S=1)$$

provided a solution exists. Accordingly, hereafter suppose that the following assumption holds.

**Assumption 7** (Existence of a unique solution to (12)). *There exists a unique square-integrable function q*∗(*A, Z, X*) *that satisfies* (12).

Heuristically, uniqueness of a solution to (12) requires that variation in *W* is sufficiently informative about variation in *Z*, in the sense that there is no variation in *W* that is not associated with corresponding variation in *Z*. See Section D of the Supplementary Materials for further elaboration of completeness condition and Newey & Powell (2003), D’Haultfoeuille (2011) for related use of the assumption in the literature. Below we briefly illustrate Assumption 7 in the examples of Section 2.3.1.

**Example 1′.** *Suppose U and Z are both binary, and a binary NCO W is also observed. Let* $p_{za.w}^{\prime }=P(Z=z,A=a|W=w,Y=0,S=1)$ *for* z,a,w∈{0,1}, *then solving* *Equation* (12) *gives* $q*(a,z)=\left[p_{1a.1}^{\prime }-p_{1a.0}^{\prime }+\left(p_{0a.0}^{\prime }-p_{0a.1}^{\prime }-p_{1a.1}^{\prime }+p_{1a.0}^{\prime }\right)z\right]/\left(p_{0a.0}^{\prime }p_{1a.1}^{\prime }-p_{0a.1}^{\prime }p_{1a.0}^{\prime }\right)$. *The probabilities p***′**_*za.w*_ *can all be estimated from the study sample.*

We emphasize that the solution to Equation (12) is ultimately an approximation to the (non-identifiable) treatment confounding bridge function in the target population. The accuracy of this approximation relies on the extent to which the rare disease assumption holds in the target population of interest. We study the potential bias due to departure from this key assumption in Section E of the Supplementary Materials. We further observe that, under the null hypothesis of no vaccine effectiveness, or if *W* has no direct effects on *Y* or *S*, then the function *q*∗(*A, Z, X*) matches the treatment confounding bridge exactly, even if the disease is not rare, as stated in the corollary below.

**Corollary 1.** *Under the Assumptions of Theorem 1, Assumption 7, and the null hypothesis of no vaccine effect against infection, such that* Y⫫A|U,X, *then*

E[q(A,Z,X)|W,A=a,X,Y=0,S=1]=1/P(A=a|W,X,Y=0,S=1).

From Theorem 2, we immediately have the following corollary which provides a basis for estimation of *q*∗(*A, Z, X*) from the observed TND sample.

**Corollary 2.** *Under the conditions of Theorem 2, for any function m*(*W, A, X*)*, the solution q*∗(*A, Z, X*) *to* *Equation* (12) *also solves the population moment equation*

(13) $$E\left[m(W,A,X)q*(A,Z,X)-m(W,1,X)-m(W,0,A)|Y=0,S=1\right]=0.$$

In practice, a parametric model *q*∗(*A, Z, X*; *τ*) for the treatment confounding bridge function might be appropriate, where *τ* is an unknown finite dimensional parameter, Corollary 2 suggests one can then estimate *τ* by solving the estimating equation

(14) $$\frac{1}{n}\sum_{i=1}^{n}\left(1-Y_{i}\right)[m(W,A,X)q(A,Z,X;\hat{\tau })-m(W,1,X)-m(W,0,X)]=0,$$

where *m*(*W, A, X*) is a user-specified function with dimension no smaller than *τ* ‘s.

**Example 1′′.** *If Z and W are both binary, rather than solving the system of equations implied by* (12)*, one can instead specify a saturated model*

(15) $$q*(A,Z;\tau )=\tau_{0}+\tau_{1}Z+\tau_{2}A+\tau_{3}ZA$$

*and estimate τ* = (*τ*_0_*, τ*_1_*, τ*_2_*, τ*_3_)^*T*^ *by solving* (14) *with m*(*W, A*) = (1*, W, A, WA*)^*T*^. *Extension to Z and X with multiple categories is relatively straightforward*.

**Example 2**′. *In case of continuous* (*U, X, Z*)*, result* (9) *suggests the model*

(16) $$q*(A,Z,X;\tau )=1+\exp\left[{\left(-1\right)}^{A}\left(\tau_{0}+\tau_{1}A+\tau_{2}Z+\tau_{3}X\right)\right].$$

*If a univariate NCO W is available, we may solve* (14) *with m*(*W, A, X*) *defined as a vector including* (*W, A, X*)*, their high-order interactions and an intercept.–*

### 2.4 Estimation and Inference

In previous sections, we have defined the structural parameter of interest *β*_0_ as stratum-specific log risk ratio, introduced the treatment confounding bridge function as a key ingredient to identification of *β*_0_, and presented a strategy to estimate the treatment confounding bridge function leveraging an NCO. We summarize the steps of our estimation framework in Algorithm 1 and present the large-sample properties of the resulting estimator ($\hat{\beta }$, $\hat{\tau }$) in Theorem 3.

**Theorem 3** (Inference based on ($\hat{\beta }$, $\hat{\tau }$)). *Under Assumptions 1–7 and suitable regularity conditions provided in* Section F, *the estimator* ($\hat{\beta }$, $\hat{\tau }$) *Algorithm 1, or equivalently, the solution to the estimating equation* $\frac{1}{n}\sum_{i=1}^{n}G_{i}(\beta ,\tau )=0$ *is regular and asymptotically linear with the i-th influence function* $IF_{i}(\beta ,\tau )=-\Omega {\left(\beta ,\tau \right)}^{+}G_{i}(\beta ,\tau )$, *where* $\Omega {\left(\beta ,\tau \right)}^{+}$ *denotes the*

$$\bar{\underline{\begin{array}{l} \underline{\begin{array}{l} \text{Algorithm 1}\text{ Negative control method to estimate vaccine eectiveness from a test-negative}\\ \text{design}\;\;\;\;\;\;\;\;\;\;\;\;\;\;\; \end{array}}\\ 1:\;\text{Identify the variables in the data according to Figure 1(e), in particular the NCEs and}\\ \;\;\text{NCOs}.\\ 2\;:\;\text{Estimate the treatment confounding bridge function by solving Equation (14) with}\\ \;\;\text{a suitable parametric model }q*(A,Z,X;\tau )\;\text{and a user-specied function }m(W;\;A;X).\\ \;\;\text{Write }\hat{\tau }\;\text{as the resulting estimate of }\tau .\;\\ 3\;:\;\text{Estimate }\beta_{0}\text{ by}\\ \;\;\;\;\;\;\;\hat{\beta }=\log\left(\frac{\sum c\left(X_{i}\right)q^{*}\left(a,Z_{i},X_{i};\hat{\tau }\right)A_{i}Y_{i}}{\sum c\left(X_{i}\right)q^{*}\left(A_{i},Z_{i},X_{i};\hat{\tau }\right)\left(1-A_{i}\right)Y_{i}}\right)\\ \;\;where\;\hat{q}(A,Z,X)=q*(A,Z,X;\hat{\tau })\text{ and }c(\cdot )\;\text{is a user-specied one-dimensional function}. \end{array}}}$$

*Moore-Penrose inverse* Ω(β,τ),

$$G_{i}(\beta ,\tau )=\left(\begin{array}{c} {\left(-1\right)}^{1-A_{i}}c\left(X_{i}\right)q^{*}\left(A_{i},Z_{i},X_{i};\tau \right)Y_{i}\exp\left(-\beta A_{i}\right)\\ \left(1-Y_{i}\right)\left[m\left(W_{i},A_{i},X_{i}\right)q^{*}\left(A_{i},Z_{i},X_{i};\tau \right)-m\left(W_{i},1,X_{i}\right)-m\left(W_{i},0,X_{i}\right)\right] \end{array}\right)$$

*and* $\Omega (\beta ,\tau )=\left(E\left[\partial G_{i}(\beta ,\tau )/\partial \beta^{T}\right],E\left[\partial G_{i}(\beta ,\tau )/\partial \tau^{T}\right]\right)$.

Large-sample standard errors and confidence intervals follow from standard estimating equation theory (See Van der Vaart (2000) Theorem 5.21).

The estimator $\hat{\beta }$ and its standard error are constructed under Assumption 6. If Assumption 6 fails to hold, $\hat{\beta }$ may be biased and confidence intervals may not be well-calibrated. However, by Corollary 1, under the null hypothesis of no vaccine effect, the estimated *q*∗(*A, Z, X*) converges to the true treatment confounding bridge function and $\hat{\beta }$ is consistent for *β*_0_ = 0. This implies that while our methods are approximately asymptotically unbiased for rare infections, they provide a valid test of no vaccine causal effect even if the infection is not rare.

### 2.5 Estimating VE under treatment-induced selection

Thus far, unbiasedness of the estimating function *V*_0_ has crucially relied on Assumption 1 that *A* does not have a direct effect on *S*. In some settings, this assumption may be violated if an infected person who is vaccinated is on average more likely to present to the ER than an unvaccinated infected person with similar symptoms, so that treatment or vaccination-induced selection into the TND sample is said to be present. In such settings, the estimator $\hat{\beta }$ produced by Algorithm 1 may be severely biased. Crucially, we note that this form of selection bias can be present even in context of a randomized trial in which vaccination/treatment is known to the analyst, if the outcome is ascertained using a TND, for example in recent cluster-randomized test-negative design studies of community-level dengue intervention effectiveness (Anders et al. 2018, Jewell et al. 2019, Dufault & Jewell 2020, Wang et al. 2022). In this Section, we provide sufficient conditions for identification under treatment-induced selection. In this vein, consider the following assumptions:

**Assumption 1**′. *P*(*S* = 1 | *A* = *a, Y* = 1*, U, X*)*/P*(*S* = 1 | *A* = *a, Y* = 0*, U, X*) = exp(*h*(*U, X*)) *for a* = 0, 1.

That is, the risk ratio association between infection status and selection into the TND sample is independent of vaccination status. Furthermore,

**Assumption 2**′. *(No effect modification by confounders on the OR scale).*


$$\frac{P(Y=1|A=1,U,X)/P(Y=0|A=1,U,X)}{P(Y=1|A=0,U,X)/P(Y=0|A=0,U,X)}=\exp\left(\beta_{0}^{\prime }\right).$$

Recall that Assumption 2 posited a constant vaccination causal effect on the RR scale across levels of (*U, X*), while Assumption 3′ instead posits that the corresponding causal effect on the odds ratio scale is constant across levels of (*U, X*). In case of a rare infection in the target population, the OR and RR are approximately equal, in which case VE is well approximated by 1 – *OR*.

Furthermore, identification relies on the following modified definition of a treatment confounding bridge function:

**Assumption 4**′. *There exists a treatment confounding bridge function* $\tilde{q}$ *such that for a* = 0, 1,

(17) $$E[\tilde{q}(a,Z,X)|A=a,U,X]=1/P(A=a|U,X,Y=0,S=1)\;\;almost\;surely.$$

We now obtain the identification of the OR in the following theorem:

**Theorem 1**′. *Under Assumptions 1*′*, 2*′*, 3 and 4*′*, we have that*

$$E\left[{\tilde{V}}_{1}\left(A,Y,Z,X;\beta_{0}^{\prime }\right)|U,X,S=1\right]=0$$

*where* ${\tilde{V}}_{1}(A,Y,Z,X;\beta )$ *is defined as V*_1_ *in Theorem 1 except* with $\tilde{q}$ *replacing q.*

Importantly, the theorem establishes that the estimating function *V*_1_ previously developed in the paper can, under stated conditions, remain unbiased for the odds ratio association of vaccination with testing positive for the infection, even in the presence of treatment-induced selection into the TND sample.

Estimation of the treatment confounding bridge function $\tilde{q}(A,Z,X)$ requires a negative control outcome that satisfies:

**Assumption 5**′. *(NCO Independence Conditions)* W⫫(A,Z,S)∣U,X,Y.

In addition to Assumptions 5, this last assumption requires that neither *Y* nor *S* is a causal effect of *W*. Figure 1(f) illustrates a DAG that satisfies our assumptions regarding (*Z, W*). As can be verified in the graph, Assumption 6′ is needed to ensure that collider stratification bias induced by the path *A* → [*S* = 1] ← *W* upon conditioning on *S* = 1 is no longer present. Identification of the function $\tilde{q}$ is given below:

**Theorem 2**′. *Under assumptions 3, 4*′ *and 5*′, *for a* = 0, 1 *we have that*

$$E[\tilde{q}(a,Z,X)|A=a,W,X,Y=0,S=1]=1/P(A=a|W,X,Y=0,S=1)$$

As a result of Theorem 2′, the parameters in the treatment confounding bridge function can be estimated by solving moment equation (14).

In summary, the above discussion suggests that one can continue to use Algorithm 1 to estimate VE in presence of treatment induced selection bias, albeit on the OR scale and under a modified set of negative control conditions. Theorem 3 continues to apply.

As a side note, Assumption 1′ automatically holds under Assumption 1, hence the above results also apply to the setting in previous sections illustrated in Figure 1(e). We state this result in the following corollary.

**Corollary 3.** *Under Assumptions 1, 2*′*, 3 and 4*′*, we have*

$$E\left[{\tilde{V}}_{1}\left(A,Y,Z,X;\beta_{0}^{\prime }\right)|U,X,S=1\right]=0.$$

With Assumption 1, the treatment confounding bridge function $\tilde{q}$ can be estimated by solving the moment equation (14) either under Assumption 5′ and 7, or Assumptions 5, 6 and 7 as an approximation under the rare disease assumption. Corollary 3 leads to an interesting observation: under treatment-independent sampling (Assumption 1), the estimator $\hat{\beta }$ from Algorithm 1 can be viewed as either log RR or log OR, depending on the setting.

Finally, as Schnitzer (2022) pointed out, under the additional assumptions that (1) subjects included in the TND analysis who do not test positive for the infection in view are nevertheless subject to another infection, referred to as a test-negative infection, (2) test-positive and test-negative infections are mutually exclusive, and (3) vaccination of interest has no causal effect on the test-negative infection, the conditional odds ratio exp(*β*_0_) also identifies the conditional causal risk ratio. We further discussed this result in Section G of the Supplementary Materials.

## 3 Simulation Study

To assess the empirical performance of our proposed method, we perform simulation studies mimicking a TND with binary vaccination, infection, and testing. We generate a target population of *N* = 7, 000, 000 according to Figure 1(e). We consider two scenarios where *U*, *Z* and *W* are binary or continuous. In each scenario, we consider the true value of the log risk ratio *β*_0_ = log(0.2), log(0.5), log(0.7) and 0. We set baseline log risk *η*_0_ = log(0.01) so that the outcome is rare in the target population. The generated TND sample had sample size raging between *n* = 43, 000 and *n* = 52, 000.

For each scenario, we evaluated the performance of bias and coverage rates of 95% confidence intervals of the proposed NC estimator for *β*_0_ over 500 Monte Carlo samples. As the estimated treatment confounding bridge function in Algorithm 1 is only an approximation under Assumption 6, whose bias may affect the estimation for *β*_0_, we also include the NC-Oracle estimator that uses the true treatment confounding bridge function. For comparison, we included two standard estimators for *β*_0_ in test-negative designs, logistic regression (Bond et al. 2016) and the IPTW estimator (Schnitzer 2022), both of which do not account for bias due to the latent factor *U*. Table S1 in Section I of the Supplementary Materials shows the details of the data generating process and the methods we evaluated.

Figure S1 in Section I reports the bias of the four estimators we considered and coverage of their 95% corresponding confidence intervals. In both settings, both NC and NC-Oracle are essentially unbiased whereas logistic regression and IPTW give biased estimates in all scenarios. NC-Oracle exhibits slightly higher precision than NC, which implies that estimating the treatment confounding bridge function in the TND is only slightly more variable. Confidence intervals of NC and NC-Oracle both attain nominal coverage, whereas logistic regression and IPTW based confidence intervals undercover severely.

To investigate the performance of our method in case of a non-rare infection, we repeat the simulation under the same setup but increasing baseline log risk *η*_0_. In our simulation setting, exp(*η*_0_) equals *δ* in Assumption 6 that describes the prevalence of the infection outcome in the target population, which determines the bias of estimating the treatment confounding bridge function. The results are reported in Figures S2 and S3 in Section I of the Supplementary Materials. While the NC-Oracle estimator remains unbiased and maintained calibrated confidence intervals, the bias of the NC estimator increases with increasing *δ* and has confidence intervals that under-cover. Notably, the NC estimator remains unbiased with calibrated confidence intervals under the null hypothesis *β*_0_ = 0. Both logistic regression and IPTW estimators are severely biased.

## 4 Application

We applied our proposed method to a TND study of COVID-19 VE of two-dose Moderna vaccine (mRNA-1273), two-dose Pfizer-BioNTech vaccine (BNT162b2), and single-dose Johnson & Johnson’s Janssen vaccine (Ad26.COV2.S) against SARS-Cov-2 infection nested in the University of Michigan Health System. The selected study sample included patients who interacted with the University of Michigan Health System and experienced COVID-19 symptoms, had suspected exposure to COVID-19 virus, or sought to screen for SARS-Cov-2 infection, between April 5, 2021 and December 7, 2021. In addition, selected test-positive subjects had at least one positive lab test for SARS-Cov-2 infection after April 5. Vaccination history was obtained through electronic health records. A study subject was considered fully vaccinated if they received at least one dose of Johnson & Johnson’s Janssen vaccine or at least two doses of Moderna or Pfizer-BioNTech vaccine. If a subject tested positive before or within 14 days after their first dose of Janssen vaccine or within 14 days after their second dose of Moderna or Pfizer-BioNTech vaccine, they were considered unvaccinated (Moline et al. 2021).

We selected immunization visits before December 2020 for NCE since COVID-19 vaccines were unavailable before December 2020 and prior immunization was unlikely to affect SARS-Cov-2 infection risk; nor that of the selected NCOs we describe next. For NCO, we selected a binary indicator of having at least one of the following “negative control outcome” conditions after April 5, 2021: arm/leg cellulitis, eye/ear disorder, gastro-esophageal disease, atopic dermatitis, and injuries. Such candidate NCE and NCO are likely to satisfy the requisite conditional independence conditions of valid negative control variables and to be related to a patient’s latent HSB. We adjusted for age group (*<*18, between 18 and 60, or ≥ 60), gender, race (white or non-white), Charlson comorbidity score ≥ 3, and the calendar month of a test-positive subject’s first positive COVID test or a test-negative subject’s last COVID test. Table S2 in Section J of the Supplementary Materials summarizes the distribution of negative control variables, demographic variables and SARS-Cov-2 infection among vaccinated and unvaccinated subjects.

Because NCE is expected not to be associated with either the outcome or NCO in a fully adjusted analysis unless there is unmeasured confounding, we first fit regression models to detect presence of residual confounding bias. Conditioning on the baseline covariates, in both vaccinated and unvaccinated groups, NCE was significantly associated with SARS-Cov-2 infection (*p <* 0.001) and NCO (*p <* 0.001) in corresponding adjusted logistic regression models, suggesting the presence of hidden bias (See Section J Table S3 and S4 of the Supplementary Materials).

We implemented Algorithm 1 to estimate VE. We specified a linear model for the treatment confounding bridge function with an interaction term between COVID-19 vaccination and the NCE, and set the function *m* to include an intercept term, COVID-19 vaccination, the NCO, and baseline covariates, as well as all two-way interactions. For comparison, we also implemented the adjusted logistic regression model and the IPTW estimator of marginal risk ratio of Schnitzer (2022), where propensity score is estimated with a logistic regression model.

Table 1 shows the estimated VE and 95% confidence intervals for the three estimators. There is significant evidence of hidden bias as summarized in Tables S3 and S4. The VE estimates with our double NC method are notably higher than those of standard logistic regression and IPTW estimator for all three vaccines.

Recent TND studies estimated the mRNA COVID-19 vaccine (Pfizer-BioNTech and Moderna) effectiveness ranging between 80% and 98% against lab-confirmed SARS-COV-2 infection of different variants (Dagan et al. 2021, Bruxvoort et al. 2021, Israel et al. 2021). Our NC approach provided VE estimates that are closer to these prior studies. We hypothesize that the standard logistic regression and IPTW estimator underestimate the VE by overlooking residual confounding bias due to HSB and related factors, which our proposed double NC approach appears to control to some extent.

## 5 Discussion

In this article, we have introduced a statistical approach leveraging negative control variables to account for hidden bias due to residual confounding and/or selection mechanism in a test-negative design, both of which have raised major concerns (Sullivan et al. 2016, Schnitzer 2022). Negative control variables abound in practice, such as vaccination history which is routinely collected in insurance claims and electronic health records. Hence the proposed method may be particularly useful in such real-world settings to obtain improved estimates of vaccine effectiveness. Beyond TND, our method is also applicable to other study designs with outcome-dependent sampling, such as a case-control study for a rare disease where unmeasured confounding is of concern. With simple modifications, our approach can also be applied to settings with polytomous treatment and/or outcome, as discussed in Section K of the Supplementary Materials.

The TND is a challenging setting in causal inference where selection bias and unmeasured confounding co-exist, the selection is outcome-dependent, and unmeasured confounders also impact selection. Jackson et al. (2018) performed simulation studies to evaluate the selection bias of VE estimate by logistic regression and concluded that selection bias due to healthcare-seeking behavior is unlikely to be meaningful in practice. However, their simulation study did not consider the effect of healthcare-seeking behavior on the infection outcome. Didelez et al. (2010) used graphical models to study conditions for the estimability of the odds ratio and testability of the hypothesis of null causal effect under outcome-dependent sampling, but they did not consider the setting where the unmeasured confounders also affect selection. A widely adopted approach to adjust for selection bias is to reweight each observation’s contribution by their corresponding inverse probability of selection into the sample (Hernán et al. 2004), but such weights are unlikely to be available in most TNDs without access to a random sample from the target population and accurate measurements of latent healthcare-seeking behavior. Bareinboim et al. (2014) formally showed that causal effects cannot be recovered from outcome-dependent sampling without an additional assumption. Bareinboim & Pearl (2012) also showed that the causal odds ratio cannot be recovered under both confounding and selection bias. We have established that, however, progress can be made under a semiparametric multiplicative model, provided the outcome is rare in the target population, and that double negative control variables are available. To this end, this article showcases the potential power of negative control methods and proximal causal inference in epidemiologic research (Shi, Miao & Tchetgen Tchetgen 2020, Tchetgen Tchetgen et al. 2020).

We primarily focused on outpatient TND studies, where recruitment is restricted to subjects who seek care voluntarily. TNDs have also been applied to inpatient settings for studying VE against, for example, flu hospitalization (Foppa et al. 2016, Feng et al. 2016). In inpatient TNDs, differential access to healthcare and underlying health characteristics between vaccinated and unvaccinated subjects are likely the main source of confounding bias (Feng et al. 2016). Our methods are still applicable in such settings, but negative control variables should be selected to be relevant to this source of unmeasured confounding. For example, previous vaccination and hospitalization outside of the flu season or hospitalization due to other flu-like illness are viable candidate NCE and NCO, respectively (Jackson et al. 2006).

Our approach is also suitable for post-market TND studies where real-world VE is of interest and vaccination history is obtained retrospectively, possibly through electronic health records. For vaccine efficacy in a controlled trial setting, Wang et al. (2022) recently developed estimation and inference of RR in cluster-randomized TND, aiming to correct for bias due to intervention-induced confounding by HSB induced due to unblinding. They proposed a log-RR estimator which corrects for selection bias by leveraging a valid test-negative outcome, under an assumption that either (i) the vaccine does not have a causal effect in the population, and the causal impact of vaccination on selection is equal for test-positive and -negative subsamples; or (ii) among care seekers, the incidence of test-negative outcomes does not differ between vaccinated and unvaccinated, and the intervention effect among care seekers is generalizable to the whole population. Even under randomization, identification conditions given in Section 2.5 are neither stronger nor weaker than those of Wang et al. (2022) described above, as neither set of assumptions appear to imply the other. An important advantage of our proposed methods is that they can be used to account for selection bias in a TND study irrespective of randomization.

Our methods target RR as a measure of VE instead of the more common OR (Jackson et al. 2006, Sullivan et al. 2016). These two measures are approximately equal for rare infections as described in Section L of the Supplementary Materials. Schnitzer (2022) recently considered estimation of a marginal causal RR in the TND sample and justified the use of an inverse probability of treatment weighted (IPTW) estimator in a setting in which an unmeasured common cause of infection and selection into the TND sample does not cause vaccination (and thus there is no unmeasured confounding). Instead, our methods allow for an unmeasured common cause of vaccination, infection, and selection into the TND sample; however, in order to estimate a causal RR, we invoke both, an assumption of no effect modification by an unmeasured confounder, and a rare-disease condition. As we establish, the latter assumption can be relaxed to test the null hypothesis of no causal effect of the vaccine on infection risk. In Section 2.5, we establish that under a homogeneous OR condition, and an alternative definition of the treatment bridge function, our methods can identify a causal effect of the vaccine on the OR scale without invoking the rare disease condition.

Throughout the article, we have assumed diagnostic tests are accurate and individuals who seek care are sparsely distributed, such that the vaccination of a given subject in the TND sample does not protect another study subject from infection, i.e. there is no interference in the TND sample, a common assumption in TND literature. This latter assumption may be violated if members of the same households are present in the ER, in which case block interference must be accounted for using results from interference literature (Hudgens & Halloran 2008, Tchetgen Tchetgen & VanderWeele 2012).

We have proposed a parametric approach to estimate treatment confounding bridge function. While we have provided examples where certain parametric models are appropriate, in general, such a parametric approach may result in model misspecification bias. Nonparametric methods that have been developed for proximal causal inference, such as kernel machine learning (Ghassami, Ying, Shpitser, Tchetgen Tchetgen et al. 2021, Mastouri et al. 2021) may be adapted to our setting. Since the treatment confounding bridge function is defined by a conditional moment restriction, another potential nonparametric approach is sieve generalized method of moments that uses basis functions to approximate the nuisance function (Ai & Chen 2003, Chen 2007). We leave these topics for future research.

## Supplementary Material

Supplement 1

## Figures and Tables

**Table 1:** Estimated VE and 95% confidence intervals of the negative control estimator, logistic regression and IPTW estimator with the University of Michigan Health System data.

	Negative control	Logistic regression	IPTW
Pfizer-BioNTech	71.6% (68.9%, 74.0%)	67.0% (64.5%, 69.4%)	60.5% (57.9%, 62.9%)
Moderna	86.8% (84.7%, 88.6%)	75.1% (72.5%, 77.5%)	66.7% (62.3%, 70.6%)
Janssen (J & J)	66.0% (54.5%, 74.6%)	56.0% (47.9%, 62.9%)	47.5% (27.4%, 62.1%)
