# Supplementary material for: Double Negative Control Inference in Test-Negative Design Studies of Vaccine Effectiveness
Source: ArXiv. 2022 Mar 23:arXiv:2203.12509v3. Preprint. [Version 3] (PMC8963685)
Supplement: Supplementary file 1 [file NIHPP2203.12509v3-supplement-1.pdf]

Supplementary materials to "Double Negative Control Inference in Test-Negative Design Studies of Vaccine Effectiveness"

A Proofs and derivations

A.1 Proof of Proposition 1

The result immediately follows if we can show that

$$E\left[(-1)^{1-A}\frac{1}{P(A|U,X)}\exp(-\beta_0 A)\middle|U,X,Y=1,S=1\right]=0. \tag{18}$$

By Assumption 1, the left-hand side of (18) equals

$$E\left[(-1)^{1-A}\frac{1}{P(A|U,X)}\exp(-\beta_0 A)\middle|U,X,Y=1\right].$$

We further have

$$\begin{aligned}
&E\left[(-1)^{1-A}\frac{1}{P(A|U,X)}\exp(-\beta_0 A)\middle|U,X,Y=1\right]\\
=&\sum_{a=0}^{1}(-1)^{1-a}\frac{1}{P(A=a|U,X)}\exp(-\beta_0 a)P(A=a|U,X,Y=1)\\
=&\sum_{a=0}^{1}(-1)^{1-a}\frac{1}{P(A=a|U,X)}\exp(-\beta_0 a)\frac{P(A=a|U,X)P(Y=1|A=a,U,X)}{P(Y=1|U,X)}\\
\overset{A.2}{=}&\sum_{a=0}^{1}(-1)^{1-a}\exp(-\beta_0 a)\frac{\exp(\beta_0 a)P(Y=1|A=0,U,X)}{P(Y=1|U,X)}\\
=&\sum_{a=0}^{1}(-1)^{1-a}\frac{P(Y=1|A=0,U,X)}{P(Y=1|U,X)}\\
=&0.
\end{aligned}$$

A.2 Proof of Theorem 1

It suffices to show that

$$E[(-1)^{1-A}q(A,Z,X)\exp(-\beta_0 A)\mid U,X,Y=1,S=1]=0.$$

By Assumption 1, the left-hand side is

$$\begin{aligned}
&E[e^{-\beta_0 A}(-1)^{1-A}q(A,Z,X) \mid U,X,Y=1] \\
=&E\{e^{-\beta_0 A}(-1)^{1-A}E[q(A,Z,X) \mid A,U,X,Y=1] \mid U,X,Y=1\} \\
\overset{A.3}{=}&E\{e^{-\beta_0 A}(-1)^{1-A}E[q(A,Z,X) \mid A,U,X] \mid U,X,Y=1\} \\
\overset{A.4}{=}&E\{e^{-\beta_0 A}(-1)^{1-A}\frac{1}{P(A|U,X)} \mid U,X,Y=1\} \\
=&0
\end{aligned}$$

The last equality is proved as before.

A.3 Derivation of the treatment confounding bridge function in Example 2

By Assumption 4, the treatment confounding bridge function $q(A,Z,X)$ should satisfy

$$E[q(A,Z,X)|U=u,A=a,X=x] = \frac{1}{P(A=a|U=u,X=x)}$$

for all a, u and X.

We write $q(A,Z,X) = 1 + r(Z,A,X)$, then

$$E[r(Z,a,X)|U,A=a,X] = \frac{1-P(A=a|U,X)}{P(A=a|U,X)}.$$

Consider

$$r(Z,A,X) = \exp((-1)^A(\tau_0 + \tau_1 A + \tau_2 Z + \tau_3 X)),$$

then

$$\begin{aligned}
&\frac{P(A=1|U=u,X=x)}{1-P(A=1|U=u,X=x)} \\
=&\int r(z,0,x)f(z|u,x,A=0)dz \\
=&\int \exp(\tau_0+\tau_2 z+\tau_3 x)\frac{1}{\sqrt{2\pi\sigma_z^2}}\exp\left(-\frac{(z-\mu_{0z}-\mu_{UZ}u-\mu_{XZ}x)^2}{2\sigma_z^2}\right)dz \\
=&\exp(\tau_0+\tau_3 x)\exp\left(\tau_2(\mu_{0Z}+\mu_{UZ}u+\mu_{XZ}x)+\frac{\sigma_z^2\tau_2^2}{2}\right) \\
=&\exp(\tau_0+\tau_2\mu_{0Z}+\frac{\sigma_z^2\tau_2^2}{2}+\tau_2\mu_{UZ}u+(\tau_3+\mu_{XZ}\tau_2)x)
\end{aligned}$$

and

$$
\begin{aligned}
&\frac{1-P(A=1|U=u,X=x)}{P(A=1|U=u,X=x)}\\
=&\int r(z,1,x)f(z|u,x,A=1)dz\\
=&\int \exp(-\tau_0-\tau_1-\tau_2 z-\tau_3 x)\frac{1}{\sqrt{2\pi\sigma_z^2}}\exp\left(-\frac{(z-\mu_{0Z}-\mu_{AZ}-\mu_{UZ}u-\mu_{XZ}x)^2}{2\sigma_z^2}\right)dz\\
=&\exp(-\tau_0-\tau_1-\tau_3 x)\exp\left(-\tau_2(\mu_{0Z}+\mu_{AZ}+\mu_{UZ}u+\mu_{XZ}x)+\frac{\sigma_z^2\tau_2^2}{2}\right)\\
=&\exp(-\tau_0-\tau_1-\tau_2\mu_{0Z}-\tau_2\mu_{AZ}+\frac{\sigma_z^2\tau_2^2}{2}-\tau_2\mu_{UZ}u-(\tau_3+\tau_2\mu_{XZ})x)
\end{aligned}
$$

This requires that

$$
\tau_1+\tau_2\mu_{AZ}=\sigma_z^2\tau_2^2
$$

Because

$$
P(A=1|U,X)=\text{expit}(\mu_{0A}+\mu_{UA}U+\mu_{XA}X),
$$

we conclude the parameters in the bridge function are

$$
\tau_2=\mu_{UA}/\mu_{UZ},
$$

$$
\begin{aligned}
\tau_3&=\mu_{XA}-\mu_{XZ}\tau_2=\mu_{XA}-\mu_{XZ}\mu_{UA}/\mu_{UZ}\\
\tau_1&=\sigma_z^2\tau_2^2-\tau_2\mu_{AZ}=\frac{\sigma_z^2\mu_{UA}^2}{\mu_{UZ}^2}-\frac{\mu_{UA}\mu_{AZ}}{\mu_{UZ}}\\
\tau_0&=\mu_{0A}-\tau_2\mu_{0Z}-\frac{\sigma_z^2\alpha_2^2}{2}=\mu_{0A}-\frac{\mu_{UA}\mu_{0Z}}{\mu_{UZ}}-\frac{\sigma_z^2\mu_{UA}^2}{2\mu_{UZ}^2}
\end{aligned}
$$

A.4 Proof of Theorem 2 and Corollary 1

We first introduce a few properties due to the rare disease assumption:

Lemma 1. *Under Assumptions 1, 3, 5 and 6, for every a, w, u and x, we have*

(a)

$$
\begin{aligned}
&P(Y=1|W=w,U=u,X=x)<\delta, \qquad P(Y=1|A=a,U=u,X=x)<\delta,\\
&P(Y=1|U=u,X=x)<\delta.
\end{aligned}
$$

(b)

$$
1-\delta<\frac{P(A=a|U=u,X=x,Y=0,S=1)}{P(A=a|U=u,X=x)}<\frac{1}{1-\delta}
$$

(c)

$$
(1-\delta)^2<\frac{P(A=a|W=w,U=u,X=x,Y=0,S=1)}{P(A=a|U=u,X=x,Y=0,S=1)}<\frac{1}{(1-\delta)^2}.
$$

(d)

$$f(z|A=a,U=u,X=x)=f(z|W=w,A=a,U=u,X=x,Y=0,S=1).$$

Proof. (a) For every a, w, u and x, we have

$$\begin{aligned}
&P(Y=1|W=w,U=u,X=x)\\
&=\sum_a P(Y=1|A=a,W=w,U=u,X=x)P(A=a|W=w,U=u,X=x)\\
&\overset{A.6}{<}\delta\sum_a P(A=a|W=w,U=u,X=x)\\
&=\delta.
\end{aligned}$$

The rest follows similarly.

(b) For every a, u and x, we have

$$P(A=a|U=u,X=x,Y=0)=P(A=a|U=u,X=x)\times\frac{P(Y=0|A=a,U=u,X=x)}{P(Y=0|U=u,X=x)}.$$

By Lemma 1(a), we have

$$1-\delta<\frac{P(Y=0|A=a,U=u,X=x)}{P(Y=0|U=u,X=x)}=\frac{P(A=a|U=u,X=x,Y=0)}{P(A=a|U=u,X=x)}<\frac{1}{1-\delta}.$$

The result follows by noticing that $P(A=a|U=u,X=x,Y=0)=P(A=a|U=u,X=x,Y=0,S=1)$ due to Assumption 1.

(c) For every a, w, u and x, we have

$$\begin{aligned}
&P(A=a|W=w,U=u,X=x,Y=0)\\
&=P(A=a|W=w,U=u,X=x)\times\frac{P(Y=0|A=a,W=w,U=u,X=x)}{P(Y=0|W=w,U=u,X=x)}\\
&\overset{A.5(a)}{=}P(A=a|U=u,X=x)\times\frac{P(Y=0|A=a,W=w,U=u,X=x)}{P(Y=0|W=w,U=u,X=x)}\\
&=P(A=a|U=u,X=x,Y=0)\times\frac{P(Y=0|U=u,X=x)}{P(Y=0|A=a,U=u,X=x)}\\
&\quad\times\frac{P(Y=0|A=a,W=w,U=u,X=x)}{P(Y=0|W=w,U=u,X=x)}
\end{aligned}$$

By Lemma 1(a), we have

$$1-\delta<\frac{P(Y=0|U=u,X=x)}{P(Y=0|A=a,U=u,X=x)}<\frac{1}{1-\delta}$$

and

$$1-\delta < \frac{P(Y=0|A=a,W=w,U=u,X=x)}{P(Y=0|W=w,U=u,X=x)} < \frac{1}{1-\delta}.$$

We therefore have

$$(1-\delta)^2 < \frac{P(A=a|W=w,U=u,X=x,Y=0)}{P(A=a|U=u,X=x,Y=0)} < \frac{1}{(1-\delta)^2}.$$

Finally, by Assumptions 1 and 5(c), we have

$$P(A=a|U=u,X=x,Y=0) = P(A=a|U=u,X=x,Y=0,S=1),$$
$$P(A=a|W=w,U=u,X=x,Y=0) = P(A=a|W=w,U=u,X=x,Y=0,S=1).$$

We conclude that

$$(1-\delta)^2 < \frac{P(A=a|W=w,U=u,X=x,Y=0,S=1)}{P(A=a|U=u,X=x,Y=0,S=1)} < \frac{1}{(1-\delta)^2}.$$

(d)

$$\begin{aligned} & f(z|W=w,A=a,U=u,X=x,Y=0,S=1) \\ \overset{A.5(c)}{=} & f(z|W=w,A=a,U=u,X=x,Y=0) \\ \overset{A.5(b)}{=} & f(z|A=a,U=u,X=x) \end{aligned}$$

□

Therefore, we have

$$
\begin{aligned}
&E\{q(a,Z,X)|A=a,W,X,Y=0,S=1\}\\
=&\int q(a,z,X)f(z|A=a,W,X,Y=0,S=1)\,\mathrm{d}z\\
=&\int\int q(a,z,X)f(z|A=a,W,U=u,X,Y=0,S=1)f(u|A=a,W,X,Y=0,S=1)\,\mathrm{d}z\,\mathrm{d}u\\
\overset{L.1(d)}{=}&\int\left\{\int q(a,z,X)f(z|A=a,U=u,X)\,\mathrm{d}u\right\}f(u|A=a,W,X,Y=0,S=1)\,\mathrm{d}z\\
\overset{A.4}{=}&\int\frac{1}{P(A=a|U=u,X)}f(u|A=a,W,X,Y=0,S=1)du\\
\overset{L.1(b)}{<}&\frac{1}{1-\delta}\int\frac{1}{P(A=a|U=u,X,Y=0,S=1)}f(u|A=a,W,X,Y=0,S=1)du\\
\overset{L.1(c)}{<}&\frac{1}{(1-\delta)^3}\int\frac{1}{P(A=a|W,U=u,X,Y=0,S=1)}f(u|A=a,W,X,Y=0,S=1)du\\
=&\frac{1}{(1-\delta)^3}\int\frac{f(u|W,X,Y=0,S=1)f(u|A=a,W,X,Y=0,S=1)}{P(A=a|W,X,Y=0,S=1)f(u|A=a,W,X,Y=0,S=1)}\,\mathrm{d}u\\
=&\frac{1}{(1-\delta)^3}\frac{1}{P(A=a|W,X,Y=0,S=1)}\int f(u|W,X,Y=0,S=1)\,\mathrm{d}u\\
=&\frac{1}{(1-\delta)^3}\frac{1}{P(A=a|W,X,Y=0,S=1)}
\end{aligned}
$$

and

$$
\begin{aligned}
&E\{q(a,Z,X)|A=a,W,X,Y=0,S=1\}\\
=&\int\frac{1}{P(A=a|U=u,X)}f(u|A=a,W,X,Y=0,S=1)\,\mathrm{d}u\\
\overset{L.1(b)}{>}&(1-\delta)\int\frac{1}{P(A=a|U=u,X,Y=0,S=1)}f(u|A=a,W,X,Y=0,S=1)\,\mathrm{d}u\\
\overset{L.1(c)}{>}&(1-\delta)^3\int\frac{1}{P(A=a|W,U=u,X,Y=0,S=1)}f(u|A=a,W,X,Y=0,S=1)\,\mathrm{d}u\\
=&(1-\delta)^3\int\frac{f(u|W,X,Y=0,S=1)f(u|A=a,W,X,Y=0,S=1)}{P(A=a|W,X,Y=0,S=1)f(u|A=a,W,X,Y=0,S=1)}\,\mathrm{d}u\\
=&(1-\delta)^3\frac{1}{P(A=a|W,X,Y=0,S=1)}\int f(u|W,X,Y=0,S=1)\,\mathrm{d}u\\
=&(1-\delta)^3\frac{1}{P(A=a|W,X,Y=0,S=1)}.
\end{aligned}
$$

To prove Corollary 1, we have

$$
\begin{aligned}
&E\{q(a,Z,X)|A=a,W,X,Y=0,S=1\}\\
=&\int \frac{1}{P(A=a|U=u,X)} f(u|A=a,W,X,Y=0,S=1)\,\mathrm{d}u\\
=&\int \frac{P(A=a|U=u,X,Y=0,W,S=1)}{P(A=a|U=u,X)P(A=a|W,X,Y=0,S=1)} f(u|W,X,Y=0,S=1)\,\mathrm{d}u\\
\overset{A.5}{=}&\int \frac{P(A=a|U=u,X,Y=0,W)}{P(A=a|U=u,X)P(A=a|W,X,Y=0,S=1)} f(u|W,X,Y=0,S=1)\,\mathrm{d}u
\end{aligned}
$$

If $A \perp\!\!\!\perp Y|U,X$, then together with Assumption 5 we have $P(A=a|U=u,X,Y=0,W) = P(A=a|U=u,X)$, whereby the above equals

$$
\frac{1}{P(A=a|W,X,Y=0,S=1)}\int f(u|W,X,Y=0,S=1)du = \frac{1}{P(A=a|W,X,Y=0,S=1)}.
$$

A.5 Proof of Corollary 2 and further discussion

We first prove equation (13)

$$
\begin{aligned}
&E[m(W,A,X)q^*(A,Z,X) - m(W,1,X) - m(W,0,X)|Y=0,S=1]\\
=&E\left\{m(W,A,X)E\left[q^*(A,Z,X)|W,A,X,Y=0,S=1\right] - m(W,1,X) - m(W,0,X)|Y=0,S=1\right\}\\
\overset{(12)}{=}&E\left\{m(W,A,X)\frac{1}{P(A|W,X,Y=0,S=1)} - m(W,1,X) - m(W,0,X)|Y=0,S=1\right\}\\
=&E\Big\{E\left[m(W,A,X)\frac{1}{P(A|W,X,Y=0,S=1)}|W,X,Y=0,S=1\right] - \\
&\quad m(W,1,X) - m(W,0,X)|Y=0,S=1\Big\}\\
=&E\left\{m(W,1,X) + m(W,0,X) - m(W,1,X) - m(W,0,X)|Y=0,S=1\right\}\\
=&\,0
\end{aligned}
$$

In fact, one can show that any regular and asymptotically normal estimator of τ that satisfies (12) has influence function of the form

$$
\begin{aligned}
IF(W,Z,A,X) = -\left\{E\left[\frac{\partial q(A,Z,X;\tau)}{\partial\tau}\bigg|_{\tau=\tau_0} m(W,A,X)(1-Y)\bigg|S=1\right]\right\}^{-1}(1-Y)\\
\Big(m(W,A,X)q(A,Z,X) - m(W,1,X) - m(W,0,X)\Big)
\end{aligned}
$$

for an arbitrary function $m(W,A,X)$. Therefore, any regular and asymptotically normal estimator of τ corresponds to the solution of the estimating equation (14) for some function $m(W,A,X)$.

To prove this result, we see that for any parametric submodel that satisfies (12) and is

indexed by s such that the true distribution corresponds to $s=0$, we have

$$E_s\left\{(1-Y)\left[q(A,Z,X;\tau_s)-\frac{1}{f_s(A|W,X,Y=0,S=1)}\right]m(W,A,X)\middle|S=1\right\}=0$$

and thus

$$\partial E_s\left\{(1-Y)\left[q(A,Z,X;\tau_s)-\frac{1}{f_s(A|W,X,Y=0,S=1)}\right]m(W,A,X)\middle|S=1\right\}/\partial s\Big|_{s=0}=0.$$

Note that

$$\begin{aligned}
&\frac{\partial}{\partial s}E_s[(1-Y)q(A,Z,X;\tau_s)m(W,A,X)|S=1]\\
=&E\left[\frac{\partial q(A,Z,X;\tau)}{\partial\tau}\Big|_{\tau=\tau_0}m(W,A,X)(1-Y)\middle|S=1\right]\frac{\partial\tau_s}{\partial s}\Big|_{s=0}+\\
&E[(1-Y)q(A,Z,X)m(W,A,X)S(O|S=1)|S=1]
\end{aligned}$$

and

$$\begin{aligned}
&\frac{\partial}{\partial s}E_s\left[\frac{(1-Y)m(W,A,X)}{f_s(A|W,X,Y=0,S=1)}\middle|S=1\right]\\
=&E\left[-(1-Y)m(W,A,X)\frac{\frac{\partial}{\partial s}f_s(A|W,X,Y=0,S=1)}{f^2(A|W,X,Y=0,S=1)}\middle|S=1\right]+\\
&E\left[\frac{m(W,A,X)(1-Y)}{f(A|W,X,Y=0,S=1)}S(W,A,X,Y=0|S=1)\middle|S=1\right]\\
=&E\left[-\frac{m(W,A,X)(1-Y)}{f(A|W,X,Y=0,S=1)}S(A|W,X,Y=0,S=1)\middle|S=1\right]+\\
&E\left[\frac{m(W,A,X)(1-Y)}{f(A|W,X,Y=0,S=1)}S(W,A,X,Y=0|S=1)\middle|S=1\right]\\
=&E\left[\frac{m(W,A,X)(1-Y)}{f(A|W,X,Y=0,S=1)}S(W,X,Y=0|S=1)\middle|S=1\right]\\
=&E\left[(1-Y)\{m(W,1,X)-m(W,0,X)\}S(W,X,Y=0|S=1)\middle|S=1\right]\\
=&E\left[(1-Y)\{m(W,1,X)-m(W,0,X)\}S(O|S=1)\middle|S=1\right]
\end{aligned}$$

Rearranging the terms, we have

$$\frac{\partial \tau_s}{\partial s}\bigg|_{s=0} =$$

$$E\bigg[-\bigg\{E\bigg[\frac{\partial q(A,Z,X;\tau)}{\partial \tau}\bigg|_{\tau=\tau_0} m(W,A,X)(1-Y)\bigg|S=1\bigg]\bigg\}^{-1}(1-Y)$$
$$\Big(m(W,A,X)q(A,Z,X) - m(W,1,X) - m(W,0,X)\Big)S(O|S=1)\bigg|S=1\bigg]$$

A.6 Proof of Theorem 1′

Lemma 2. *Under Assumptions 2′, 3′ and 6′, we have*

$$\begin{aligned}&P(A=a,Y=y|U,W,X,S=1)\\&\quad=\frac{1}{\xi}P(A=a|Y=0,U,W,X,S=1)P(Y=y|A=0,U,W,X,S=1)\exp(\beta_0' ay)\end{aligned}$$

where $\xi = \sum_{a^*,y^*} P(A=a^*|Y=0,U,W,X,S=1)P(Y=y^*|A=0,U,W,X,S=1)\exp(\beta_0' a^* y^*)$.

Proof. Note that

$$\begin{aligned}&\frac{P(Y=1|A=1,U,W,X,S=1)P(Y=0|A=0,U,W,X,S=1)}{P(Y=1|A=0,U,W,X,S=1)P(Y=0|A=1,U,W,X,S=1)}\\&\overset{A.6'}{=}\frac{P(Y=1|A=1,U,X,S=1)P(Y=0|A=0,U,X,S=1)}{P(Y=1|A=0,U,X,S=1)P(Y=0|A=1,U,X,S=1)}\\&=\exp(\beta_0')\times\frac{P(S=1|Y=1,A=1,U,X)}{P(S=1|Y=0,A=1,U,X)}\times\frac{P(S=1|Y=0,S=0,U,X)}{P(S=1|Y=1,S=0,U,X)}\\&\overset{A.2'}{=}\exp(\beta_0')\times\exp(h(U,X))\times\exp(-h(U,X))\\&=\exp(\beta_0')\end{aligned}$$

The result follows after Chen (2003). □

To prove Theorem 1′, we need to show

$$E[(-1)^{1-A}c(X)q(A,Z,X)Y\exp(-\beta_a A)|S=1]=0$$

It suffices to prove that

$$E[(-1)^{1-A}q(A,Z,X)Y\exp(-\beta_a A)|U,X,S=1]=0.$$

The left-hand side is

$$\sum_{a,y} \int (-1)^{1-a} y \exp(-\beta_0' a) q(a,z,X) P(A=a, Y=y|U,X,S=1) f(z|U,X,A=a,Y=y,S=1) dz$$

$$\stackrel{L.2}{=} \sum_{a,y} \int (-1)^{1-a} y \exp(-\beta_0' a) q(a,z,X) \times$$

$$\frac{1}{\xi} P(A=a|Y=0,U,X,S=1) P(Y=y|A=0,U,X,S=1) \exp(\beta_0' a y) \times$$

$$f(z|U,X,A=a,Y=y,S=1) dz$$

$$= \sum_{a} \int (-1)^{1-a} \exp(-\beta_0' a) q(a,z,X) \times$$

$$\frac{1}{c} P(A=a|Y=0,U,X,S=1) P(Y=1|A=0,U,X,S=1) \exp(\beta_0' a) \times$$

$$f(z|U,X,A=a,Y=1,S=1) dz$$

$$= \sum_{a} \frac{(-1)^{1-a}}{\xi} P(A=a|Y=0,U,X,S=1) P(Y=1|A=0,U,X,S=1) \times$$

$$\int q(a,z,X) f(z|U,X,A=a,Y=1,S=1) dz$$

$$\stackrel{A.4'}{=} \sum_{a} \frac{(-1)^{1-a}}{\xi} P(A=a|Y=0,U,X,S=1) P(Y=1|A=0,U,X,S=1) \times$$

$$\frac{1}{P(A=a|Y=0,U,X,S=1)}$$

$$= \sum_{a} \frac{(-1)^{1-a}}{\xi} P(Y=1|A=0,U,X,S=1)$$

$$=0$$

A.7 Proof of Theorem 2′

$$
\begin{aligned}
& E[\tilde{q}(a,Z,X)|A=a,W,X,Y=0,S=1] \\
=& E\left\{E[\tilde{q}(a,Z,X)|A=a,U,W,X,Y=0,S=1]|A=a,W,X,Y=0,S=1\right\} \\
\overset{A.5'}{=}& E\left\{E[\tilde{q}(a,Z,X)|A=a,U,X,Y=0,S=1]|A=a,W,X,Y=0,S=1\right\} \\
\overset{A.3'}{=}& E\left\{E[\tilde{q}(a,Z,X)|A=a,U,X]|A=a,W,X,Y=0,S=1\right\} \\
\overset{A.4'}{=}& E\left\{\frac{1}{P(A=a|U,X,Y=0,S=1)}|A=a,W,X,Y=0,S=1\right\} \\
=& \int \frac{1}{P(A=a|U=u,X,Y=0,S=1)} f(u|A=a,W,X,Y=0,S=1)du \\
=& \int \frac{1}{P(A=a|U=u,X,Y=0,S=1)} \times \\
& \quad \frac{f(u|W,X,Y=0,S=1)P(A=a|U=u,W,X,Y=0,S=1)}{P(A=a|W,X,Y=0,S=1)}du \\
\overset{A.5'}{=}& \int \frac{1}{P(A=a|U=u,X,Y=0,S=1)} \times \\
& \quad \frac{f(u|W,X,Y=0,S=1)P(A=a|U=u,X,Y=0,S=1)}{P(A=a|W,X,Y=0,S=1)}du \\
=& \frac{1}{P(A=a|W,X,Y=0,S=1)} \int f(u|W,X,Y=0,S=1)du \\
=& \frac{1}{P(A=a|W,X,Y=0,S=1)}
\end{aligned}
$$

B Existence of solutions to (7)

In this section, we provide the conditions of existence of solutions to (7). The conditions for (12) can be similarly derived. The results in this section directly adapted from Appendix B of Cui et al. (2020).

Let $L^2\{F(t)\}$ denote the Hilbert space of all square-integrable functions of t with respect to distribution function $F(t)$, equiped with inner product $\langle g_1, g_2 \rangle = \int g_1(t) g_2(t) dF(t)$. Let $T_{a,x}$ denote the operator $L^2\{F(z|a,x)\} \to L^2\{F(u|a,x)\}$, $T_{a,x}q = E[q(Z)|A = a, U = u, X = x]$ and let $(\lambda_{a,x,n}, \varphi_{a,x,n}, \phi_{a,x,n})$ denote a singular value decomposition of $T_{a,x}$. The solution to (7) exists if:

(1) $\int\int f(z|a,u,x) f(u|a,z,x) dz du < \infty$;

(2) $\int P^{-2}(A = a|U = u, X = x) f(u|a,x) du < \infty$;

(3) $\sum_{n=1}^{\infty} \lambda_{a,x,n}^{-2} |\langle P^{-1}(A = a|U = u, X = x), \phi_{a,x,n} \rangle|^2 < \infty$.

C Treatmeng bridge function and estimation with categorical NCE, NCO and unmeasured confounders.

We first consider the case where U and Z are both binary. The integral equation (7) can then be written as $\sum_{z=0}^{1} q(a,z)P(Z=z|U=u,A=a) = P(A=a|U=u)^{-1}$, or equivalently, $\sum_{z=0}^{1} p_{za.u}q(a,z) = 1$ for each $a,u \in \{0,1\}$, where $p_{za.u} = P(Z=z, A=a|U=u)$. Therefore, the treatment confounding bridge function $q(a,z)$ solves the linear equation system

$$P_{Z,A|U}\begin{pmatrix} q(a,0) \\ q(a,1) \end{pmatrix} = \begin{pmatrix} 1 \\ 1 \end{pmatrix}, \text{ where } P_{Z,A|U} = \begin{pmatrix} p_{0a.0} & p_{1a.0} \\ p_{0a.1} & p_{1a.1} \end{pmatrix}.$$

If the matrix $P_{Z,A|U}$ is invertible, then $q(a,z)$ has a closed form solution given by

$$q(a,z) = [p_{1a.1} - p_{1a.0} + (p_{0a.0} - p_{0a.1} - p_{1a.1} + p_{1a.0})z] \,/\, (p_{0a.0}p_{1a.1} - p_{0a.1}p_{1a.0})\,. \tag{19}$$

We then consider a categorical unmeasured confounder U with categories $u_1,\ldots,u_J$ and NCE Z with categories $z_1,\ldots,z_I$. We assume there are no other covariates X. We write $p_{ia.k} = P(Z=z_i, A=a|U=u_j)$ for $i=1,\ldots,I$, $a=0,1$, and $j=1,\ldots,J$.

Similar to before, the treatment confounding bridge function $q(a,z)$ should satisfy

$$\sum_{i=1}^{I} p_{ia.j}q(a,z_i) = 1 \tag{20}$$

for all i,j. Therefore, any solution of the following equation system, if exists, is a treatment confounding bridge function:

$$\begin{aligned} p_{1a.1}q(a,z_1) + p_{2a.1}q(a,z_2) + \cdots + p_{Ia.1}q(a,z_I) = 1 \\ p_{1a.2}q(a,z_1) + p_{2a.2}q(a,z_2) + \cdots + p_{Ia.2}q(a,z_I) = 1 \\ \cdots \\ p_{1a.J}q(a,z_1) + p_{2a.J}q(a,z_2) + \cdots + p_{Ia.J}q(a,z_I) = 1 \end{aligned} \tag{21}$$

for $a = 0,1$. We denote the probability matrix

$$P_{Z,A|U} = \begin{pmatrix} p_{1a.1} & p_{2a.1} & \cdots & p_{Ia.1} \\ p_{1a.2} & p_{2a.2} & \cdots & p_{Ia.2} \\ \vdots & & & \vdots \\ p_{1a.J} & p_{2a.J} & \cdots & p_{Ia.J} \end{pmatrix}. \tag{22}$$

A treatment confounding bridge function exists if the matrix $P_{Z,A|U}$ is invertible.

Suppose besides a categorical NCE Z with levels $z_1,\ldots,z_I$, we also have a categorical NCO W with levels $w_1,\ldots,w_K$. The integral equation (12) is equivalent to the linear equation system

$$\begin{array}{c} p'_{1a.1}q^*(a,z_1)+p'_{2a.1}q^*(a,z_2)+\cdots+p'_{Ia.1}q^*(a,z_I)=1 \\ p'_{1a.2}q^*(a,z_1)+p'_{2a.2}q^*(a,z_2)+\cdots+p'_{Ia.2}q^*(a,z_I)=1 \\ \cdots \\ p'_{1a.K}q^*(a,z_1)+p'_{2a.K}q^*(a,z_2)+\cdots+p'_{Ia.K}q^*(a,z_I)=1 \end{array} \tag{23}$$

for $a = 0, 1$, where $p'_{ia.k} = P(Z = z_i, A = a|W = w_k, Y = 0, S = 1)$. We denote that matrix

$$P'(a) = \begin{pmatrix} p'_{1a.1} & p'_{2a.1} & \cdots & p'_{Ia.1} \\ p'_{1a.2} & p'_{2a.2} & \cdots & p'_{Ia.2} \\ \vdots & & & \vdots \\ p'_{1a.K} & p'_{2a.K} & \cdots & p'_{Ia.K} \end{pmatrix}. \tag{24}$$

Then Assumption 7 is equivalent to the condition that $P'(a)$ is invertible with $I = K$ for $a = 0, 1$, in which case (12) has a unique solution

$$(q^*(a, z_1), \ldots, q^*(a, z_I))^T = [P'(a)]^{-1}\mathbf{1}_I.$$

The probabilities $p'_{ia.k}$'s can all be estimated from the study data.

D Discussion of Assumption 7

Similar to (7), Equation (12) defines a Fredholm integral equation of the first kind, yet only involves observed data. Although a treatment confounding bridge function $q(A, Z, X)$ must satisfy (12), there is no guarantee that solving (12) gives a treatment confounding bridge function if multiple solutions exist. When a solution to (12) exists, we give the following assumptions that guarantee the uniqueness of solution.

Assumption 8 (Completeness)**.**

(a) For any square-integrable function g, if $E[g(Z)|A, U, X] = 0$ almost surely, then $g(Z) = 0$ almost surely;

(b) For any square-integrable function h, if $E[h(U)|A, W, X, Y = 0, S = 1]$ almost surely, then $h(U) = 0$ almost surely.

Intuitively, Assumption 8 is a statement on the information contained in the unmeasured confounders vs. in the negative control variables – Assumption 8(a) requires the confounders U are informative enough about Z in the target population and Assumption 8(b) requires the NCO W is informative enough about U in the subgroup $Y = 0, S = 1$, so that no information is lost when taking the two conditional expectations. Completeness conditions similar to Assumption 8 were originally introduced by Lehmann and Scheffe to identify the so-called unbiase minimum risk estimator (Lehmann & Scheffé 2012*a*,*b*). In econometrics and causal inference literature, completeness conditions have been employed to achieve identifiability for a variety of nonparametric or semiparametric models, such as instrumental variable regression (Newey & Powell 2003, D'Haultfoeuille 2011), measurement error models (Hu & Schennach 2008), synthetic control (Shi et al. 2021), and previous works in negative control methods (Miao, Shi & Tchetgen Tchetgen 2018, Cui et al. 2020, Ying et al. 2021). The completeness condition holds for a wide range of distributions. Newey & Powell (2003) and D'Haultfoeuille (2011) provided justifications in exponential families and discrete distributions with finite support. Andrews (2017) constructed a broad class of bivariate distributions that satisfy the completeness condition.

Assumption 8 have several immediate consequences:

Proposition 2. *(a) Under Assumptions 3, 5 and 8, for any square integrable function g such that $E[g(Z)|A, W, X, S = 0, Y = 1] = 0$ almost surely, then $g(Z) = 0$ almost surely.*

(b) Under Assumptions 8(a) and 1-4, the treatment confounding bridge function is unique. That is, if two square-integrable functions $q(A, Z, X)$ and $q_1(A, Z, X)$ satisfy

$$E[q(a, Z, x)|A = a, U = u, X = x] = E[q_1(a, Z, x)|A = a, U = u, X = x]$$
$$= \frac{1}{P(A = a|U = u, X = x)}$$

for all a, u, x almost surely, then $q(A, Z, X) = q_1(A, Z, X)$ almost surely.

(c) Under Assumptions 3, 5, 7 and 8, Equation (12) *has a unique solution* $q^*(A, Z, X)$.

We prove Proposition 2 below. Proposition 2 states that the completeness conditions in Assumption 8 and the definitions of the negative control variables lead to a third completeness condition that only involves the study data. Proposition 2 states the uniqueness of the treatment confounding bridge function, $q(A, Z, X)$, and the solution to (12), $q^*(A, Z, X)$. The function $q^*(A, Z, X)$ can therefore be identified from the study data alone and is a good approximation of $q(A, Z, X)$ by Theorem 2.

Proof. (a) Suppose a square-integrable function $g(Z)$ satisfies

$$E[g(Z)|A, W, X, Y = 0, S = 1] = 0 \quad \text{almost surely.}$$

The left-hand side equals

$$\begin{aligned}
&E\{E[g(Z)|U, A, W, X, Y = 0, S = 1]|A, W, X, Y = 0, S = 1\}\\
\overset{A.5}{=}&E\{E[g(Z)|U, A, X, Y = 0, S = 1]|A, W, X, Y = 0, S = 1\}\\
\overset{A.3}{=}&E\{E[g(Z)|U, A, X]|A, W, X, Y = 0, S = 1\}
\end{aligned}$$

By Assumption 8(b), $E[g(Z)|U, A, X] = 0$ almost surely. Then by Assumption 8(a), $g(Z) = 0$ almost surely.

(b) We have

$$E[q(A, Z, X) - q_1(A, Z, X)|A, U, X] = 0$$

almost surely. By Assumtion 8(a), we have

$$q(A, Z, X) - q_1(A, Z, X) = 0$$

almost surely, or $q(A, Z, X) = q_1(A, Z, X)$ almost surely.

(c) If two square-integrable functions $q^*(A, Z, X) = q_1^*(A, Z, X)$ satisfies

$$\begin{aligned}
&E[q^*(A, Z, X)|A, W, X, Y = 0, S = 1] = E[q_1^*(A, Z, X)|A, W, X, Y = 0, S = 1]\\
=&\frac{1}{P(A|W, X, Y = 0, S = 1)}
\end{aligned}$$

almost surely, then

$$E\{[q^*(A, Z, X) - q_1^*(A, Z, X)]|A, W, X, Y = 0, S = 1\} = 0.$$

Under Assumptions 3, 5 and 8, Proposition 2(a) holds and therefore

$$q^*(A, Z, X) = q_1^*(A, Z, X)$$

almost surely.

□

E Discussion on the rare disease assumption 6

Throughout this section, we set $c(X) = 1$.

We first describe a crucial identity that links the log risk ratio β_0 and the treatment confounding bridge function $q(A, Z, X)$.

Lemma 3. *Under Assumptions 1, 2 and 4, we have*

$$\beta_0 = \log\left(\frac{E[q(A,Z,X)I(A=1,Y=1)|S=1]}{E[q(A,Z,X)I(A=0,Y=1)|S=1]}\right) \tag{25}$$

Proof. The right hand side of (25) is

$$\begin{aligned}
&\log\left(\frac{E[q(A,Z,X)I(A=1,Y=1)|S=1]}{E[q(A,Z,X)I(A=0,Y=1)|S=1]}\right)\\
=&\log\left(\frac{E[q(A,Z,X)|A=1,Y=1,S=1]}{E[q(A,Z,X)|A=0,Y=1,S=1]}\right)+\log\left(\frac{P(Y=1,A=1|S=1)}{P(Y=1,A=0|S=1)}\right)
\end{aligned}$$

Note that for $a = 0, 1$,

$$\begin{aligned}
&E[q(A,Z,X)|A=a,Y=1,S=1]\\
=&E\left\{E[q(A,Z,X)|U,X,A=a,Y=1,S=1]|A=a,Y=1,S=1\right\}\\
\overset{A.1}{=}&E\left\{E[q(A,Z,X)|U,X,A=a]|A=a,Y=1,S=1\right\}\\
\overset{A.4}{=}&E\left\{\frac{1}{P(A=a|U,X)}\middle|A=a,Y=1,S=1\right\}\\
=&\int\frac{1}{P(A=a|U=u,X=x)}f(u,x|A=a,Y=1,S=1)\,\mathrm{d}u\,\mathrm{d}x\\
=&\int\frac{1}{P(A=a|U=u,X=x)}\times\\
&\frac{P(A=a|U=u,X=x)P(Y=1|A=a,U=u,X=x)P(S=1|Y=1,A=a,U=u,X=x)}{P(A=a,Y=1,S=1)}\,\mathrm{d}u\,\mathrm{d}x\\
\overset{A.1,A.2}{=}&\int\frac{\exp(\beta_0 a)P(Y=1|A=0,U=u,X=x)P(S=1|Y=1,U=u,X=x)}{P(A=a,Y=1,S=1)}\,\mathrm{d}u\,\mathrm{d}x\\
=&\frac{\exp(\beta_0 a)}{P(A=a,Y=1,S=1)}\int P(Y=1|A=0,U=u,X=x)P(S=1|Y=1,U=u,X=x)\,\mathrm{d}u\,\mathrm{d}x.
\end{aligned}$$

Therefore, the right-hand side of (25) equals

$$\begin{aligned}
&\beta_0+\log\left(\frac{P(A=0,Y=1,S=1)}{P(A=1,Y=1,S=1)}\right)+\log\left(\frac{P(Y=1,A=1|S=1)}{P(Y=1,A=0|S=1)}\right)\\
=&\beta_0+\log\left(\frac{P(A=0,Y=1|S=1)P(S=1)}{P(A=1,Y=1|S=1)P(S=1)}\right)+\log\left(\frac{P(Y=1,A=1|S=1)}{P(Y=1,A=0|S=1)}\right)\\
=&\beta_0
\end{aligned}$$

□

Let q^* be the function that satisfies (12):

$$E[q^*(a,Z,X)|A=a,W,X,Y=0,S=1]=\frac{1}{P(A=a|U=u,X,Y=0,S=1)}.$$

We introduce an additional regularity condition:

Assumption 9 (Uniform continuity)**.** *For any fixed positive square-integrable function $g(U)$ and a small positive number $0<\eta<1$, there exists some $0<\gamma=\gamma(g,\eta)<0$ such that $1-\gamma<\frac{E[g_1(U)|A,W,X,Y=0,S=1]}{E[g(U)|A,W,X,Y=0,S=1]}<\frac{1}{1-\gamma}$ a.e. implies $1-\eta<\frac{g_1(U)}{g(U)}<\frac{1}{1-\eta}$ a.e., where $g_1(U)$ is a positive square integrable function.*

Assumption 9 requires that the inverse mapping of $g\to E[g(U)|A,W,X,Y=0,S=1]$ is sufficiently smooth. By Theorem 2 and Assumption 7, we have

$$\begin{aligned}(1-\delta)^3 &< \frac{E[q^*(A,Z,X)|A,W,X,Y=0,S=1]}{E[q(A,Z,X)|A,W,X,Y=0,S=1]}\\ &= \frac{E\{E[q^*(A,Z,X)|A,W,U,X,Y=0,S=1]|A,W,X,Y=0,S=1\}}{E\{E[q(A,Z,X)|A,W,U,X,Y=0,S=1]|A,W,X,Y=0,S=1\}}\\ &\overset{A.5}{=} \frac{E\{E[q^*(A,Z,X)|A,U,X]|A,W,X,Y=0,S=1\}}{E\{E[q(A,Z,X)|A,U,X]|A,W,X,Y=0,S=1\}}\\ &\overset{A.4}{=} \frac{E\{E[q^*(A,Z,X)|A,U,X]|A,W,X,Y=0,S=1\}}{E\{E[\frac{1}{P(A|U,X)}|A,U,X]|A,W,X,Y=0,S=1\}}\\ &< \frac{1}{(1-\delta)^3}\end{aligned}$$

By Assumption 9, this implies

$$\frac{1-\eta(\delta)}{P(A|U,X)}<E[q^*(A,Z,X)|A,U,X]<\frac{1}{(1-\eta(\delta))P(A|U,X)}. \tag{26}$$

The constant $\eta(\delta)$ is determined by the smoothness of the inverse mapping of $g\mapsto E[g(U)|A,W,X,Y=0,S=1]$.

$$E[q^*(A,Z,X)|U=u,X,Y=0,S=1]=\frac{1}{P(A=a|U=u,X,Y=0,S=1)} \tag{27}$$

for almost all a and u. Let

$$\begin{aligned}\beta_0^* &= \log\left(\frac{E[q^*(A,Z,X)AY|S=1]}{E[q^*(A,Z,X)(1-A)Y|S=1]}\right)\\ &= \log\left(\frac{E[q^*(A,Z,X)|A=1,Y=1,S=1]}{E[q^*(A,Z,X)|A=0,Y=1,S=1]}\right)+\log\left(\frac{P(Y=1,A=1|S=1)}{P(Y=1,A=0|S=1)}\right).\end{aligned}$$

Under mild regularity, the estimator $\widehat{\beta}$ is regular and asymptotically linear for β_0^*, and

therefore

$$\widehat{\beta} = \beta_0^* + O_p(1/\sqrt{n}) = \beta_0 + (\beta_0^* - \beta_0) + O_p(1/\sqrt{n}).$$

It suffices to study

$$\beta_0^* - \beta_0 = \log\left(\frac{E[q^*(A,Z,X)|A=1,Y=1,S=1]}{E[q^*(A,Z,X)|A=0,Y=1,S=1]}\right) - \log\left(\frac{E[q(A,Z,X)|A=1,Y=1,S=1]}{E[q(A,Z,X)|A=0,Y=1,S=1]}\right)$$

Notice that

$$\begin{aligned}
& E[q^*(A,Z,X)|A=a,Y=1,S=1] \\
=& E\{E[q^*(A,Z,X)|A=a,U,X,Y=1,S=1]|A=a,Y=1,S=1\} \\
\overset{A.3}{=}& E\{E[q^*(A,Z,X)|A=a,U,X]|A=a,Y=1,S=1\} \\
\overset{A.3}{=}& E\{E[q^*(A,Z,X)|A=a,U,X,Y=0]|A=a,Y=1,S=1\} \\
\overset{(26)}{<}& \frac{1}{1-\eta(\delta)} E\left\{\frac{1}{P(A=a|U,X)}\middle| A=a,Y=1,S=1\right\} \\
\overset{A.4}{=}& \frac{1}{1-\eta(\delta)} E\left\{E[q(A,Z,X)|A,U,X]\middle| A=a,Y=1,S=1\right\} \\
\overset{A.3}{=}& \frac{1}{1-\eta(\delta)} E\left\{E[q(A,Z,X)|A,U,X,Y=1,S=1]\middle| A=a,Y=1,S=1\right\} \\
=& \frac{1}{1-\eta(\delta)} E[q(A,Z,X)|A=a,Y=1,S=1]
\end{aligned}$$

and similarly,

$$E[q^*(A,Z,X)|A=a,Y=1,S=1] > (1-\eta(\delta))E[q(A,Z,X)|A=a,Y=1,S=1].$$

Therefore, we have

$$1-\eta(\delta) < \frac{E[q^*(A,Z,X)|A=a,Y=1,S=1]}{E[q(A,Z,X)|A=a,Y=1,S=1]} < \frac{1}{1-\eta(\delta)}.$$

We conclude that

$$2\log(1-\eta(\delta)) = \log((1-\epsilon)^2) < \beta_0^* - \beta_0 < \log\left(\frac{1}{(1-\epsilon)^2}\right) = -2\log(1-\epsilon)$$

and thus

$$|\widehat{\beta} - \beta_0| < -2\log(1-\eta(\delta)) + O_p(\frac{1}{\sqrt{n}}).$$

F Regularity conditions and proof of Theorem 3

We denote τ_0^* as the true value of τ such that $q(A, Z, X; \tau_0^*) = q^*(A, Z, X)$. We will give the regularity conditions and proof that $(\widehat{\beta}, \widehat{\tau})$ is a regular and asymptotically linear estimator of (β_0^*, τ_0^*). Here β_0^* and q^* are the biased versions of β_0 and q defined in Section E, respectively, although the biases are negligible when the infection is rare. Following Section E, $\widehat{\beta}$ is also a regular and asymptotically linear estimator of β_0 if

$$\sup_{a,w,u,x} P(Y = 1 | A = a, W = w, U = u, X = x) < \delta_n,$$

Assumption 9 holds and

$$\log(1 - \eta(\delta_n)) = o_p(\frac{1}{\sqrt{n}}).$$

A set of regularity conditions are

R.1 The function $\tau \mapsto q(A, Z, X; \tau)$ is Lipschitz in a neighborhood of τ_0^*; that is, for every τ_1 and τ_2 in a neighborhood of τ_0 and a measurable function $\dot{q}(A, Z, X)$ with $E[\dot{q}(A, Z, X)] < \infty$, we have $\|q(A, Z, X; \tau_1) - q(A, Z, X; \tau_2)\| \leq \dot{q}(A, Z, X)\|\tau_1 - \tau_2\|$;

R.2 $E[q(A, Z, X; \tau_0^*)^2] < \infty$ and $E[m(W, A, X)^2] < \infty$;

R.3 The function $\tau \mapsto q(A, Z, X; \tau)$ is differentiable at τ_0^*. The derivative matrix $\Omega(\beta_0^*, \tau^*)$ is nonsingular;

R.4 $\frac{1}{n} \sum_{i=1}^n G_i(\widehat{\beta}, \widehat{\tau}) = o_p(n^{-1/2})$ and $(\widehat{\beta}, \widehat{\tau}) \xrightarrow{p} (\beta_0^*, \tau_0^*)$.

The condition R.1 and the fact that $\beta \mapsto \exp(-\beta A)$ is Lipschitz in a neighborhood of β_0^* imply the function $(\beta, \tau) \to M_i(\beta, \tau)$ is Lipschitz in a neighborhood of (β_0^*, τ_0^*) for every i. The remaining proof follows Van der Vaart (2000) Theorem 5.21.

G Connection between the conditional odds ratio and causal conditional risk ratio

When the test-positive and the test-negative infections are mutually exclusive, we define a categorical variable I such that $I = 2$ indicates the test-positive infection, $I = 1$ indicates the test-negative infection, and $I = 0$ indicates no infections. The conditional odds ratio of $I = 2$ vs. $I = 1$ is

$$\psi_{OR}(U, X) = \frac{P(I = 2 \mid A = 1, U, X) P(I = 1 \mid A = 0, U, X)}{P(I = 2 \mid A = 0, U, X) P(I = 1 \mid A = 1, U, X)}.$$

Further assume that the treatment has no effect on the test-negative infection:

$$P(I = 1 \mid A = 1, U, X) = P(I = 0 \mid A = 0, U, X),$$

then

$$\psi_{OR}(U, X) = \frac{P(I = 2 \mid A = 1, U, X)}{P(I = 2 \mid A = 0, U, X)},$$

the risk ratio of vaccination A on $I = 2$. Finally, assume the following standard identification assumptions hold:

(1) (Consistency) $I(a) = a$ if $A = a$ for $a = 0, 1$;

(2) (Exchangeability) $A \perp\!\!\!\perp I(a) \mid U, X$ for $a = 0, 1$;

(3) (Positivity) $0 < P(A = a \mid U, X)$ almost surely for $a = 0, 1$.

In particular, the consistency assumption requires no interference between the individuals' treatment and outcome. Then the conditional risk ratio is also the conditional causal risk ratio given (U, X):

$$\frac{P(I = 2 \mid A = 1, U, X)}{P(I = 2 \mid A = 0, U, X)} = \frac{P(I(1) = 2 \mid A = 1, U, X)}{P(I(0) = 2 \mid A = 0, U, X)} = \frac{P(I(1) = 2 \mid U, X)}{P(I(0) = 2 \mid U, X)}.$$

Finally, under the Assumption 10' that $\exp(\beta_0') = \psi_{OR}(u, x)$ for every u, x, the marginal causal risk ratio is

$$\begin{aligned}
\frac{P(I(1) = 2)}{P(I(0) = 2)} &= \frac{\int P(I(1) = 2 \mid U = u, X = x) f(u, x) \, \mathrm{d}u \, \mathrm{d}x}{\int P(I(0) = 2 \mid U = u, X = x) f(u, x) \, \mathrm{d}u \, \mathrm{d}x} \\
&= \frac{\int P(I(0) = 2 \mid U = u, X = x) \exp(\beta_0') f(u, x) \, \mathrm{d}u \, \mathrm{d}x}{\int P(I(0) = 2 \mid U = u, X = x) f(u, x) \, \mathrm{d}u \, \mathrm{d}x} \\
&= \exp(\beta_0').
\end{aligned}$$

H Accounting for effect modification by measured confounders

So far we have operated under Assumption 2 that VE is constant across levels of (U, X). As we now show, this assumption can be relaxed to allow for potential effect modification with respect to X without compromising identification. This extension is particularly important because empirical evidence has indeed suggested that flu vaccine effectiveness may vary across sex and age groups (Chambers et al. 2018); and similar effect heterogeneity is of key interest in case of COVID-19 (Fernández Villalobos et al. 2021).

Instead of Assumption 2, we consider a less stringent assumption:

Assumption 10 (No effect modification by unmeasured confounders)**.**

$$P(Y = 1|A = a, U, X) = \exp(\beta_0(X)a)g(U, X) \tag{28}$$

where $\beta_0(x)$ are $g(u, x)$ are unknown functions of x and u, x respectively.

Under standard assumptions of consistency, ignorability (given U, X) and positivity (Hernán MA 2020), Assumption 10 further implies that $\beta_0(x) = E[Y(1)|X = x]/E[Y(0)|X = x]$, i.e. the conditional causal RR as a function of x. Similar to Theorem 1, we have:

Theorem 4. *Under Assumptions 1, 3, 4 and 10, for an arbitrary function $c(\cdot)$ we have that*

$$E[V_3(A, Y, Z, X; \beta_0)|S = 1] = 0,$$

where $V_3(A, Y, Z, X; \beta_0) = (-1)^{1-A}c(X)q(A, Z, X)\exp(-\beta_0(X)A)$.

The proof of Theorem 4 is identical to that of Theorem 1 with $\beta_0 A$ replaced with $\beta_0(X)A$. Identification and estimation of the treatment confounding bridge function are also essentially identical to that of Corollary 2. Therefore, it is straightforward to extend Algorithm 1 to allow effect modification by measured confounders.

Algorithm 2 below describes a straightforward extension of Algorithm 1 to estimation the conditional vaccine effectiveness $VE(x) = 1 - \exp(\beta_0(x))$ under Assumption 10 and a parametric model $\beta_0(X; \alpha)$ indexed by a finite-dimensional parameter α.

We describe the large-sample properties of the estimator $(\widehat{\alpha}, \widehat{\tau})$ in the theorem below.

Theorem 5 (Inference of $(\widehat{\alpha}, \widehat{\tau})$)**.** *Under Assumptions ??- 1, 3-4, 5, 10 and suitable regularity conditions listed at the end of this section, the estimator $(\widehat{\alpha}, \widehat{\tau})$ in Algorithm 2, or equivalently, the solution to the estimating equation $\frac{1}{n}\sum_{i=1}^{n} \tilde{G}_i(\alpha, \tau) = 0$ is regular and asymptotically linear with influence function*

$$\widetilde{IF}(\alpha, \tau) = -\left[\tilde{\Omega}(\alpha, \tau)^T \tilde{\Omega}(\alpha, \tau)\right]^{-1} \tilde{\Omega}(\alpha, \tau)^T \tilde{G}_i(\alpha, \tau),$$

where

$$\tilde{G}_i(\alpha, \tau) = \begin{pmatrix} (-1)^{1-A_i}c(X_i)q^*(A_i, Z_i, X_i; \tau)Y_i \exp(-\beta_0(X; \alpha)A_i) \\ (1 - Y_i)\left[m(W_i, A_i, X_i)q^*(A_i, Z_i, X_i; \tau) - m(W_i, 1, X_i) - m(W_i, 0, X_i)\right] \end{pmatrix}$$

Algorithm 2 Negative control method to estimate conditional vaccine effectiveness from a test-negative design

1: Identify the variables in the data according to Figure 1(c), in particular the NCEs and NCOs.
2: Estimate the treatment confounding bridge function by solving the equation (14) with a suitable parametric model $q^*(A, Z, X; \tau)$ and a user-specified function $m(W, A, X)$. Write $\widehat{\tau}$ as the resulting estimate of τ.
3: Estimate α by solving

$$\frac{1}{n}\sum_{i=1}^{n}(-1)^{1-A_i}c(X_i)q^*(A_i, Z_i, X_i; \widehat{\tau})\exp(-\beta_0(X_i;\alpha)A_i) = 0 \tag{29}$$

Denote the resulting estimate of α as $\widehat{\alpha}$. The estimated conditional vaccine effectiveness is

$$\widehat{VE}(x) = 1 - \exp(\beta_0(x; \widehat{\alpha})).$$

and

$$\tilde{\Omega}(\alpha, \tau) = \left(E\left[\frac{\partial \tilde{G}_i(\alpha, \tau)}{\partial \alpha^T}\right], E\left[\frac{\partial \tilde{G}_i(\alpha, \tau)}{\partial \tau^T}\right]\right).$$

Here $c(X)$ is a user-specified function of X with the sample dimension as α.

Suppose that in Algorithm 2, one specifies $\beta_0(X; \alpha) = X^T\alpha$, then a natural choice for $c(X)$ is $c(X) = X$. A sandwich estimator of the asymptotic variance of $(\widehat{\alpha}, \widehat{\tau})$ can be deduced from previous derivations. Under Assumption 10, we have shown that one can identify $VE(X)$, however, one may be unable to identify the population marginal VE without an additional assumption. Interestingly, we note that the population marginal risk ratio would remain non-identified even if one had access to a random sample from the target population to inform the marginal distribution of X. Specifically, as shown in Huitfeldt et al. (2019), the marginal $RR = E[Y(1)]/E[Y(0)]$ can be written as $RR = E[RR(X)|Y(0) = 1]$, i.e. the average risk ratio among subjects who would contract say Influenza had they possibly contrary to fact, not been vaccinated against Influenza. However, the distribution of X within the group $Y(a = 0) = 1$ cannot be identified in presence of unmeasured confounding, thus ruling out identification of the population marginal RR.

Define q^* as before and

$$\beta_0^*(x) = \log\left(\frac{E[q^*(A, Z, x)I(A = 1, Y = 1)|S = 1, X = x]}{E[q^*(A, Z, x)I(A = 0, Y = 1)|S = 1, X = x]}\right).$$

Denote α_0^* as the value of α such that $\beta_0(x; \alpha_0^*) = \beta_0^*(x)$. Below we give the set of regularity conditions such that $(\widehat{\alpha}, \widehat{\tau})$ is a regular and asymptotically linear estimator of (α_0^*, τ_0^*):

R'.1 The function $\tau \mapsto q(A, Z, X; \tau)$ is Lipschitz in a neighborhood of τ_0^* and $\alpha \mapsto \beta_0(X; \alpha)$ is Lipschitz in a neighborhood of α_0^*.

R'.2 $E[q(A,Z,X;\tau_0^*)^2] < \infty$, $E[m(W,A,X)^2] < \infty$, $E[C(X)^2] < \infty$ and $E[\exp(-2\beta_0(X;\alpha_0^*))] < \infty$. ;

R'.3 The function $\tau \mapsto q(A,Z,X;\tau)$ is differentiable at τ_0^* and $\alpha \mapsto \beta_0(X;\alpha)$ is differentiable at α_0^*. The derivative matrix $\tilde{\Omega}(\alpha_0^*, \tau^*)$ is nonsingular;

R'.4 $\dfrac{1}{n}\sum_{i=1}^n \tilde{G}_i(\widehat{\alpha},\widehat{\tau}) = o_p(n^{-1/2})$ and $(\widehat{\alpha},\widehat{\tau}) \xrightarrow{p} (\alpha_0^*, \tau_0^*)$.

I Simulation settings and results

Table S1: Data generating processes and evaluated methods in the simulation study. We chose various values of η_0 to generate data with a range of infection prevalance in the population; we chose the log risk ratio β_0 to be $\log(0.2)$, $\log(0.5)$, $\log(0.7)$ and 0 as different effect sizes. Here $\text{expit}(x) = \exp(x)/[1 + \exp(x)]$.

Data generating process	Methods
**Binary U, Z, W, No X :**<br>U : Bernoulli(0.5)<br>Z : Bernoulli($0.2 + 0.4U$)<br>A : Bernoulli($0.2 + 0.4U$)<br>Y : Bernoulli($\exp(\eta_0 + \beta_0 A - 0.693U)$)<br>$W$: Bernoulli($0.02 - 0.01U$)<br>D : Bernoulli($0.02 - 0.015U$)<br>S : Bernoulli($\max(Y, D, W) \times (0.1 + 0.4U)$)<br><br>**Continuous U, X, Z, W :**<br>U, X : Uniform(0, 1);<br>A : Bernoulli($\text{expit}(-1 + U + 0.25X)$)<br>$Z$: N($0.25A + 0.25X + 5U, 0.25^2$);<br>$W$: N($0.25X + 2U, 0.25^2$);<br>Y : Bernoulli($\exp(\eta_0 + \beta_0 A - 2U - 0.25X -$ $0.75UX)$)<br>D : Bernoulli($\exp(-4.605 + 0.25X - 0.2U)$)<br>$S$: Bernoulli($\max(Y, D) \times \text{expit}(-1.4 + 0.5X +$ $2U + UX)$).	**NC estimator:**<br>Proposed estimator given by Algorithm 1. In the binary setting, we use a saturated parametric model (15) for the treatment confounding bridge function, with $m(W, A) = (1, W, A, WA)^T$; in the continuous setting, we use model (16) and $m(W, A, X) = (1, W, A, X)^T$. We set $c(X) = 1$ in both settings;<br><br>**NC-Oracle estimator:**<br>The NC estimator that uses the true treatment confounding bridge function $q(A, Z, X)$;<br><br>**Logistic regression:**<br>Logistic regression of Y on A (and X in the continuous setting), overlooking the unmeasured confounders U;<br><br>**IPTW estimator:**<br>The IPTW estimator in Equation (6) but ignoring the unmeasured confounders U (Schnitzer 2022). The propensity score is estimated by logistic regression.

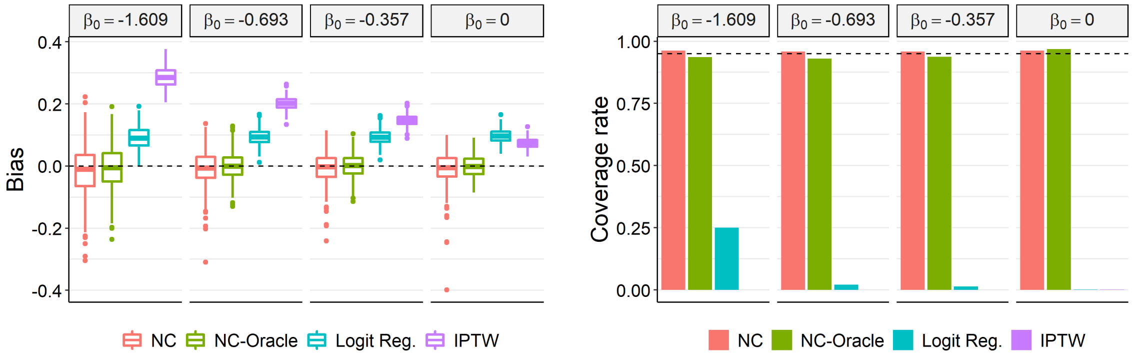

(a)

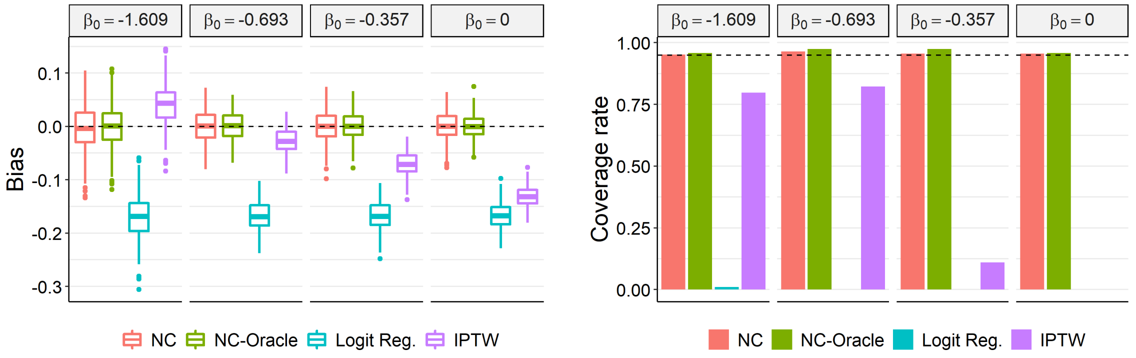

(b)

Figure S1: Bias (left) and coverage rates of 95% confidence interval (right) for the proposed NC estimator, the oracle estimator (NC-Oracle), logistic regression (Logit Reg.) and IPTW estimator with a (a) binary or (b) continuous unmeasured confounder.

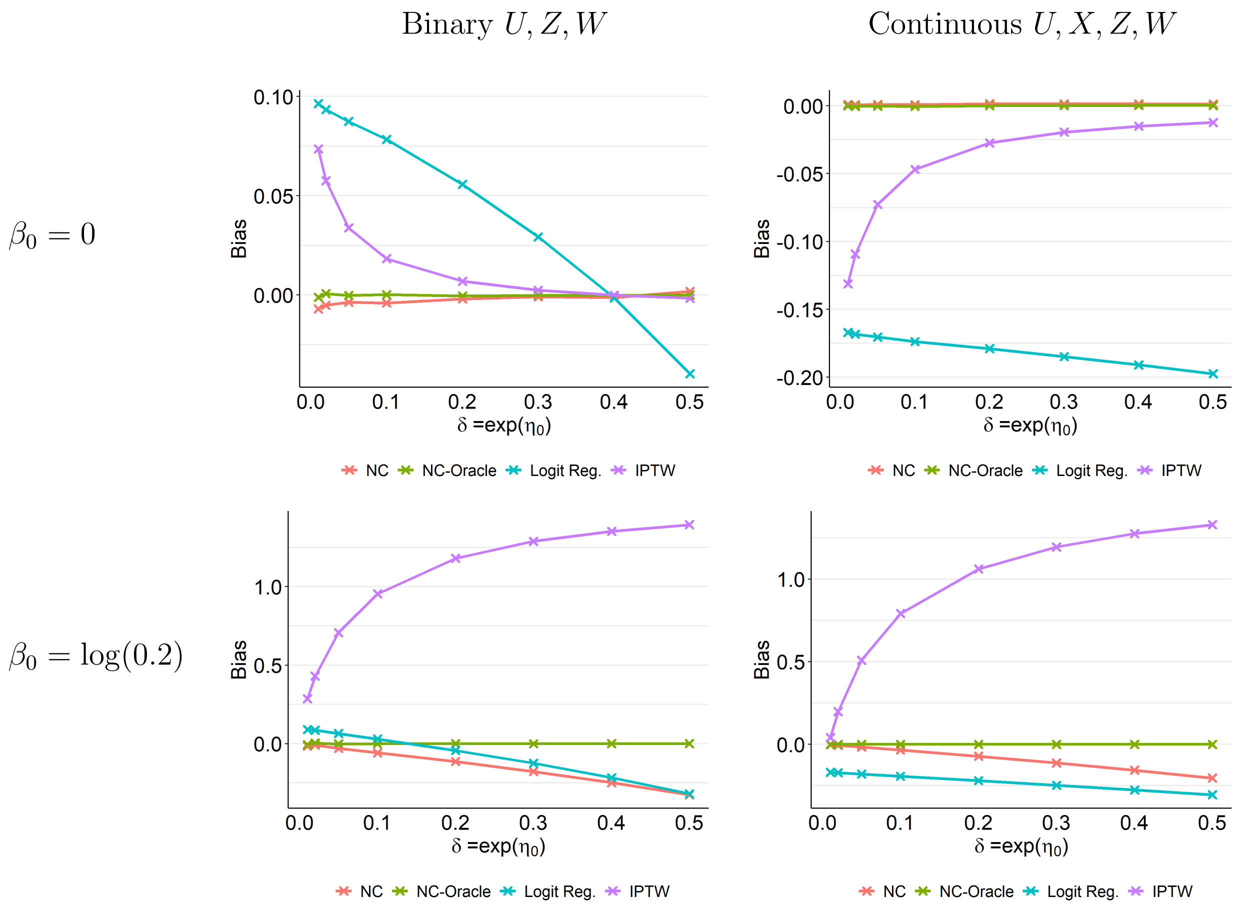

Figure S2: Bias of the proposed NC estimator, the oracle estimator (NC-Oracle), logistic regression (Logit Reg.) and the IPTW estimator over 500 replications in the simulation, with a range of baseline outcome risk $\delta = \exp(\eta_0)$ and log risk ratio β_0.

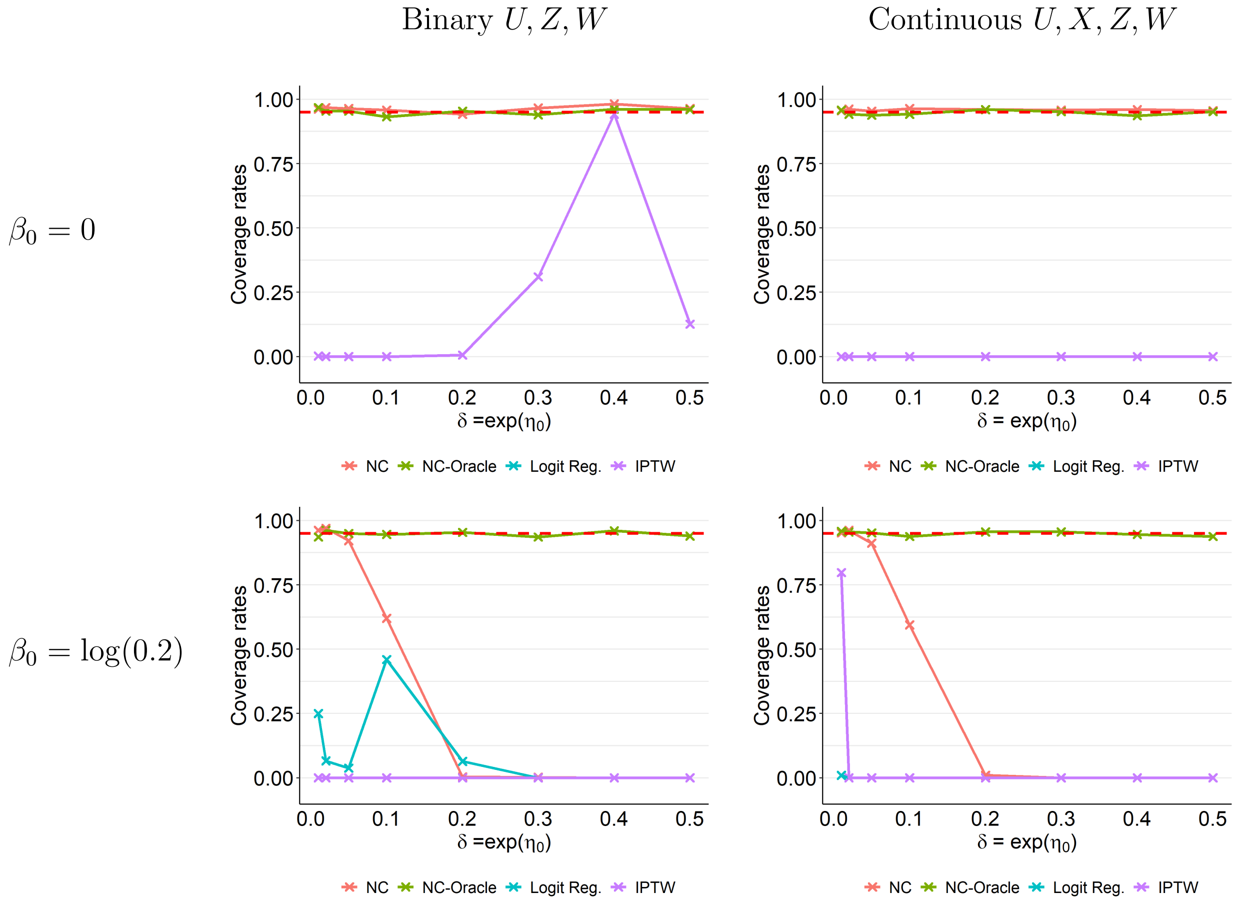

Figure S3: Coverage probability of 95% confidence intervals for the proposed NC estimator, the oracle estimator (NC-Oracle), logistic regression (Logit Reg.) and the IPTW estimator over 500 replications in the simulation, with a range of baseline outcome risk $\delta = \exp(\eta_0)$ and log risk ratio β_0. The curves for logistic regression and IPTW overlap for $\beta_0 = 0$. The red dash line indicates the nominal level 95%.

J Detailed results of University of Michigan Health System Data analysis

Table S2: Descriptive statistics of University of Michigan Health System COVID-19 Data. Variables were summarized as counts (percentage%).

	Unvaccinated (N=12,672)	Vaccinated (N=39,591)
Vaccine types		
Pfizer-BioNTech	/	20,312 (51.3%)
Moderna	/	10,831 (27.4%)
Johnson & Johnson's Janssen	/	1,409 (3.6%)
Other	/	7,039 (17.8%)
SARS-Cov-2 infection	3,074 (24.2%)	2,774 (7.0%)
NCE: Immunization before Dec 2020	3,854 (30.4%)	18,167 (45.9%)
NCO conditions		
Arm/leg cellulitis	39 (0.3%)	161 (0.4%)
Eye/ear disorder	83 (0.6%)	518 (1.3%)
Gastro-esophageal disease	619 (4.9%)	3,188 (8.0%)
Atopic dermatitis	13 (0.1%)	41 (0.1%)
Injuries	1,033 (8.2%)	3,690 (9.3%)
General adult examination	752 (5.9%)	4,687 (11.8%)
No. of NCO conditions ≥ 1	2,258 (17.8%)	10,355 (26.2%)
Age		
≤ 18	1,683 (13.3%)	2,406 (6.1%)
$\geq 18, < 60$	8,667 (68.4%)	23,495 (59.3%)
≥ 60	2,322 (18.3%)	13,690 (34.6%)
Male	5,357 (42.3%)	16,241 (41.0%)
White	9.012 (71.1%)	30,748 (77.7%)
Charlson score ≥ 3	869 (6.8%)	3,230 (8.2%)

Table S3: Logistic regression of SARS-Cov-2 infection on COVID-19 vaccination, the NCE (previous immunization) and other baseline covariates.

	Est.	S.E.	p-value
(Intercept)	-1.30	0.06	<0.001
COVID-19 vaccination	-1.56	0.03	<0.001
Previous Immunization	0.37	0.03	<0.001
Age $\geq$18, $\leq$60	0.29	0.05	<0.001
Age $\geq$60	0.10	0.06	0.099
Male	0.01	0.03	0.750
White	0.32	0.04	<0.001
Charlson score $\geq$3	0.15	0.05	0.005
Calendar month			
April	-0.00	0.05	0.940
May	-0.98	0.06	<0.001
June	-1.79	0.08	<0.001
July	-1.16	0.07	<0.001
August	-0.53	0.05	<0.001
September	-0.40	0.05	<0.001
October	-0.10	0.04	0.026

Table S4: Logistic regression of having at least one NCO conditions on COVID-19 vaccination, the NCE (previous immunization) and other covariates.

	Est.	S.E.	p-value
(Intercept)	-2.32	0.06	<0.001
COVID-19 vaccination	0.28	0.03	<0.001
Previous Immunization	0.63	0.02	<0.001
Age $\geq$18, $\leq$60	0.28	0.04	<0.001
Age $\geq$60	0.76	0.05	<0.001
Male	-0.03	0.02	0.190
White	0.19	0.03	<0.001
Charlson score $\geq$3	0.38	0.04	<0.001
Calendar month			
April	-0.05	0.04	0.290
May	0.16	0.04	<0.001
June	0.16	0.04	<0.001
July	0.16	0.04	<0.001
August	0.14	0.04	<0.001
September	-0.03	0.04	0.360
October	0.04	0.04	0.309

K Extension to other data types

K.1 Polytomous treatment and binary outcome

Let $\mathcal{A} = \{a_0, a_1, \ldots, a_J\}$ denote the set of levels for a polytomous treatment (e.g. different types of COVID-19 vaccines), where a_0 is the reference treatment level (e.g. no vaccination). Let $\mathcal{Y} = \{0, 1\}$ denote the support of the binary outcome of interest. We replace the Assumption 2 with the following homogeneity assumption:

$$P(Y = 1 \mid A = a_j, U, X) = \exp(\beta_j) g(U, X) \qquad \text{for } j = 1, \ldots, J \tag{30}$$

where $g(U, X) = P(Y = 1 \mid A = a_0, U, X)$ is the risk function in the reference treatment group. Equation (30) indicates that the risk ratio of $Y = 1$ in the group $A = a_j$ vs. $A = a_0$ is homogeneous in every (U, X) stratum, where β_j is the logarithm of said risk ratio. Assume that the NCE independence conditions (Assumption 3) still hold, and the definition of the treatment confounding bridge function (Assumption 4) holds for $A = \{a_0, \ldots, a_J\}$, then for an arbitrary one-dimensional function $c(X)$ such that

$$E\{c(X) Y I(A = a_0) q(a_0, Z, X) \mid S = 1\} \neq 0,$$

we have

$$\beta_j = \log\left(\frac{E\{c(X) Y I(A = a_j) q(a_j, Z, X) \mid S = 1\}}{E\{c(X) Y I(A = a_0) q(a_0, Z, X) \mid S = 1\}}\right) \qquad \text{for } j = 1, \ldots, J.$$

Proof. For $j = 0, \ldots, J$, we have

$$\begin{aligned}
& E\left[c(X) Y I(A = a_j) q(a_j, Z, X) \mid S = 1\right] \\
=& E[c(X) I(A = a_j) q(a_j, Z, X) \mid Y = 1, S = 1] P(Y = 1 \mid S = 1) \\
=& E[c(X) E\{I(A = a_j) q(a_j, Z, X) \mid Y = 1, S = 1, U, X\} \mid Y = 1, S = 1] P(Y = 1 \mid S = 1) \\
=& E[c(X) E\{q(a_j, Z, X) \mid A = a_j, Y = 1, S = 1, U, X\} P(A = a_j \mid Y = 1, S = 1, U, X) \mid Y = 1, S = 1] \\
& \quad P(Y = 1 \mid S = 1) \\
\overset{A.1, A.3}{=}& E\left[c(X) E\{q(a_j, Z, X) \mid A = a_j, U, X\} P(A = a_j \mid Y = 1, U, X) \mid Y = 1, S = 1\right] P(Y = 1 \mid S = 1) \\
\overset{A.4}{=}& E\left[\frac{c(X) P(A = a_j \mid Y = 1, U, X)}{P(A = a_j \mid U, X)} \mid Y = 1, S = 1\right] P(Y = 1 \mid S = 1) \\
=& E\left[\frac{c(X) P(Y = 1 \mid A = a_j, U, X)}{P(Y = 1 \mid U, X)} \mid Y = 1, S = 1\right] P(Y = 1 \mid S = 1).
\end{aligned}$$

Therefore, for $j = 1, \ldots, J$ we have

$$
\begin{aligned}
&\frac{E\left[c(X)YI(A=a_j)q(a_j,Z,X) \mid S=1\right]}{E\left[c(X)YI(A=a_0)q(a_0,Z,X) \mid S=1\right]} \\
=&\frac{E\left[\dfrac{c(X)P(Y=1 \mid A=a_j,U,X)}{P(Y=1 \mid U,X)} \mid Y=1,S=1\right]P(Y=1 \mid S=1)}{E\left[\dfrac{c(X)P(Y=1 \mid A=a_0,U,X)}{P(Y=1 \mid U,X)} \mid Y=1,S=1\right]P(Y=1 \mid S=1)} \\
=&\frac{E\left[\dfrac{c(X)P(Y=1 \mid A=a_0,U,X)\exp(\beta_j)}{P(Y=1 \mid U,X)} \mid Y=1,S=1\right]P(Y=1 \mid S=1)}{E\left[\dfrac{c(X)P(Y=1 \mid A=a_0,U,X)}{P(Y=1 \mid U,X)} \mid Y=1,S=1\right]P(Y=1 \mid S=1)} \\
=&\exp(\beta_j).
\end{aligned}
$$

□

Consider an approximation of the treatment confounding bridge function $q^*(a,z,x)$ under the rare disease assumption that satisfies

$$E[q^*(a,Z,X) \mid A=a,W,X] = 1/P(A=a \mid W,X,Y=0,S=1).$$

As before, it is straightforward to show that

$$E\left[(1-Y)\left\{m(A,W,X)q^*(A,Z,X) - \sum_{j=0}^{J} m(a_j,W,X)\right\} \mid S=1\right] = 0 \tag{31}$$

for an arbitrary function $m(A,W,X)$.

With a suitable parametric model $q^*(a,z,x;\tau)$ the nuisance parameter τ can be estimated by solving the estimating equation

$$\sum_{i=1}^{n}(1-Y_i)\left\{m(A_i,W_i,X_i)q^*(A_i,Z_i,X_i;\tau) - \sum_{j=0}^{J} m(a_j,W_i,X_i)\right\} = 0 \tag{32}$$

We can estimate the log risk ratio β_j with Algorithm 3 below:

K.2 Binary treatment and polytomous outcome

Suppose the treatment is binary such that $\mathcal{A} = \{0,1\}$ and $\mathcal{Y} = \{y_0, y_1, \ldots, y_K\}$ is the support of a polytomous outcome, where y_0 denotes a reference outcome. Suppose the treatment effect on each level of the outcome is of interest. We replace Assumption 2 with the following homogeneity assumption:

$$P(Y=y_k \mid A=1,U,X) = \exp(\beta^k)g_k(U,X) \qquad \text{for } k=1,\ldots,K \tag{34}$$

Algorithm 3 Negative control method to estimate risk ratio with a polytomous treatment.

1: Identify the variables in the data according to Figure 1(e), in particular the NCEs and NCOs.

2: Estimate the treatment confounding bridge function by solving Equation (32) with a suitable parametric model $q^*(A, Z, X; \tau)$ and a user-specified function $m(W, A, X)$. Write $\widehat{\tau}$ as the resulting estimate of τ.

3: Estimate β_j by

$$\widehat{\beta}_j = \log\left(\frac{\sum c(X_i)q^*(A_i, Z_i, X_i; \widehat{\tau})I(A_i = a_j)Y_i}{\sum c(X_i)q^*(A_i, Z_i, X_i; \widehat{\tau})I(A_i = a_0)Y_i}\right); \tag{33}$$

for $j = 1, \ldots, J$, where $c(\cdot)$ is a user-specified one-dimensional function.

where $g_k(U, X) = P(Y = y_k \mid A = 0, U, X)$ is the risk function of $Y = y_k$ in the baseline treatment group. Equation (34) indicates that the risk ratio of $Y = y_k$ between $A = 1$ vs. $A = 0$ is homogeneous across every U, X stratum. This also requires that $\sum_{k=1}^{K} \exp(\beta^k)g_k(U, X) < 1$, which is likely to hold under a rare disease assumption that $P(Y \neq y_0 \mid A, U, X)$ is small for every A, U, X. Assume the NCE condition (Assumption 3). Suppose Assumptions 1, 3 and 1 continue to hold, then for an arbitrary one-dimensional function $c(X)$ such that

$$E\{c(X)I(Y = y_k)(1 - A)q(A, Z, X) \mid S = 1\} \neq 0,$$

we have

$$\beta^k = \log\left(\frac{E\{c(X)I(Y = y_k)Aq(A, Z, X) \mid S = 1\}}{E\{c(X)I(Y = y_k)(1 - A)q(A, Z, X) \mid S = 1\}}\right) \qquad \text{for } k = 1, \ldots, K.$$

Proof. For $k = 0, \ldots, K$ and $a = 0, 1$, we have

$$\begin{aligned}
&E\left[c(X)I(Y = y_k)I(A = a)q(a, Z, X) \mid S = 1\right] \\
=&E[c(X)I(A = a)q(a, Z, X) \mid Y = y_k, S = 1]P(Y = y_k \mid S = 1) \\
=&E[c(X)E\{I(A = a)q(a, Z, X) \mid Y = y_k, S = 1, U, X\} \mid Y = y_k, S = 1]P(Y = y_k \mid S = 1) \\
=&E[c(X)E\{q(a, Z, X) \mid A = a, Y = y_k, S = 1, U, X\}\times \\
&\qquad P(A = a \mid Y = y_k, S = 1, U, X) \mid Y = y_k, S = 1]P(Y = y_k \mid S = 1) \\
\overset{A.1,A.3}{=}&E\left[c(X)E\{q(a, Z, X) \mid A = a, U, X\}P(A = a \mid Y = y_k, U, X) \mid Y = y_k, S = 1\right]\times \\
&\qquad P(Y = y_k \mid S = 1) \\
\overset{A.4}{=}&E\left[\frac{c(X)P(A = a \mid Y = y_k, U, X)}{P(A = a \mid U, X)} \mid Y = y_k, S = 1\right]P(Y = y_k \mid S = 1) \\
=&E\left[\frac{c(X)P(Y = y_k \mid A = a, U, X)}{P(Y = y_k \mid U, X)} \mid Y = y_k, S = 1\right]P(Y = y_k \mid S = 1).
\end{aligned}$$

Therefore, for $k = 1, \ldots, K$ we have

$$
\begin{aligned}
&\frac{E\left[c(X)I(Y=y_k)Aq(a_j,Z,X) \mid S=1\right]}{E\left[c(X)I(Y=y_k)(1-A)q(a_0,Z,X) \mid S=1\right]} \\
=&\frac{E\left[\dfrac{c(X)P(Y=y_k \mid A=1,U,X)}{P(Y=y_k \mid U,X)} \mid Y=y_k, S=1\right]P(Y=y_k \mid S=1)}{E\left[\dfrac{c(X)P(Y=y_k \mid A=0,U,X)}{P(Y=y_k \mid U,X)} \mid Y=y_k, S=1\right]P(Y=y_k \mid S=1)} \\
=&\frac{E\left[\dfrac{c(X)P(Y=y_k \mid A=0,U,X)\exp(\beta^k)}{P(Y=y_k \mid U,X)} \mid Y=y_k, S=1\right]P(Y=y_k \mid S=1)}{E\left[\dfrac{c(X)P(Y=y_k \mid A=0,U,X)}{P(Y=y_k \mid U,X)} \mid Y=y_k, S=1\right]P(Y=y_k \mid S=1)} \\
=&\exp(\beta^k).
\end{aligned}
$$

□

Suppose the rare disease assumption 6 is replaced by the rare disease assumption below:

$$P(Y \neq y_0 \mid A, W, U, X) \approx 0 \qquad \text{almost surely,}$$

then we can similarly use the function $q^*(\cdot)$ defined in Assumption 7 as an approximation of $q(\cdot)$. With a suitable parametric model $q^*(a, z, x; \tau)$, the nuisance parameter τ can be estimated by solving the estimating equation

$$\sum_{i=1}^{n} I(Y_i = y_0)\left\{m(A_i, W_i, X_i)q^*(A_i, Z_i, X_i; \tau) - \sum_{a=0}^{1} m(a, W_i, X_i)\right\} = 0 \tag{35}$$

. We can then estimate the log risk ratios β^k with Algorithm 4 below:

Algorithm 4 Negative control method to estimate risk ratio with a polytomous outcome.

1: Identify the variables in the data according to Figure 1(e), in particular the NCEs and NCOs.
2: Estimate the treatment confounding bridge function by solving Equation (**??**) with a suitable parametric model $q^*(A, Z, X; \tau)$ and a user-specified function $m(W, A, X)$. Write $\widehat{\tau}$ as the resulting estimate of τ.
3: Estimate β^k by

$$\widehat{\beta}^k = \log\left(\frac{\sum c(X_i)I(Y_i = y_k)A_i q^*(A_i, Z_i, X_i; \widehat{\tau})}{\sum c(X_i)I(Y_i = y_k)(1 - A_i)q^*(A_i, Z_i, X_i; \widehat{\tau})}\right); \tag{36}$$

for $k = 1, \ldots, K$, where $c(\cdot)$ is a user-specified one-dimensional function.

K.3 Polytomous treatment and outcome

Suppose both the treatment and outcome of interest are polytomous such that $\mathcal{A} = \{a_0, a_1, \ldots, a_J\}$ and $\mathcal{Y} = \{y_0, y_1, \ldots, y_K\}$. Suppose we are interested in the effect of different treatments on each level of the outcome. The results of the previous sections can be extended to identify and estimate the treatment effect. We replace Assumption 2 with the following homogeneity assumption:

$$P(Y = y_k \mid A = a_j, U, X) = \exp(\beta_j^k) g_k(U, X) \qquad \text{for } j = 1, \ldots, J \text{ and } k = 1, \ldots, K.$$

Also assume the following rare disease assumption holds:

$$P(Y \neq y_0 \mid A, W, U, X) \approx 0 \qquad \text{almost surely,}$$

then the function $q^*(\cdot)$ defined in Assumption 7 can serve as an approximation of $q(\cdot)$. With a suitable parametric model $q^*(a, z, x; \tau)$, the nuisance parameter τ can be estimated by solving the estimating equation

$$\sum_{i=1}^{n} I(Y_i = y_0) \left\{ m(A_i, W_i, X_i) q^*(A_i, Z_i, X_i; \tau) - \sum_{j=0}^{J} m(a_j, W_i, X_i) \right\} = 0 \qquad (37)$$

.

then the log risk ratio β_j^k for $Y = y_k$ of $A = a_j$ vs. $A = a_0$ can be estimated with Algorithm 5

Algorithm 5 Negative control method to estimate risk ratio with a polytomous treatment and outcome.

1: Identify the variables in the data according to Figure 1(e), in particular the NCEs and NCOs.

2: Estimate the treatment confounding bridge function by solving Equation (14) with a suitable parametric model $q^*(A, Z, X; \tau)$ and a user-specified function $m(W, A, X)$. Write $\widehat{\tau}$ as the resulting estimate of τ.

3: Estimate β_j^k by

$$\widehat{\beta}_j^k = \log \left(\frac{\sum c(X_i) I(Y_i = y_k, A_i = a_j) q^*(A_i, Z_i, X_i; \widehat{\tau})}{\sum c(X_i) I(Y_i = y_k, A_i = a_0) q^*(A_i, Z_i, X_i; \widehat{\tau})} \right); \qquad (38)$$

for $k = 1, \ldots, K$, where $c(\cdot)$ is a user-specified one-dimensional function.

L Approximate equivalent of RR and OR for a rare infection

By Assumption 2, the conditional risk ratio is

$$\text{RR} = \exp(\beta_0) = \frac{P(Y = 1 \mid A = 1, U, X)}{P(Y = 1 \mid A = 0, U, X)}.$$

Consider the conditional odds ratio

$$\text{OR} := \frac{P(Y = 1 \mid A = 1, U, X)P(Y = 0 \mid A = 0, U, X)}{P(Y = 1 \mid A = 0, U, X)P(Y = 0 \mid A = 1, U, X)} = \text{RR} \times \frac{P(Y = 0 \mid A = 0, U, X)}{P(Y = 0 \mid A = 1, U, X)}.$$

Under the rare infection Assumption 6, we have

$$1 - \delta \leq P(Y = 0 \mid A = a, U, X) \leq 1, \qquad a = 0, 1$$

for a small $\delta > 0$.

Therefore, we have

$$1 - \delta \leq \frac{\text{OR}}{\text{RR}} = \frac{P(Y = 0 \mid A = 0, U, X)}{P(Y = 0 \mid A = 1, U, X)} \leq \frac{1}{1 - \delta}.$$

If $\delta \approx 0$, then OR $\approx$ RR.
