# Supplementary material for: Double Negative Control Inference in Test-Negative Design Studies of Vaccine Effectiveness
Source: ArXiv. 2022 Mar 23:arXiv:2203.12509v2. Preprint. [Version 2] (PMC8963685)
Supplement: Supplementary file 1 [file NIHPP2203.12509V2-supplement-1.pdf]

A Proof of Proposition 1

The result immediately follows if we can show that

$$E\left[(-1)^{1-A}\frac{1}{P(A|U,X)}\exp(-\beta_0 A)\middle|U,X,Y=1,S=1\right]=0. \tag{20}$$

By Assumption 2, the left-hand side of (20) equals

$$E\left[(-1)^{1-A}\frac{1}{P(A|U,X)}\exp(-\beta_0 A)\middle|U,X,Y=1\right].$$

We further have

$$\begin{aligned}
&E\left[(-1)^{1-A}\frac{1}{P(A|U,X)}\exp(-\beta_0 A)\middle|U,X,Y=1\right]\\
=&\sum_{a=0}^{1}(-1)^{1-a}\frac{1}{P(A=a|U,X)}\exp(-\beta_0 a)P(A=a|U,X,Y=1)\\
=&\sum_{a=0}^{1}(-1)^{1-a}\frac{1}{P(A=a|U,X)}\exp(-\beta_0 a)\frac{P(A=a|U,X)P(Y=1|A=a,U,X)}{P(Y=1|U,X)}\\
\overset{A.3}{=}&\sum_{a=0}^{1}(-1)^{1-a}\exp(-\beta_0 a)\frac{\exp(\beta_0 a)P(Y=1|A=0,U,X)}{P(Y=1|U,X)}\\
=&\sum_{a=0}^{1}(-1)^{1-a}\frac{P(Y=1|A=0,U,X)}{P(Y=1|U,X)}\\
=&0.
\end{aligned}$$

B Existence of solutions to (6)

In this section, we provide the conditions of existence of solutions to (6). The conditions for (11) can be similarly derived. The results in this section directly adapted from Appendix B of Cui et al. (2020).

Let $L^2\{F(t)\}$ denote the Hilbert space of all square-integrable functions of t with respect to distribution function $F(t)$, equiped with inner product $\langle g_1, g_2\rangle = \int g_1(t)g_2(t)dF(t)$. Let $T_{a,x}$ denote the operator $L^2\{F(z|a,x)\} \to L^2\{F(u|a,x)\}$, $T_{a,x}q = E[q(Z)|A = a, U = u, X = x]$ and let $(\lambda_{a,x,n}, \varphi_{a,x,n}, \phi_{a,x,n})$ denote a singular value decomposition of $T_{a,x}$. The solution to (6) exists if:

(1) $\int\int f(z|a,u,x)f(u|a,z,x)dzdu < \infty$;

(2) $\int P^{-2}(A = a|U = u, X = x)f(u|a,x)du < \infty$;

(3) $\sum_{n=1}^{\infty} \lambda_{a,x,n}^{-2} |\langle P^{-1}(A = a|U = u, X = x), \phi_{a,x,n}\rangle|^2 < \infty$.

C Proof of Theorem 1

It suffices to show that

$$E[(-1)^{1-A}q(A,Z,X)\exp(-\beta_0 A)|U,X,Y=1,S=1]=0.$$

By Assumption 2, the left-hand side is

$$\begin{aligned}
&E[e^{-\beta_0 A}(-1)^{1-A}q(A,Z,X)|U,X,Y=1] \\
=&E\{e^{-\beta_0 A}(-1)^{1-A}E[q(A,Z,X)|A,U,X,Y=1]|U,X,Y=1\} \\
\overset{A.4}{=}&E\{e^{-\beta_0 A}(-1)^{1-A}E[q(A,Z,X)|A,U,X]|U,X,Y=1\} \\
\overset{A.5}{=}&E\{e^{-\beta_0 A}(-1)^{1-A}\frac{1}{P(A|U,X)}|U,X,Y=1\} \\
=&0
\end{aligned}$$

The last equality is proved in Appendix A.

D Treatmeng bridge function and estimation with categorical NCE, NCO and unmeasured confounders.

We consider a categorical unmeasured confounder U with categories $u_1, \ldots, u_J$ and NCE Z with categories $z_1, \ldots, z_I$. We assume there are no other covariates X. We write $p_{ia.k} = P(Z = z_i, A = a | U = u_j)$ for $i = 1, \ldots, I$, $a = 0, 1$, and $j = 1, \ldots, J$.

Similar to before, the treatment confounding bridge function $q(a, z)$ should satisfy

$$\sum_{i=1}^{I} p_{ia.j} q(a, z_i) = 1 \tag{21}$$

for all i, j. Therefore, any solution of the following equation system, if exists, is a treatment confounding bridge function:

$$\begin{aligned}
p_{1a.1} q(a, z_1) + p_{2a.1} q(a, z_2) + \cdots + p_{Ia.1} q(a, z_I) &= 1 \\
p_{1a.2} q(a, z_1) + p_{2a.2} q(a, z_2) + \cdots + p_{Ia.2} q(a, z_I) &= 1 \\
&\cdots \\
p_{1a.J} q(a, z_1) + p_{2a.J} q(a, z_2) + \cdots + p_{Ia.J} q(a, z_I) &= 1
\end{aligned} \tag{22}$$

for $a = 0, 1$. We denote the probability matrix

$$P_{Z,A|U} = \begin{pmatrix} p_{1a.1} & p_{2a.1} & \cdots & p_{Ia.1} \\ p_{1a.2} & p_{2a.2} & \cdots & p_{Ia.2} \\ \vdots & & & \vdots \\ p_{1a.J} & p_{2a.J} & \cdots & p_{Ia.J} \end{pmatrix}. \tag{23}$$

A treatment confounding bridge function exists if the matrix $P_{Z,A|U}$ is invertible.

Suppose besides a categorical NCE Z with levels $z_1, \ldots, z_I$, we also have a categorical

NCO W with levels $w_1, \ldots, w_K$. The integral equation (11) is equivalent to the linear equation system

$$\begin{aligned} p'_{1a.1}q^*(a, z_1) + p'_{2a.1}q^*(a, z_2) + \cdots + p'_{Ia.1}q^*(a, z_I) &= 1 \\ p'_{1a.2}q^*(a, z_1) + p'_{2a.2}q^*(a, z_2) + \cdots + p'_{Ia.2}q^*(a, z_I) &= 1 \\ &\cdots \\ p'_{1a.K}q^*(a, z_1) + p'_{2a.K}q^*(a, z_2) + \cdots + p'_{Ia.K}q^*(a, z_I) &= 1 \end{aligned} \tag{24}$$

for $a = 0, 1$, where $p'_{ia.k} = P(Z = z_i, A = a | W = w_k, Y = 0, S = 1)$. We denote that matrix

$$P'(a) = \begin{pmatrix} p'_{1a.1} & p'_{2a.1} & \cdots & p'_{Ia.1} \\ p'_{1a.2} & p'_{2a.2} & \cdots & p'_{Ia.2} \\ \vdots & & & \vdots \\ p'_{1a.K} & p'_{2a.K} & \cdots & p'_{Ia.K} \end{pmatrix}. \tag{25}$$

Then Assumption 8 is equivalent to the condition that $P'(a)$ is invertible with $I = K$ for $a = 0, 1$, in which case (11) has a unique solution

$$(q^*(a, z_1), \ldots, q^*(a, z_I))^T = [P'(a)]^{-1}\mathbf{1}_I.$$

The probabilities $p'_{ia.k}$'s can all be estimated from the study data.

E Derivation of the treatment confounding bridge function in Example 2

By Assumption 5, the treatment confounding bridge function $q(A, Z, X)$ should satisfy

$$E[q(A, Z, X)|U = u, A = a, X = x] = \frac{1}{P(A = a|U = u, X = x)}$$

for all a, u and X.

We write $q(A, Z, X) = 1 + r(Z, A, X)$, then

$$E[r(Z, a, X)|U, A = a, X] = \frac{1 - P(A = a|U, X)}{P(A = a|U, X)}.$$

Consider

$$r(Z, A, X) = \exp((-1)^A(\tau_0 + \tau_1 A + \tau_2 Z + \tau_3 X)),$$

then

$$\begin{aligned}
&\frac{P(A = 1|U = u, X = x)}{1 - P(A = 1|U = u, X = x)} \\
=& \int r(z, 0, x) f(z|u, x, A = 0) dz \\
=& \int \exp(\tau_0 + \tau_2 z + \tau_3 x) \frac{1}{\sqrt{2\pi\sigma_z^2}} \exp\left(-\frac{(z - \mu_{0z} - \mu_{UZ} u - \mu_{XZ} x)^2}{2\sigma_z^2}\right) dz \\
=& \exp(\tau_0 + \tau_3 x) \exp\left(\tau_2(\mu_{0Z} + \mu_{UZ} u + \mu_{XZ} x) + \frac{\sigma_z^2 \tau_2^2}{2}\right) \\
=& \exp(\tau_0 + \tau_2 \mu_{0Z} + \frac{\sigma_z^2 \tau_2^2}{2} + \tau_2 \mu_{UZ} u + (\tau_3 + \mu_{XZ} \tau_2) x)
\end{aligned}$$

and

$$
\begin{aligned}
&\frac{1-P(A=1|U=u,X=x)}{P(A=1|U=u,X=x)}\\
=&\int r(z,1,x)f(z|u,x,A=1)dz\\
=&\int \exp(-\tau_0-\tau_1-\tau_2 z-\tau_3 x)\frac{1}{\sqrt{2\pi\sigma_z^2}}\exp\left(-\frac{(z-\mu_{0Z}-\mu_{AZ}-\mu_{UZ}u-\mu_{XZ}x)^2}{2\sigma_z^2}\right)dz\\
=&\exp(-\tau_0-\tau_1-\tau_3 x)\exp\left(-\tau_2(\mu_{0Z}+\mu_{AZ}+\mu_{UZ}u+\mu_{XZ}x)+\frac{\sigma_z^2\tau_2^2}{2}\right)\\
=&\exp(-\tau_0-\tau_1-\tau_2\mu_{0Z}-\tau_2\mu_{AZ}+\frac{\sigma_z^2\tau_2^2}{2}-\tau_2\mu_{UZ}u-(\tau_3+\tau_2\mu_{XZ})x)
\end{aligned}
$$

This requires that

$$\tau_1+\tau_2\mu_{AZ}=\sigma_z^2\tau_2^2$$

Because

$$P(A=1|U,X)=\text{expit}(\mu_{0A}+\mu_{UA}U+\mu_{XA}X),$$

we conclude the parameters in the bridge function are

$$\tau_2=\mu_{UA}/\mu_{UZ},$$

$$\tau_3=\mu_{XA}-\mu_{XZ}\tau_2=\mu_{XA}-\mu_{XZ}\mu_{UA}/\mu_{UZ}$$

$$\tau_1=\sigma_z^2\tau_2^2-\tau_2\mu_{AZ}=\frac{\sigma_z^2\mu_{UA}^2}{\mu_{UZ}^2}-\frac{\mu_{UA}\mu_{AZ}}{\mu_{UZ}}$$

$$\tau_0=\mu_{0A}-\tau_2\mu_{0Z}-\frac{\sigma_z^2\alpha_2^2}{2}=\mu_{0A}-\frac{\mu_{UA}\mu_{0Z}}{\mu_{UZ}}-\frac{\sigma_z^2\mu_{UA}^2}{2\mu_{UZ}^2}$$

F Proof of Theorem 2 and Corollary 1

We first introduce a few properties due to the rare disease assumption:

Lemma 1. *Under Assumptions 2, 4, 6 and 7, for every a, w, u and x, we have*

(a)

$$P(Y=1|W=w,U=u,X=x)<\delta, \qquad P(Y=1|A=a,U=u,X=x)<\delta,$$
$$P(Y=1|U=u,X=x)<\delta.$$

(b)

$$1-\delta<\frac{P(A=a|U=u,X=x,Y=0,S=1)}{P(A=a|U=u,X=x)}<\frac{1}{1-\delta}$$

(c)

$$(1-\delta)^2<\frac{P(A=a|W=w,U=u,X=x,Y=0,S=1)}{P(A=a|U=u,X=x,Y=0,S=1)}<\frac{1}{(1-\delta)^2}.$$

(d)

$$f(z|A=a,U=u,X=x)=f(z|W=w,A=a,U=u,X=x,Y=0,S=1).$$

Proof. (a) For every a, w, u and x, we have

$$\begin{aligned}
&P(Y=1|W=w,U=u,X=x)\\
&=\sum_a P(Y=1|A=a,W=w,U=u,X=x)P(A=a|W=w,U=u,X=x)\\
&\overset{A.7}{<}\delta\sum_a P(A=a|W=w,U=u,X=x)\\
&=\delta.
\end{aligned}$$

The rest follows similarly.

(b) For every a, u and x, we have

$$P(A=a|U=u,X=x,Y=0)=P(A=a|U=u,X=x)\times\frac{P(Y=0|A=a,U=u,X=x)}{P(Y=0|U=u,X=x)}.$$

By Lemma 1(a), we have

$$1-\delta<\frac{P(Y=0|A=a,U=u,X=x)}{P(Y=0|U=u,X=x)}=\frac{P(A=a|U=u,X=x,Y=0)}{P(A=a|U=u,X=x)}<\frac{1}{1-\delta}.$$

The result follows by noticing that $P(A=a|U=u,X=x,Y=0)=P(A=a|U=u,X=x,Y=0,S=1)$ due to Assumption 2.

(c) For every a, w, u and x, we have

$$\begin{aligned}
&P(A=a|W=w,U=u,X=x,Y=0)\\
&=P(A=a|W=w,U=u,X=x)\times\frac{P(Y=0|A=a,W=w,U=u,X=x)}{P(Y=0|W=w,U=u,X=x)}\\
&\overset{A.6(a)}{=}P(A=a|U=u,X=x)\times\frac{P(Y=0|A=a,W=w,U=u,X=x)}{P(Y=0|W=w,U=u,X=x)}\\
&=P(A=a|U=u,X=x,Y=0)\times\frac{P(Y=0|U=u,X=x)}{P(Y=0|A=a,U=u,X=x)}\\
&\quad\times\frac{P(Y=0|A=a,W=w,U=u,X=x)}{P(Y=0|W=w,U=u,X=x)}
\end{aligned}$$

By Lemma 1(a), we have

$$1-\delta<\frac{P(Y=0|U=u,X=x)}{P(Y=0|A=a,U=u,X=x)}<\frac{1}{1-\delta}$$

and

$$1-\delta < \frac{P(Y=0|A=a,W=w,U=u,X=x)}{P(Y=0|W=w,U=u,X=x)} < \frac{1}{1-\delta}.$$

We therefore have

$$(1-\delta)^2 < \frac{P(A=a|W=w,U=u,X=x,Y=0)}{P(A=a|U=u,X=x,Y=0)} < \frac{1}{(1-\delta)^2}.$$

Finally, by Assumptions 2 and 6(c), we have

$$P(A=a|U=u,X=x,Y=0) = P(A=a|U=u,X=x,Y=0,S=1),$$

$$P(A=a|W=w,U=u,X=x,Y=0) = P(A=a|W=w,U=u,X=x,Y=0,S=1).$$

We conclude that

$$(1-\delta)^2 < \frac{P(A=a|W=w,U=u,X=x,Y=0,S=1)}{P(A=a|U=u,X=x,Y=0,S=1)} < \frac{1}{(1-\delta)^2}.$$

(d)

$$\begin{aligned} & f(z|W=w,A=a,U=u,X=x,Y=0,S=1) \\ \overset{A.6(c)}{=} & f(z|W=w,A=a,U=u,X=x,Y=0) \\ \overset{A.6(b)}{=} & f(z|A=a,U=u,X=x) \end{aligned}$$

□

Therefore, we have

$$
\begin{aligned}
& E\{q(a,Z,X)|A=a,W,X,Y=0,S=1\} \\
=& \int q(a,z,X)f(z|A=a,W,X,Y=0,S=1)\,\mathrm{d}z \\
=& \int\int q(a,z,X)f(z|A=a,W,U=u,X,Y=0,S=1)f(u|A=a,W,X,Y=0,S=1)\,\mathrm{d}z\,\mathrm{d}u \\
\overset{L.1(d)}{=}& \int\left\{\int q(a,z,X)f(z|A=a,U=u,X)\,\mathrm{d}u\right\}f(u|A=a,W,X,Y=0,S=1)\,\mathrm{d}z \\
\overset{A.5}{=}& \int \frac{1}{P(A=a|U=u,X)}f(u|A=a,W,X,Y=0,S=1)du \\
\overset{L.1(b)}{<}& \frac{1}{1-\delta}\int \frac{1}{P(A=a|U=u,X,Y=0,S=1)}f(u|A=a,W,X,Y=0,S=1)du \\
\overset{L.1(c)}{<}& \frac{1}{(1-\delta)^3}\int \frac{1}{P(A=a|W,U=u,X,Y=0,S=1)}f(u|A=a,W,X,Y=0,S=1)du \\
=& \frac{1}{(1-\delta)^3}\int \frac{f(u|W,X,Y=0,S=1)f(u|A=a,W,X,Y=0,S=1)}{P(A=a|W,X,Y=0,S=1)f(u|A=a,W,X,Y=0,S=1)}\,\mathrm{d}u \\
=& \frac{1}{(1-\delta)^3}\frac{1}{P(A=a|W,X,Y=0,S=1)}\int f(u|W,X,Y=0,S=1)\,\mathrm{d}u \\
=& \frac{1}{(1-\delta)^3}\frac{1}{P(A=a|W,X,Y=0,S=1)}
\end{aligned}
$$

and

$$
\begin{aligned}
&E\{q(a,Z,X)|A=a,W,X,Y=0,S=1\}\\
=&\int \frac{1}{P(A=a|U=u,X)} f(u|A=a,W,X,Y=0,S=1)\,\mathrm{d}u\\
\overset{L.1(b)}{>}&(1-\delta)\int \frac{1}{P(A=a|U=u,X,Y=0,S=1)} f(u|A=a,W,X,Y=0,S=1)\,\mathrm{d}u\\
\overset{L.1(c)}{>}&(1-\delta)^3\int \frac{1}{P(A=a|W,U=u,X,Y=0,S=1)} f(u|A=a,W,X,Y=0,S=1)\,\mathrm{d}u\\
=&(1-\delta)^3\int \frac{f(u|W,X,Y=0,S=1)f(u|A=a,W,X,Y=0,S=1)}{P(A=a|W,X,Y=0,S=1)f(u|A=a,W,X,Y=0,S=1)}\,\mathrm{d}u\\
=&(1-\delta)^3\frac{1}{P(A=a|W,X,Y=0,S=1)}\int f(u|W,X,Y=0,S=1)\,\mathrm{d}u\\
=&(1-\delta)^3\frac{1}{P(A=a|W,X,Y=0,S=1)}.
\end{aligned}
$$

To prove Corollary 1, we have

$$
\begin{aligned}
&E\{q(a,Z,X)|A=a,W,X,Y=0,S=1\}\\
=&\int \frac{1}{P(A=a|U=u,X)} f(u|A=a,W,X,Y=0,S=1)\,\mathrm{d}u\\
=&\int \frac{P(A=a|U=u,X,Y=0,W,S=1)}{P(A=a|U=u,X)P(A=a|W,X,Y=0,S=1)} f(u|W,X,Y=0,S=1)\,\mathrm{d}u\\
\overset{A.6}{=}&\int \frac{P(A=a|U=u,X,Y=0,W)}{P(A=a|U=u,X)P(A=a|W,X,Y=0,S=1)} f(u|W,X,Y=0,S=1)\,\mathrm{d}u
\end{aligned}
$$

If $A \perp\!\!\!\perp Y|U,X$, then together with Assumption 6 we have $P(A=a|U=u,X,Y=0,W) = P(A=a|U=u,X)$, whereby the above equals

$$
\frac{1}{P(A=a|W,X,Y=0,S=1)}\int f(u|W,X,Y=0,S=1)du = \frac{1}{P(A=a|W,X,Y=0,S=1)}.
$$

G Discussion of Assumption 8

Similar to (6), Equation (11) defines a Fredholm integral equation of the first kind, yet only involves observed data. Although a treatment confounding bridge function $q(A, Z, X)$ must satisfy (11), there is no guarantee that solving (11) gives a treatment confounding bridge function if multiple solutions exist. When a solution to (11) exists, we give the following assumptions that guarantee the uniqueness of solution.

Assumption 10 (Completeness)**.**

(a) For any square-integrable function g, if $E[g(Z)|A, U, X] = 0$ almost surely, then $g(Z) = 0$ almost surely;

(b) For any square-integrable function h, if $E[h(U)|A, W, X, Y = 0, S = 1]$ almost surely, then $h(U) = 0$ almost surely.

Intuitively, Assumption 10 is a statement on the information contained in the unmeasured confounders vs. in the negative control variables – Assumption 10(a) requires the confounders U are informative enough about Z in the target population and Assumption 10(b) requires the NCO W is informative enough about U in the subgroup $Y = 0, S = 1$, so that no information is lost when taking the two conditional expectations. Completeness conditions similar to Assumption 10 were originally introduced by Lehmann and Scheffe to identify the so-called unbiase minimum risk estimator (Lehmann and Scheffé, 2012a,b). In econometrics and causal inference literature, completeness conditions have been employed to achieve identifiability for a variety of nonparametric or semiparametric models, such as instrumental variable regression (D'Haultfoeuille, 2011; Newey and Powell, 2003), measurement error models (Hu and Schennach, 2008), synthetic control (Shi et al., 2021), and previous works in negative control methods (Cui et al., 2020; Miao, Shi, and Tchetgen

Tchetgen, 2018; Ying et al., 2021). The completeness condition holds for a wide range of distributions. Newey and Powell (2003) and D'Haultfoeuille (2011) provided justifications in exponential families and discrete distributions with finite support. Andrews (2011) constructed a broad class of bivariate distributions that satisfy the completeness condition.

Assumption 10 have several immediate consequences:

Proposition 2. *(a) Under Assumptions 4, 6 and 10, for any square integrable function g such that $E[g(Z)|A, W, X, S = 0, Y = 1] = 0$ almost surely, then $g(Z) = 0$ almost surely.*

(b) Under Assumptions 10(a) and 1-5, the treatment confounding bridge function is unique. That is, if two square-integrable functions $q(A, Z, X)$ and $q_1(A, Z, X)$ satisfy

$$E[q(a, Z, x)|A = a, U = u, X = x] = E[q_1(a, Z, x)|A = a, U = u, X = x]$$
$$= \frac{1}{P(A = a|U = u, X = x)}$$

for all a, u, x almost surely, then $q(A, Z, X) = q_1(A, Z, X)$ almost surely.

(c) Under Assumptions 4, 6, 8 and 10, Equation (11) has a unique solution $q^*(A, Z, X)$.

We prove Proposition 2 below. Proposition 2 states that the completeness conditions in Assumption 10 and the definitions of the negative control variables lead to a third completeness condition that only involves the study data. Proposition 2 states the uniqueness of the treatment confounding bridge function, $q(A, Z, X)$, and the solution to (11), $q^*(A, Z, X)$. The function $q^*(A, Z, X)$ can therefore be identified from the study data alone and is a good approximation of $q(A, Z, X)$ by Theorem 2.

Proof. (a) Suppose a square-integrable function $g(Z)$ satisfies

$$E[g(Z)|A, W, X, Y = 0, S = 1] = 0 \quad \text{almost surely.}$$

The left-hand side equals

$$\begin{aligned}
&E\{E[g(Z)|U, A, W, X, Y = 0, S = 1]\,|A, W, X, Y = 0, S = 1\} \\
\overset{A.6}{=}&E\{E[g(Z)|U, A, X, Y = 0, S = 1]\,|A, W, X, Y = 0, S = 1\} \\
\overset{A.4}{=}&E\{E[g(Z)|U, A, X]\,|A, W, X, Y = 0, S = 1\}
\end{aligned}$$

By Assumption 10(b), $E[g(Z)|U, A, X] = 0$ almost surely. Then by Assumption 10(a), $g(Z) = 0$ almost surely.

(b) We have

$$E[q(A, Z, X) - q_1(A, Z, X)|A, U, X] = 0$$

almost surely. By Assumtion 10(a), we have

$$q(A, Z, X) - q_1(A, Z, X) = 0$$

almost surely, or $q(A, Z, X) = q_1(A, Z, X)$ almost surely.

(c) If two square-integrable functions $q^*(A, Z, X) = q_1^*(A, Z, X)$ satisfies

$$\begin{aligned}
&E[q^*(A, Z, X)|A, W, X, Y = 0, S = 1] = E[q_1^*(A, Z, X)|A, W, X, Y = 0, S = 1] \\
=&\frac{1}{P(A|W, X, Y = 0, S = 1)}
\end{aligned}$$

almost surely, then

$$E\{[q^*(A,Z,X) - q_1^*(A,Z,X)]|A,W,X,Y=0,S=1\} = 0.$$

Under Assumptions 4, 6 and 10, Proposition 2(a) holds and therefore

$$q^*(A,Z,X) = q_1^*(A,Z,X)$$

almost surely.

□

H Proof of Corollary 2 and further discussion

We first prove equation (12)

$$
\begin{aligned}
&E[m(W,A,X)q^*(A,Z,X) - m(W,1,X) - m(W,0,X)|Y=0,S=1] \\
=&E\left\{m(W,A,X)E\left[q^*(A,Z,X)|W,A,X,Y=0,S=1\right] - m(W,1,X) - m(W,0,X)|Y=0,S=1\right\} \\
\overset{(11)}{=}&E\left\{m(W,A,X)\frac{1}{P(A|W,X,Y=0,S=1)} - m(W,1,X) - m(W,0,X)|Y=0,S=1\right\} \\
=&E\Big\{E\left[m(W,A,X)\frac{1}{P(A|W,X,Y=0,S=1)}|W,X,Y=0,S=1\right] - \\
&\quad m(W,1,X) - m(W,0,X)|Y=0,S=1\Big\} \\
=&E\left\{m(W,1,X) + m(W,0,X) - m(W,1,X) - m(W,0,X)|Y=0,S=1\right\} \\
=&\ 0
\end{aligned}
$$

In fact, one can show that any regular and asymptotically normal estimator of τ that satisfies (11) has influence function of the form

$$
\begin{aligned}
IF(W,Z,A,X) = -\left\{E\left[\frac{\partial q(A,Z,X;\tau)}{\partial \tau}\bigg|_{\tau=\tau_0} m(W,A,X)(1-Y)\bigg|S=1\right]\right\}^{-1}(1-Y) \\
\Big(m(W,A,X)q(A,Z,X) - m(W,1,X) - m(W,0,X)\Big)
\end{aligned}
$$

for an arbitrary function $m(W,A,X)$. Therefore, any regular and asymptotically normal estimator of τ corresponds to the solution of the estimating equation (13) for some function $m(W,A,X)$.

To prove this result, we see that for any parametric submodel that satisfies (11) and is

indexed by s such that the true distribution corresponds to $s = 0$, we have

$$E_s\left\{(1-Y)\left[q(A,Z,X;\tau_s) - \frac{1}{f_s(A|W,X,Y=0,S=1)}\right]m(W,A,X)\middle|S=1\right\} = 0$$

and thus

$$\partial E_s\left\{(1-Y)\left[q(A,Z,X;\tau_s) - \frac{1}{f_s(A|W,X,Y=0,S=1)}\right]m(W,A,X)\middle|S=1\right\}/\partial s\bigg|_{s=0} = 0.$$

Note that

$$\begin{aligned}
&\frac{\partial}{\partial s}E_s[(1-Y)q(A,Z,X;\tau_s)m(W,A,X)|S=1]\\
=&E\left[\frac{\partial q(A,Z,X;\tau)}{\partial \tau}\bigg|_{\tau=\tau_0} m(W,A,X)(1-Y)\middle|S=1\right]\frac{\partial \tau_s}{\partial s}\bigg|_{s=0}+\\
&E[(1-Y)q(A,Z,X)m(W,A,X)S(O|S=1)|S=1]
\end{aligned}$$

and

$$
\begin{aligned}
&\frac{\partial}{\partial s}E_s\Bigg[\frac{(1-Y)m(W,A,X)}{f_s(A|W,X,Y=0,S=1)}\Bigg|S=1\Bigg]\\
=&E\Bigg[-(1-Y)m(W,A,X)\frac{\frac{\partial}{\partial s}f_s(A|W,X,Y=0,S=1)}{f^2(A|W,X,Y=0,S=1)}\Bigg|S=1\Bigg]+\\
&\quad E\Bigg[\frac{m(W,A,X)(1-Y)}{f(A|W,X,Y=0,S=1)}S(W,A,X,Y=0|S=1)\Bigg|S=1\Bigg]\\
=&E\Bigg[-\frac{m(W,A,X)(1-Y)}{f(A|W,X,Y=0,S=1)}S(A|W,X,Y=0,S=1)\Bigg|S=1\Bigg]+\\
&\quad E\Bigg[\frac{m(W,A,X)(1-Y)}{f(A|W,X,Y=0,S=1)}S(W,A,X,Y=0|S=1)\Bigg|S=1\Bigg]\\
=&E\Bigg[\frac{m(W,A,X)(1-Y)}{f(A|W,X,Y=0,S=1)}S(W,X,Y=0|S=1)\Bigg|S=1\Bigg]\\
=&E\Bigg[(1-Y)\{m(W,1,X)-m(W,0,X)\}S(W,X,Y=0|S=1)\Bigg|S=1\Bigg]\\
=&E\Bigg[(1-Y)\{m(W,1,X)-m(W,0,X)\}S(O|S=1)\Bigg|S=1\Bigg]
\end{aligned}
$$

Rearranging the terms, we have

$$
\begin{aligned}
&\frac{\partial\tau_s}{\partial s}\Bigg|_{s=0}=\\
&E\Bigg[-\Bigg\{E\Bigg[\frac{\partial q(A,Z,X;\tau)}{\partial\tau}\Bigg|_{\tau=\tau_0}m(W,A,X)(1-Y)\Bigg|S=1\Bigg]\Bigg\}^{-1}(1-Y)\\
&\qquad\Big(m(W,A,X)q(A,Z,X)-m(W,1,X)-m(W,0,X)\Big)S(O|S=1)\Bigg|S=1\Bigg]
\end{aligned}
$$

I Discussion on the rare disease assumption 7

Through this section, we set $c(X) = 1$.

We first describe a crucial identity that links the log risk ratio β_0 and the treatment confounding bridge function $q(A, Z, X)$.

Lemma 2. *Under Assumptions 1, 2, 3 and 5, we have*

$$\beta_0 = \log\left(\frac{E[q(A,Z,X)I(A=1,Y=1)|S=1]}{E[q(A,Z,X)I(A=0,Y=1)|S=1]}\right) \tag{26}$$

Proof. The right hand side of (26) is

$$\begin{aligned}&\log\left(\frac{E[q(A,Z,X)I(A=1,Y=1)|S=1]}{E[q(A,Z,X)I(A=0,Y=1)|S=1]}\right)\\ =&\log\left(\frac{E[q(A,Z,X)|A=1,Y=1,S=1]}{E[q(A,Z,X)|A=0,Y=1,S=1]}\right)+\log\left(\frac{P(Y=1,A=1|S=1)}{P(Y=1,A=0|S=1)}\right)\end{aligned}$$

Note that for $a = 0, 1$,

$$
\begin{aligned}
& E[q(A, Z, X)|A = a, Y = 1, S = 1] \\
=& E\left\{E[q(A, Z, X)|U, X, A = a, Y = 1, S = 1]|A = a, Y = 1, S = 1\right\} \\
\overset{A.2}{=}& E\left\{E[q(A, Z, X)|U, X, A = a]|A = a, Y = 1, S = 1\right\} \\
\overset{A.5}{=}& E\left\{\frac{1}{P(A = a|U, X)}\middle|A = a, Y = 1, S = 1\right\} \\
=& \int \frac{1}{P(A = a|U = u, X = x)} f(u, x|A = a, Y = 1, S = 1)\,\mathrm{d}u\,\mathrm{d}x \\
=& \int \frac{1}{P(A = a|U = u, X = x)} \times \\
& \frac{P(A = a|U = u, X = x)P(Y = 1|A = a, U = u, X = x)P(S = 1|Y = 1, A = a, U = u, X = x)}{P(A = a, Y = 1, S = 1)}\,\mathrm{d}u\,\mathrm{d}x \\
\overset{A.2,A.3}{=}& \int \frac{\exp(\beta_0 a)P(Y = 1|A = 0, U = u, X = x)P(S = 1|Y = 1, U = u, X = x)}{P(A = a, Y = 1, S = 1)}\,\mathrm{d}u\,\mathrm{d}x \\
=& \frac{\exp(\beta_0 a)}{P(A = a, Y = 1, S = 1)} \int P(Y = 1|A = 0, U = u, X = x)P(S = 1|Y = 1, U = u, X = x)\,\mathrm{d}u\,\mathrm{d}x.
\end{aligned}
$$

Therefore, the right-hand side of (26) equals

$$
\begin{aligned}
& \beta_0 + \log\left(\frac{P(A = 0, Y = 1, S = 1)}{P(A = 1, Y = 1, S = 1)}\right) + \log\left(\frac{P(Y = 1, A = 1|S = 1)}{P(Y = 1, A = 0|S = 1)}\right) \\
=& \beta_0 + \log\left(\frac{P(A = 0, Y = 1|S = 1)P(S = 1)}{P(A = 1, Y = 1|S = 1)P(S = 1)}\right) + \log\left(\frac{P(Y = 1, A = 1|S = 1)}{P(Y = 1, A = 0|S = 1)}\right) \\
=& \beta_0
\end{aligned}
$$

□

Let q^* be the function that satisfies (11):

$$
E[q^*(a, Z, X)|A = a, W, X, Y = 0, S = 1] = \frac{1}{P(A = a|U = u, X, Y = 0, S = 1)}.
$$

We introduce an additional regularity condition:

Assumption 11 (Uniform continuity)**.** *For any fixed positive square-integrable function $g(U)$ and a small positive number $0 < \eta < 1$, there exists some $0 < \gamma = \gamma(g, \eta) < 0$ such that $1 - \gamma < \dfrac{E[g_1(U)|A, W, X, Y = 0, S = 1]}{E[g(U)|A, W, X, Y = 0, S = 1]} < \dfrac{1}{1-\gamma}$ a.e. implies $1 - \eta < \dfrac{g_1(U)}{g(U)} < \dfrac{1}{1-\eta}$ a.e., where $g_1(U)$ is a positive square integrable function.*

Assumption 11 requires that the inverse mapping of $g \to E[g(U)|A, W, X, Y = 0, S = 1]$ is sufficiently smooth. By Theorem 2 and Assumption 8, we have

$$
\begin{aligned}
(1-\delta)^3 &< \frac{E[q^*(A, Z, X)|A, W, X, Y = 0, S = 1]}{E[q(A, Z, X)|A, W, X, Y = 0, S = 1]} \\
&= \frac{E\{E[q^*(A, Z, X)|A, W, U, X, Y = 0, S = 1]|A, W, X, Y = 0, S = 1\}}{E\{E[q(A, Z, X)|A, W, U, X, Y = 0, S = 1]|A, W, X, Y = 0, S = 1\}} \\
&\overset{A.6}{=} \frac{E\{E[q^*(A, Z, X)|A, U, X]|A, W, X, Y = 0, S = 1\}}{E\{E[q(A, Z, X)|A, U, X]|A, W, X, Y = 0, S = 1\}} \\
&\overset{A.5}{=} \frac{E\{E[q^*(A, Z, X)|A, U, X]|A, W, X, Y = 0, S = 1\}}{E\{E[\frac{1}{P(A|U,X)}|A, U, X]|A, W, X, Y = 0, S = 1\}} \\
&< \frac{1}{(1-\delta)^3}
\end{aligned}
$$

By Assumption 11, this implies

$$\frac{1-\eta(\delta)}{P(A|U,X)} < E[q^*(A, Z, X)|A, U, X] < \frac{1}{(1-\eta(\delta))P(A|U,X)}. \tag{27}$$

The constant $\eta(\delta)$ is determined by the smoothness of the inverse mapping of $g \mapsto E[g(U)|A, W, X, Y = 0, S = 1]$.

$$E[q^*(A, Z, X)|U = u, X, Y = 0, S = 1] = \frac{1}{P(A = a|U = u, X, Y = 0, S = 1)} \tag{28}$$

for almost all a and u. Let

$$\begin{aligned}\beta_0^* &= \log\left(\frac{E[q^*(A,Z,X)AY|S=1]}{E[q^*(A,Z,X)(1-A)Y|S=1]}\right)\\ &= \log\left(\frac{E[q^*(A,Z,X)|A=1,Y=1,S=1]}{E[q^*(A,Z,X)|A=0,Y=1,S=1]}\right) + \log\left(\frac{P(Y=1,A=1|S=1)}{P(Y=1,A=0|S=1)}\right).\end{aligned}$$

Under mild regularity, the estimator $\widehat{\beta}$ is regular and asymptotically linear for β_0^*, and therefore

$$\widehat{\beta} = \beta_0^* + O_p(1/\sqrt{n}) = \beta_0 + (\beta_0^* - \beta_0) + O_p(1/\sqrt{n}).$$

It suffices to study

$$\beta_0^* - \beta_0 = \log\left(\frac{E[q^*(A,Z,X)|A=1,Y=1,S=1]}{E[q^*(A,Z,X)|A=0,Y=1,S=1]}\right) - \log\left(\frac{E[q(A,Z,X)|A=1,Y=1,S=1]}{E[q(A,Z,X)|A=0,Y=1,S=1]}\right)$$

Notice that

$$\begin{aligned}&E[q^*(A,Z,X)|A=a,Y=1,S=1]\\ =&E\{E[q^*(A,Z,X)|A=a,U,X,Y=1,S=1]|A=a,Y=1,S=1\}\\ \overset{A.4}{=}&E\{E[q^*(A,Z,X)|A=a,U,X]|A=a,Y=1,S=1\}\\ \overset{A.4}{=}&E\{E[q^*(A,Z,X)|A=a,U,X,Y=0]|A=a,Y=1,S=1\}\\ \overset{(27)}{<}&\frac{1}{1-\eta(\delta)}E\left\{\frac{1}{P(A=a|U,X)}\middle|A=a,Y=1,S=1\right\}\\ \overset{A.5}{=}&\frac{1}{1-\eta(\delta)}E\left\{E[q(A,Z,X)|A,U,X]\middle|A=a,Y=1,S=1\right\}\\ \overset{A.4}{=}&\frac{1}{1-\eta(\delta)}E\left\{E[q(A,Z,X)|A,U,X,Y=1,S=1]\middle|A=a,Y=1,S=1\right\}\\ =&\frac{1}{1-\eta(\delta)}E[q(A,Z,X)|A=a,Y=1,S=1]\end{aligned}$$

and similarly,

$$E[q^*(A,Z,X)|A=a,Y=1,S=1] > (1-\eta(\delta))E[q(A,Z,X)|A=a,Y=1,S=1].$$

Therefore, we have

$$1-\eta(\delta) < \frac{E[q^*(A,Z,X)|A=a,Y=1,S=1]}{E[q(A,Z,X)|A=a,Y=1,S=1]} < \frac{1}{1-\eta(\delta)}.$$

We conclude that

$$2\log(1-\eta(\delta)) = \log((1-\epsilon)^2) < \beta_0^* - \beta_0 < \log\left(\frac{1}{(1-\epsilon)^2}\right) = -2\log(1-\epsilon)$$

and thus

$$|\widehat{\beta} - \beta_0| < -2\log(1-\eta(\delta)) + O_p(\frac{1}{\sqrt{n}}).$$

J Regularity conditions and proof of Theorem 3

We denote τ_0^* as the true value of τ such that $q(A, Z, X; \tau_0^*) = q^*(A, Z, X)$. We will give the regularity conditions and proof that $(\widehat{\beta}, \widehat{\tau})$ is a regular and asymptotically linear estimator of (β_0^*, τ_0^*). Here β_0^* and q^* are the biased versions of β_0 and q defined in Appendix I, respectively, although the biases are negligible when the infection is rare. Following Appendix I, $\widehat{\beta}$ is also a regular and asymptotically linear estimator of β_0 if

$$\sup_{a,w,u,x} P(Y = 1|A = a, W = w, U = u, X = x) < \delta_n,$$

Assumption 11 holds and

$$\log(1 - \eta(\delta_n)) = o_p(\frac{1}{\sqrt{n}}).$$

A set of regularity conditions are

R.1 The function $\tau \mapsto q(A, Z, X; \tau)$ is Lipschitz in a neighborhood of τ_0^*; that is, for every τ_1 and τ_2 in a neighborhood of τ_0 and a measurable function $\dot{q}(A, Z, X)$ with $E[\dot{q}(A, Z, X)] < \infty$, we have $\|q(A, Z, X; \tau_1) - q(A, Z, X; \tau_2)\| \leq \dot{q}(A, Z, X)\|\tau_1 - \tau_2\|$;

R.2 $E[q(A, Z, X; \tau_0^*)^2] < \infty$ and $E[m(W, A, X)^2] < \infty$;

R.3 The function $\tau \mapsto q(A, Z, X; \tau)$ is differentiable at τ_0^*. The derivative matrix $\Omega(\beta_0^*, \tau^*)$ is nonsingular;

R.4 $\frac{1}{n}\sum_{i=1}^n G_i(\widehat{\beta}, \widehat{\tau}) = o_p(n^{-1/2})$ and $(\widehat{\beta}, \widehat{\tau}) \xrightarrow{p} (\beta_0^*, \tau_0^*)$.

The condition R.1 and the fact that $\beta \mapsto \exp(-\beta A)$ is Lipschitz in a neighborhood of β_0^* imply the function $(\beta, \tau) \to M_i(\beta, \tau)$ is Lipschitz in a neighborhood of (β_0^*, τ_0^*) for every i. The remaining proof follows Van der Vaart (2000) Theorem 5.21.

K Estimating conditional causal RR in the presence of effect modification by measured confounders

Algorithm 2 below describes a straightforward extension of Algorithm 1 to estimation the conditional vaccine effectiveness $VE(x) = 1 - \exp(\beta_0(x))$ under Assumption 9 and a parametric model $\beta_0(X;\alpha)$ indexed by a finite-dimensional parameter α.

Algorithm 2 Negative control method to estimate conditional vaccine effectiveness from a test-negative design

1: Identify the variables in the data according to Figure 1(c), in particular the NCEs and NCOs.

2: Estimate the treatment confounding bridge function by solving the equation (13) with a suitable parametric model $q^*(A, Z, X; \tau)$ and a user-specified function $m(W, A, X)$. Write $\widehat{\tau}$ as the resulting estimate of τ.

3: Estimate α by solving

$$\frac{1}{n}\sum_{i=1}^{n}(-1)^{1-A_i}c(X_i)q^*(A_i, Z_i, X_i; \widehat{\tau}) \exp(-\beta_0(X_i;\alpha)A_i) = 0 \tag{29}$$

Denote the resulting estimate of α as $\widehat{\alpha}$. The estimated conditional vaccine effectiveness is

$$\widehat{VE}(x) = 1 - \exp(\beta_0(x; \widehat{\alpha})).$$

We describe the large-sample properties of the estimator $(\widehat{\alpha}, \widehat{\tau})$ in the theorem below.

Theorem 5 (Inference of $(\widehat{\alpha}, \widehat{\tau})$). *Under Assumptions 1- 2, 4-5, 6, 9 and suitable regularity conditions listed at the end of this section, the estimator $(\widehat{\alpha}, \widehat{\tau})$ in Algorithm 2, or equivalently, the solution to the estimating equation $\frac{1}{n}\sum_{i=1}^{n}\tilde{G}_i(\alpha,\tau) = 0$ is regular and asymptotically linear with influence function*

$$\widetilde{IF}(\alpha,\tau) = -\left[\tilde{\Omega}(\alpha,\tau)^T\tilde{\Omega}(\alpha,\tau)\right]^{-1}\tilde{\Omega}(\alpha,\tau)^T\tilde{G}_i(\alpha,\tau),$$

where

$$\tilde{G}_i(\alpha,\tau) = \begin{pmatrix} (-1)^{1-A_i} c(X_i) q^*(A_i, Z_i, X_i; \tau) Y_i \exp(-\beta_0(X;\alpha)A_i) \\ (1-Y_i)\left[m(W_i, A_i, X_i) q^*(A_i, Z_i, X_i; \tau) - m(W_i, 1, X_i) - m(W_i, 0, X_i)\right] \end{pmatrix}$$

and

$$\tilde{\Omega}(\alpha,\tau) = \left(E\left[\frac{\partial \tilde{G}_i(\alpha,\tau)}{\partial \alpha^T}\right], E\left[\frac{\partial \tilde{G}_i(\alpha,\tau)}{\partial \tau^T}\right] \right).$$

Here $c(X)$ is a user-specified function of X with the sample dimension as α.

Suppose that in Algorithm 2, one specifies $\beta_0(X;\alpha) = X^T\alpha$, then a natural choice for $c(X)$ is $c(X) = X$. A sandwich estimator of the asymptotic variance of $(\widehat{\alpha}, \widehat{\tau})$ can be deduced from previous derivations. Under Assumption 9, we have shown that one can identify $VE(X)$, however, one may be unable to identify the population marginal VE without an additional assumption. Interestingly, we note that the population marginal risk ratio would remain non-identified even if one had access to a random sample from the target population to inform the marginal distribution of X. Specifically, as shown in Huitfeldt, Stensrud, and Suzuki (2019), the marginal $RR = E[Y(1)]/E[Y(0)]$ can be written as $RR = E[RR(X)|Y(0) = 1]$, i.e. the average risk ratio among subjects who would contract say Influenza had they possibly contrary to fact, not been vaccinated against Influenza. However, the distribution of X within the group $Y(a = 0) = 1$ cannot be identified in presence of unmeasured confounding, thus ruling out identification of the population marginal RR.

Define q^* as before and

$$\beta_0^*(x) = \log\left(\frac{E[q^*(A,Z,x)I(A=1,Y=1)|S=1,X=x]}{E[q^*(A,Z,x)I(A=0,Y=1)|S=1,X=x]}\right).$$

Denote α_0^* as the value of α such that $\beta_0(x;\alpha_0^*) = \beta_0^*(x)$. Below we give the set of regularity conditions such that $(\widehat{\alpha}, \widehat{\tau})$ is a regular and asymptotically linear estimator of (α_0^*, τ_0^*):

R'.1 The function $\tau \mapsto q(A, Z, X; \tau)$ is Lipschitz in a neighborhood of τ_0^* and $\alpha \mapsto \beta_0(X;\alpha)$ is Lipschitz in a neighborhood of α_0^*.

R'.2 $E[q(A, Z, X; \tau_0^*)^2] < \infty$, $E[m(W, A, X)^2] < \infty$, $E[C(X)^2] < \infty$ and $E[\exp(-2\beta_0(X;\alpha_0^*))] < \infty$. ;

R'.3 The function $\tau \mapsto q(A, Z, X; \tau)$ is differentiable at τ_0^* and $\alpha \mapsto \beta_0(X;\alpha)$ is differentiable at α_0^*. The derivative matrix $\tilde{\Omega}(\alpha_0^*, \tau^*)$ is nonsingular;

R'.4 $\frac{1}{n}\sum_{i=1}^{n} \tilde{G}_i(\widehat{\alpha}, \widehat{\tau}) = o_p(n^{-1/2})$ and $(\widehat{\alpha}, \widehat{\tau}) \xrightarrow{p} (\alpha_0^*, \tau_0^*)$.

L Proof of Theorem 1′

Lemma 3. *Under Assumptions 2′, 3′ and 6′, we have*

$$
\begin{aligned}
&P(A = a, Y = y|U, W, X, S = 1) \\
&\quad = \frac{1}{\xi} P(A = a|Y = 0, U, W, X, S = 1) P(Y = y|A = 0, U, W, X, S = 1) \exp(\beta_0' a y)
\end{aligned}
$$

where $\xi = \sum_{a^*, y^*} P(A = a^*|Y = 0, U, W, X, S = 1) P(Y = y^*|A = 0, U, W, X, S = 1) \exp(\beta_0' a^* y^*)$.

Proof. Note that

$$
\begin{aligned}
&\frac{P(Y = 1|A = 1, U, W, X, S = 1) P(Y = 0|A = 0, U, W, X, S = 1)}{P(Y = 1|A = 0, U, W, X, S = 1) P(Y = 0|A = 1, U, W, X, S = 1)} \\
&\overset{A.6'}{=} \frac{P(Y = 1|A = 1, U, X, S = 1) P(Y = 0|A = 0, U, X, S = 1)}{P(Y = 1|A = 0, U, X, S = 1) P(Y = 0|A = 1, U, X, S = 1)} \\
&= \exp(\beta_0') \times \frac{P(S = 1|Y = 1, A = 1, U, X)}{P(S = 1|Y = 0, A = 1, U, X)} \times \frac{P(S = 1|Y = 0, S = 0, U, X)}{P(S = 1|Y = 1, S = 0, U, X)} \\
&\overset{A.2'}{=} \exp(\beta_0') \times \exp(h(U, X)) \times \exp(-h(U, X)) \\
&= \exp(\beta_0')
\end{aligned}
$$

The result follows after Chen (2003). □

To prove Theorem 1′, we need to show

$$E[(-1)^{1-A} c(X) q(A, Z, X) Y \exp(-\beta_a A)|S = 1] = 0$$

It suffices to prove that

$$E[(-1)^{1-A} q(A, Z, X) Y \exp(-\beta_a A)|U, X, S = 1] = 0.$$

The left-hand side is

$$
\begin{aligned}
&\sum_{a,y}\int(-1)^{1-a}y\exp(-\beta_0'a)q(a,z,X)P(A=a,Y=y|U,X,S=1)f(z|U,X,A=a,Y=y,S=1)dz\\
\overset{L.3}{=}&\sum_{a,y}\int(-1)^{1-a}y\exp(-\beta_0'a)q(a,z,X)\times\\
&\frac{1}{\xi}P(A=a|Y=0,U,X,S=1)P(Y=y|A=0,U,X,S=1)\exp(\beta_0'ay)f(z|U,X,A=a,Y=y,S=1)dz\\
=&\sum_{a}\int(-1)^{1-a}\exp(-\beta_0'a)q(a,z,X)\times\\
&\frac{1}{c}P(A=a|Y=0,U,X,S=1)P(Y=1|A=0,U,X,S=1)\exp(\beta_0'a)f(z|U,X,A=a,Y=1,S=1)dz\\
=&\sum_{a}\frac{(-1)^{1-a}}{\xi}P(A=a|Y=0,U,X,S=1)P(Y=1|A=0,U,X,S=1)\times\\
&\int q(a,z,X)f(z|U,X,A=a,Y=1,S=1)dz\\
\overset{A.5'}{=}&\sum_{a}\frac{(-1)^{1-a}}{\xi}P(A=a|Y=0,U,X,S=1)P(Y=1|A=0,U,X,S=1)\times\\
&\frac{1}{P(A=a|Y=0,U,X,S=1)}\\
=&\sum_{a}\frac{(-1)^{1-a}}{\xi}P(Y=1|A=0,U,X,S=1)\\
=&0
\end{aligned}
$$

M Proof of Theorem 2′

$$
\begin{aligned}
&E[\tilde{q}(a,Z,X)|A=a,W,X,Y=0,S=1] \\
=&E\left\{E[\tilde{q}(a,Z,X)|A=a,U,W,X,Y=0,S=1]|A=a,W,X,Y=0,S=1\right\} \\
\overset{A.6'}{=}&E\left\{E[\tilde{q}(a,Z,X)|A=a,U,X,Y=0,S=1]|A=a,W,X,Y=0,S=1\right\} \\
\overset{A.4'}{=}&E\left\{E[\tilde{q}(a,Z,X)|A=a,U,X]|A=a,W,X,Y=0,S=1\right\} \\
\overset{A.5'}{=}&E\left\{\frac{1}{P(A=a|U,X,Y=0,S=1)}|A=a,W,X,Y=0,S=1\right\} \\
=&\int\frac{1}{P(A=a|U=u,X,Y=0,S=1)}f(u|A=a,W,X,Y=0,S=1)du \\
=&\int\frac{1}{P(A=a|U=u,X,Y=0,S=1)}\frac{f(u|W,X,Y=0,S=1)P(A=a|U=u,W,X,Y=0,S=1)}{P(A=a|W,X,Y=0,S=1)}du \\
\overset{A.6'}{=}&\int\frac{1}{P(A=a|U=u,X,Y=0,S=1)}\frac{f(u|W,X,Y=0,S=1)P(A=a|U=u,X,Y=0,S=1)}{P(A=a|W,X,Y=0,S=1)}du \\
=&\frac{1}{P(A=a|W,X,Y=0,S=1)}\int f(u|W,X,Y=0,S=1)du \\
=&\frac{1}{P(A=a|W,X,Y=0,S=1)}
\end{aligned}
$$

N Simulation setting with binary unmeasured confounder

We generate the data of a size $N = 7,000,000$ general population according to Figure 1, with the following distribution:

$$
\begin{aligned}
U &\sim Bernoulli(p_U); \\
Z|U &\sim Bernoulli(p_{0Z} + p_{UZ}U); \\
A|U &\sim Bernoulli(p_{0A} + p_{UA}U); \\
Y|A, Y &\sim Bernoulli(\exp(\eta_{0Y} + \beta_0 A + \eta_{UY}U)); \\
W|U &\sim Bernoulli(p_{0W} + p_{UW}U); \\
D|U &\sim Bernoulli(p_{0D} + p_{UD}U); \\
S|Y, D, W, U &\sim Bernoulli(\max(Y, D, W)(p_{YS} + p_{UYS}U)).
\end{aligned}
$$

In the above data generating process, to mimic a test-negative design platform, we created a binary indicator D for flu-like diseases other than W. The study sample contains subjects with $S = 1$. The distribution of S indicates that only subjects with at least one of Y, D and W equal to one will be recruited into the study sample. We chose the parameters as in Table S1, which resulted in an average study sample size of between around 48,000 and around 52,000.

To obtain the true treatment confounding bridge function, by (6), we have

$$
\sum_z q(a, z)f(z|u, a) = \sum_z q(a, z)f(a|u, z)f(z|u)/f(a|u) = 1/f(a|u)
$$

Table S1: Parameter values of the data generating distribution in the simulation with a binary unmeasured confounder.

Parameter	Value	Parameter	Value
p_U	0.5	p_{0Z}	0.2
p_{UZ}	0.4	p_{0A}	0.2
p_{UA}	0.4	η_{0Y}	log(0.01)
β_0	log(0.2), log(0.5), log(0.7), or 0	η_{UY}	log(0.5)
p_{0W}	0.02	p_{UW}	0.02
p_{0D}	0.02	p_{UD}	-0.015
p_{YS}	0.1	p_{UYS}	0.4

and thus

$$\begin{aligned}&\sum_z q(a,z)f(a|u,z)f(z|u)\\=&q(a,0)[p_{0A}+p_{UA}u]^a[1-(p_{0A}+p_{UA}u)]^{1-a}[1-(p_{0Z}+p_{UZ}u)]+\\&q(a,1)[p_{0A}+p_{UA}u]^a[1-(p_{0A}+p_{UA}u)]^{1-a}(p_{0Z}+p_{UZ}u)\\=&1\end{aligned}$$

for each u,a. We obtain that

$$\begin{pmatrix}q(0,0)\\q(0,1)\end{pmatrix}=\begin{pmatrix}[1-p_{0A}](1-p_{0Z}) & [1-p_{0A}]p_{0Z}\\ [1-(p_{0A}+p_{UA})][1-(p_{0Z}+p_{UZ})] & [1-(p_{0A}+p_{UA})](p_{0Z}+p_{UZ})\end{pmatrix}^{-1}\begin{pmatrix}1\\1\end{pmatrix}$$

$$\begin{pmatrix}q(1,0)\\q(1,1)\end{pmatrix}=\begin{pmatrix}p_{0A}(1-p_{0Z}) & p_{0A}p_{0Z}\\ (p_{0A}+p_{UA})[1-(p_{0Z}+p_{UZ})] & (p_{0A}+p_{UA})(p_{0Z}+p_{UZ})\end{pmatrix}^{-1}\begin{pmatrix}1\\1\end{pmatrix}$$

O Simulation setting with a continuous unmeasured confounder

In simulation studies with a continuous unmeasured confounder, we generate data for a general population of 7,000,000 individuals following the distribution below:

$$
\begin{aligned}
U &\sim Uniform(0,1); \\
X &\sim Uniform(0,1); \\
A|U,X &\sim Binomial(\text{expit}(\mu_{0A} + \mu_{UA}U + \mu_{XA}X)) \\
Z|A,U,X &\sim N(\mu_{0Z} + \mu_{AZ}A + \mu_{XZ}X + \mu_{UZ}U, \sigma_Z^2); \\
W|U,X &\sim N(\mu_{0W} + \mu_{XW}X + \mu_{UW}U, \sigma_W^2); \\
Y|A,U,X &\sim Binomial(\text{expit}(\mu_{0Y} + \beta A + \mu_{UY}U + \mu_{XY}X) + \mu_{UXY}UX) \\
D|U,X &\sim Binomial(\text{expit}(\mu_{0D} + \mu_{XD}X + \mu_{UD}U)) \\
S|U,X,Y,D &\sim \max(Y,D) \times Binomial(\text{expit}(\mu_{0S} + \mu_{XS}X + \mu_{US}U + \mu_{UXS}UX)).
\end{aligned}
$$

We chose the parameter values according to Table S2, which results in an average study sample size of between around 43,000 and around 47,000.

Table S2: Parameter values of the data generating distribution in the simulation with a continuous unmeasured confounder.

Parameter	Value	Parameter	Value
μ_{0A}	-1	μ_{UA}	-1
μ_{XA}	0.25	μ_{0Z}	0
μ_{AZ}	0.25	μ_{XZ}	0.25
μ_{UZ}	4	σ_Z	0.25
μ_{0Y}	log(0.01)	β_0	log(0.2), log(0.5), log(0.7), or 0
μ_{UY}	-2	μ_{XY}	-0.25
μ_{0W}	0	μ_{XW}	0.25
μ_{UW}	2	σ_W	0.25
μ_{0D}	log(0.01)	μ_{XD}	0.25
μ_{UD}	-0.2	μ_{0S}	-1.4
μ_{XS}	0.5	μ_{US}	2
μ_{UXS}	1		

P Logistic regression as a naïve approximate estimator of log risk ratio, ignoring unmeasured confounders

Figure S1 shows a causal diagram of a test-negative design with no unmeasured confounders. Again we assumed selection into the study S is independent of the subjects' treatment status A, given the subjects' infection status Y and other covariates X.

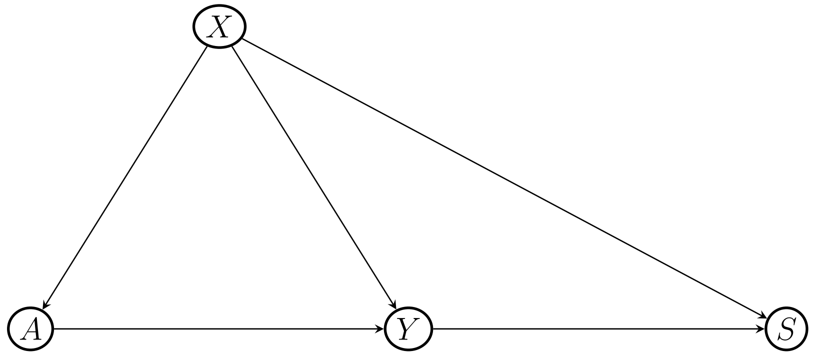

Figure S1: Directed acylic graph of a test-negative design with no unmeasured confounders.

In this scenario, we note that the conditional odds ratio given X in the study population

equals the conditional odds ratio in the general population, i.e.

$$\begin{aligned}
&\frac{P(Y=1|A=1,X,S=1)}{P(Y=1|A=0,X,S=1)} \Bigg/ \frac{P(Y=0|A=1,X,S=1)}{P(Y=0|A=0,X,S=1)} \\
=&\frac{P(Y=1|A=1,X)P(S=1|Y=1,A=1,X)}{P(Y=1|A=0,X)P(S=1|Y=1,A=0,X)} \Bigg/ \frac{P(Y=0|A=1,X)P(S=1|Y=0,A=1,X)}{P(Y=0|A=0,X)P(S=1|Y=0,A=0,X)} \\
\overset{A.2}{=}&\frac{P(Y=1|A=1,X)P(S=1|Y=1,X)}{P(Y=1|A=0,X)P(S=1|Y=1,X)} \Bigg/ \frac{P(Y=0|A=1,X)P(S=1|Y=0,X)}{P(Y=0|A=0,X)P(S=1|Y=0,X)} \\
=&\frac{P(Y=1|A=1,X)}{P(Y=1|A=0,X)} \Bigg/ \frac{P(Y=0|A=1,X)}{P(Y=0|A=0,X)}
\end{aligned}$$

This implies that we may estimate the stratum-specific odds ratio in the general population by fitting a logistic regression model to the study data:

$$P(Y=1|A,X,S=1) = \text{expit}(\gamma_0 + \gamma_X X + \gamma_A A)$$

where $\text{expit}(x) = \exp(x)/[1+\exp(x)]$, and γ_A is the log odds ratio. When the outcome is rare in the general population, i.e. $P(Y=0|A=a,X=x) \approx 1$ for any a, x, the log odds ratio γ_A also approximates the log risk ratio.

Q Simulation for non-rare diseases

To investigate the performance of our method for non-rare diseases, we repeat the simulation with the same setup with binary or continuous confounders. For the simulation with binary confounders, we set η_{0Y} to be $\log(0.20)$; for the simulation with continuous confounders, we set μ_{0Y} to be $\log(0.20)$ – both correspond to an infection risk of 20% for subjects with $A = U = X = 0$.

The results are in Figure S2. While the NC-Oracle estimator remains unbiased and maintains calibrated confidence intervals, the NC estimator is in general biased with under-covered confidence intervals. Notably, the NC estimator is unbiased with calibrated confidence intervals under the null hypothesis where $\beta_0 = 0$. The logistic regression estimator is biased and has unpredictable behavior.

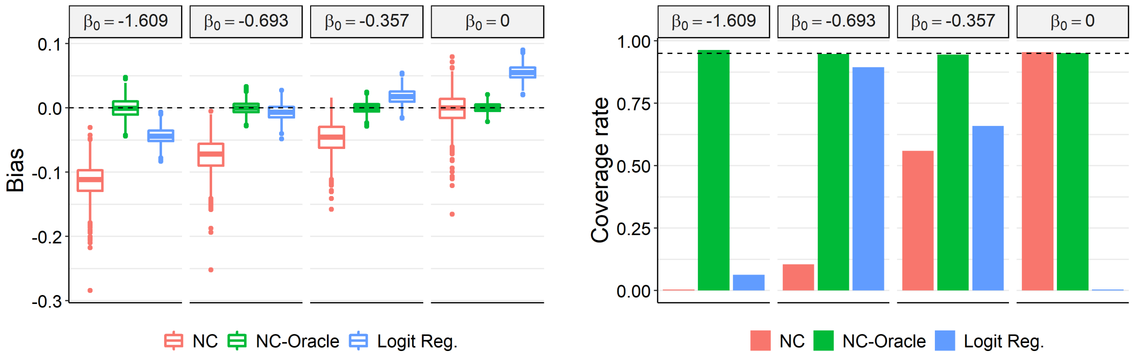

(a)

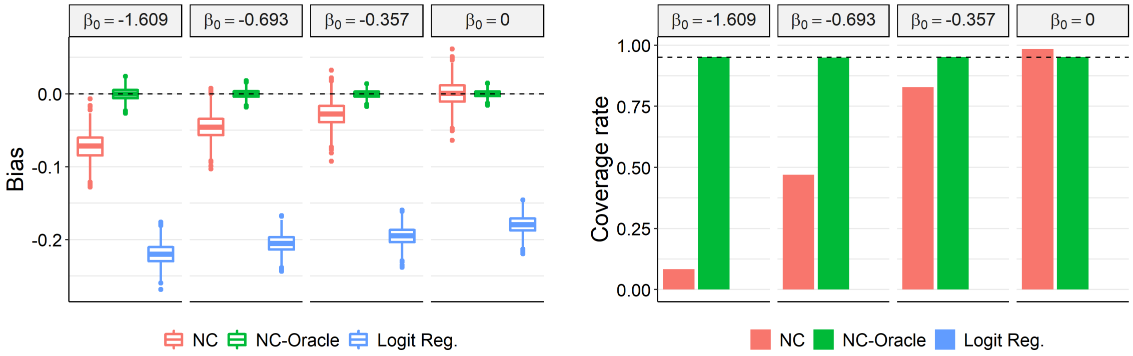

(b)

Figure S2: Bias (left) and coverage rates of 95% confidence interval (right) for the oracle estimator (NC-Oracle), GMM estimator (NC-GMM) and logistic regression (Logit Reg.) with a binary or unmeasured confounder, where η_{0y} or μ_{0Y} is $\log(0.20)$.

R Detailed results of University of Michigan Health System Data analysis

Table S3: Descriptive statistics of University of Michigan Health System COVID-19 Data. Variables were summarized as counts (percentage%).

	Unvaccinated (N=12,672)	Vaccinated (N=39,591)
Vaccine types		
Pfizer-BioNTech	/	20,312 (51.3%)
Moderna	/	10,831 (27.4%)
Johnson & Johnson's Janssen	/	1,409 (3.6%)
Other	/	7,039 (17.8%)
COVID-19 Infection	3,074 (24.2%)	2,774 (7.0%)
NCE: Immunization before Dec 2020	3,854 (30.4%)	18,167 (45.9%)
NCO conditions		
Arm/leg cellulitis	39 (0.3%)	161 (0.4%)
Eye/ear disorder	83 (0.6%)	518 (1.3%)
Gastro-esophageal disease	619 (4.9%)	3,188 (8.0%)
Atopic dermatitis	13 (0.1%)	41 (0.1%)
Injuries	1,033 (8.2%)	3,690 (9.3%)
General adult examination	752 (5.9%)	4,687 (11.8%)
No. of NCO conditions ≥ 1	2,258 (17.8%)	10,355 (26.2%)
Age		
≤ 18	1,683 (13.3%)	2,406 (6.1%)
$\geq 18, < 60$	8,667 (68.4%)	23,495 (59.3%)
≥ 60	2,322 (18.3%)	13,690 (34.6%)
Male	5,357 (42.3%)	16,241 (41.0%)
White	9.012 (71.1%)	30,748 (77.7%)
Charlson score ≥ 3	869 (6.8%)	3,230 (8.2%)

Table S4: Logistic regression of COVID-19 infection on COVID-19 vaccination, the NCE (previous immunization) and other baseline covariates.

	Est.	S.E.	p-value
(Intercept)	-1.30	0.06	<0.001
COVID-19 vaccination	-1.56	0.03	<0.001
Previous Immunization	0.37	0.03	<0.001
Age $\geq$18, $\leq$60	0.29	0.05	<0.001
Age $\geq$60	0.10	0.06	0.099
Male	0.01	0.03	0.750
White	0.32	0.04	<0.001
Charlson score $\geq$3	0.15	0.05	0.005
Calendar month			
April	-0.00	0.05	0.940
May	-0.98	0.06	<0.001
June	-1.79	0.08	<0.001
July	-1.16	0.07	<0.001
August	-0.53	0.05	<0.001
September	-0.40	0.05	<0.001
October	-0.10	0.04	0.026

Table S5: Logistic regression of having at least one NCO conditions on COVID-19 vaccination, the NCE (previous immunization) and other covariates.

	Est.	S.E.	p-value
(Intercept)	-2.32	0.06	<0.001
COVID-19 vaccination	0.28	0.03	<0.001
Previous Immunization	0.63	0.02	<0.001
Age ≥18, ≤60	0.28	0.04	<0.001
Age ≥60	0.76	0.05	<0.001
Male	-0.03	0.02	0.190
White	0.19	0.03	<0.001
Charlson score ≥3	0.38	0.04	<0.001
Calendar month			
April	-0.05	0.04	0.290
May	0.16	0.04	<0.001
June	0.16	0.04	<0.001
July	0.16	0.04	<0.001
August	0.14	0.04	<0.001
September	-0.03	0.04	0.360
October	0.04	0.04	0.309
